# Fulvestrant plus vandetanib versus placebo for the treatment of patients with metastatic breast cancer resistant to aromatase inhibitor therapy (FURVA): a multicentre, Phase 2, randomised controlled trial

**DOI:** 10.1038/s44276-023-00016-8

**Published:** 2023-09-14

**Authors:** Mark Beresford, Angela Casbard, Zoe Hudson, Margherita Carucci, Kate Ingarfield, Julia Gee, Joanna Smith, Terri Kitson, Fouad Alchami, Tracie-Ann Madden, Larrie Hayward, David Hwang, Saiqa Spensley, Simon Waters, Duncan Wheatley, Robert H. Jones

**Affiliations:** 1 Royal United Hospitals Bath NHS Foundation Trust, Bath, UK; 2 Centre for Trials Research, Cardiff University, Cardiff, UK; 3 School of Pharmacy & Pharmaceutical Sciences, Cardiff University, Cardiff, UK; 4 Department of Cellular Pathology, Cardiff & Vale University Health Board, Cardiff, UK; 5 Edinburgh Cancer Centre, NHS Lothian, Edinburgh, UK; 6 Royal Devon and Exeter Foundation NHS Trust, Exeter, UK; 7 Musgrove Park Hospital, Taunton and Somerset NHS Foundation Trust, Taunton, UK; 8 Velindre Cancer Centre, Velindre University NHS Trust, Cardiff, UK; 9 Royal Cornwall Hospital, Royal Cornwall Hospitals NHS Trust, Cornwall, UK

## Abstract

**Background:**

FURVA, a randomised, double-blind Phase II trial, investigated whether the addition of vandetanib to fulvestrant improved progression-free survival (PFS) in patients with an aromatase inhibitor(AI)-resistant advanced breast cancer.

**Methods:**

Postmenopausal women with oestrogen receptor-positive (ER+ve)/HER2-negative advanced breast cancer, who experienced disease progression on an AI, were randomised (1:1) to fulvestrant 500 mg (Q28) with vandetanib 300 mg od (f + v) or placebo (f + p) until disease progression or discontinuation. The primary endpoint was PFS; secondary endpoints included overall survival (OS) and the influence of REarranged during Transfection (RET) signalling on outcomes.

**Results:**

In total, 165 participants were randomised to f + v (*n* = 80) or f + p (*n* = 85). Median PFS was 5.5 months (m) for f + v compared to 5.5 m for f + p (hazard ratio (HR) 0.88; 95% CI: 0.62–1.23; *P* = 0.22). Unexpectedly, high total RET expression was associated with a PFS advantage of 8.87 m vs 3.94 with low RET (HR 0.493: 95% CI 0.32–0.77; *P* = 0.002) independent of the treatment arm, supported by an OS advantage 21.95 m vs 18.04 (HR 0.584; 95% CI 0.34–1.00; *P* = 0.051) in the high-RET group.

**Conclusion:**

The addition of vandetanib to fulvestrant does not improve PFS. However, high total RET expression was associated with improved PFS, suggesting RET may have a prognostic role in patients treated with fulvestrant.

**Clinical trial registration:**

ClinicalTrials.gov, NCT02530411.

## Background

Endocrine therapy is the treatment of choice for women with oestrogen receptor-positive (ER+ve)/HER2-negative breast cancer, but resistance frequently develops in almost all such women with advanced disease. Over the last 10 years, the treatment landscape of ER+/HER2− breast cancers has changed significantly with the use of CDK4/6 inhibitors [1] and targeted therapies, such as those targeting the PI3K pathway alongside endocrine agents [2, 3].

RET (REarranged during Transfection) is a receptor tyrosine kinase, which along with its ligands and co-receptors (glial cell line-derived neurotrophic factor receptors, GFRαs) acts via the PI3K and MAPK pathway that can activate the ER. High-RET expression has been shown to correlate with shorter metastasis-free and overall survival in breast cancer [4]. In cell line models, RET and its signalling has been demonstrated to be upregulated in endocrine-resistant cells [5–7] and is closely associated with ER activation in an oestrogen-independent manner [8], making its upregulation a possible mechanism of endocrine resistance and therefore an attractive target for inhibition.

Vandetanib is an oral inhibitor of vascular endothelial growth factor receptor 2 (VEGFR-2), epidermal growth factor receptor (EGFR) and RET tyrosine kinases. Vandetanib is licenced for use in medullary thyroid cancer where RET activation is predominately driven by point mutations. It has been used in small-scale studies in breast cancer, demonstrating safety and tolerability but these were not powered to show clinical efficacy [9, 10]. Examining safety data from these clinical trials suggests that higher doses of vandetanib could be used, potentially increasing the likelihood of a clinical benefit.

Scientific rationale for further examining the role of vandetanib in the management of patients with ER+ve/HER2–ve metastatic breast cancer is supported by cell line experiments demonstrating that breast cancer cell growth (along with key downstream kinase activity and ER cross-talk) is significantly inhibited by vandetanib, particularly in the setting of endocrine-resistant compared to endocrine responsive cells [5]. RET targeting can furthemore improve sensitivity to endocrine strategies in some ER+ve models [8, 11, 12]. Fulvestrant is a selective oestrogen receptor downregulator which is active in the setting of ER+ve/HER2–ve disease [13] and is an active agent in endocrine-resistant disease [14, 15].

FURVA assessed the efficacy and safety of vandetanib plus fulvestrant in postmenopausal women with ER+ve, HER2–ve advanced/metastatic breast cancer resistant to endocrine therapies. It also explored the influence of RET signalling pathway expression on vandetanib plus fulvestrant outcome.

## Methods

### Study design and participants

In this investigator-initiated, multicentre, randomised (1:1), double-blind, placebo-controlled, Phase II trial (ClinicalTrials.gov: NCT02530411; EudraCT: 2014-001208-23), patients were enrolled from 19 UK centres (Supplementary Table S2). The protocol is in the Supplementary Material.

Postmenopausal women with histologically confirmed ER+ve, HER2–ve locally advanced or metastatic breast cancer not amenable to surgical resection but suitable for endocrine therapy were enrolled. Patients who experienced disease progression whilst receiving an aromatase inhibitor (AI), or relapsed on an AI in the adjuvant setting, were eligible. Up to three previous lines of endocrine treatment and one line of chemotherapy for advanced breast cancer were permitted. Participants were categorised as having either primary or secondary resistance to aromatase inhibitor therapy. Primary resistance was defined as either disease relapse during or within 6 months of completing aromatase inhibitor treatment in the adjuvant setting, or disease progression within 6 months of starting aromatase inhibitor treatment and no response to aromatase inhibitor treatment in the metastatic setting. Secondary resistance was defined as disease relapse more than 6 months after completion of aromatase inhibitor treatment in the adjuvant setting, or disease progression following achievement of clinical benefit with aromatase inhibitor treatment in the metastatic setting. Patients could have measurable or non-measurable disease by Response Evaluation Criteria in Solid Tumours, version 1.1 (RECIST). All patients were required to have a minimum 12-week life expectancy, an ECOG (Eastern Cooperative Oncology Group) status of 0–2 and adequate bone marrow and organ function as defined in the protocol.

Key exclusion criteria were previous treatment with fulvestrant or RET pathway inhibitors, rapidly progressive visceral disease or abnormal cardiac function. Full inclusion and exclusion criteria are provided in the Supplementary Material (pages 28–31).

### Randomisation and masking

Participants were randomly assigned (1:1) to receive fulvestrant plus vandetanib or fulvestrant plus placebo. Randomisation was performed centrally, using minimisation with a 20% random element [16]. Minimisation factors included measurable versus non-measurable disease, primary versus secondary AI resistance, and the presence of liver and/or lung metastases. An interactive web-response system (IWRS) was used to assign patients’ treatment kits and trial number. Vandetatnib tablets and matching placebo had identical packaging, labelling, appearance, and administration schedules. Participants, investigators, study site staff (including radiologists and IHC assessors) and the sponsor were masked to treatment allocation until the database lock. Unblinding was permitted in case of emergency.

### Procedures

Fulvestrant 500 mg was administered on day 1 of every cycle as two intramuscular injections into each buttock, with an additional loading dose at cycle 1 day 15. Vandetanib or matching placebo was given orally twice daily with a 300 mg dose, but patients with renal impairment (defined as creatinine clearance 30–50 ml/min) started on a 200 mg dose. Fulvestrant and vandetanib were manufactured and provided by AstraZeneca and distributed by Fisher Clinical Services (Horsham, UK) throughout the trial, though AstraZeneca divested vandetanib to Genzyme in July 2015.

Participants completed drug diaries, which were reviewed at each study visit to aid data collection. Participants continued to receive study treatment until disease progression, development of unacceptable toxicities, loss to follow-up, or withdrawal of consent.

Toxicities suspected to be related to vandetanib were managed by dose interruption or dose reduction to 200 mg, then to 100 mg at the same schedule, then to 100 mg every other day. In participants with renal impairment, related toxicities were managed by dose interruption or dose reduction to 100 mg at the same schedule, then to 100 mg every other day. Repeated dose interruptions and continuous interruption of up to 28 days were allowed. Dose reduction of fulvestrant to 250 mg was allowed after discussion with the Chief Investigators.

Cross-sectional imaging of chest, abdomen and, if clinically indicated, pelvis was performed up to 28 days before randomisation to confirm eligibility, repeated on weeks 8, 16 and 24 and at 12 weekly intervals, thereafter until disease progression. Scans were assessed by local radiologists as per RECIST v1.1 to determine tumour response and date of progression.

The incidence and severity of adverse events (AEs) and serious adverse events (SAEs) were recorded throughout the study period. AEs were classified according to the National Cancer Institute Common Terminology Criteria for Adverse Events version 4.03.

### RET expression by immunohistochemistry (IHC)

Patients’ archival tumour tissue samples were requested from all participants at the point of randomisation to the trial, but only 115 were made available: 52 (45%) samples from the participants in the treatment arm and 63 (55%) from the participants in the placebo arm. All 115 tissue samples were tested for RET expression. Since antibodies for IHC assays previously reported to assess RET expression were no longer in production, a new assay was designed and optimised for the purposes of this study [4, 17]. A recombinant IgG anti-RET rabbit monoclonal antibody was selected (Ab134100, Abcam, Cambridge, UK). Slides containing 5um sections of formalin-fixed, paraffin-embedded (FFPE) tissue were dewaxed and rehydrated using xylene and through ethanol dilutions. This was followed by heat-mediated antigen retrieval, the use of an endogenous peroxidase block, and a subsequent protein-blocking step. The primary antibody was diluted to 1:100 and applied directly to slides which were then incubated at 23 °C overnight. After washes, an HRP-labelled polymer secondary antibody system (Envision+ K4009, Agilent Technologies, CA, USA) was applied for 60 min before finally DAB chromogen/substrate detection (K3468, Agilent Technologies, CA, USA) and counterstain was used to visualise the expression of RET. The assay was pre-validated using a series of clinical samples, FFPE-pelleted cell line controls (high-RET expression in T47D cells and low/negative expression in HeLa cells) and also IgG isotype controls (ab172730, Abcam, Cambridge UK). Internal clinical sample positive controls were used alongside each batch of samples for FURVA. Total RET expression (tRET) was assessed by two scorers ZH and FA using the h-score method [4, 18]. An optimal cut point for high/low tRET expression was determined using MaxStat methodology [17] using the package Survminer [19] in R Studio.

### Outcomes

The primary endpoint was investigator-assessed progression-free survival (PFS), defined as the time from randomisation to the first documented progression (according to RECIST v1.1) or death from any cause regardless of whether the participant withdrew from the study therapy or received another anti-cancer therapy prior to progression. Participants who had not died or whose disease had not progressed at the time of the analysis were assessed at the date of their last evaluable RECIST assessment. If the participant had no evaluable visits or had no baseline data, they were censored at Day 1, unless they died within two visits of baseline.

Secondary endpoints were overall survival (OS), defined as the time from randomisation to death from any cause (participants still alive were censored at the date last seen); objective response rate (ORR), defined as the proportion of participants with a complete or partial response, according to RECIST; clinical benefit rate (CBR), defined as the proportion of participants with an objective response or stable disease lasting 24 weeks or longer from the point of randomisation; duration of response, defined as the time from first documented objective response to the first documented progression or death. Also evaluated were tolerability and feasibility, as shown by the number of participants discontinuing or requiring dose modifications, and the frequency and severity of AEs.

### Statistical analyses

The sample size was calculated for a Phase 2 screening design, based on a primary outcome of PFS, a hazard ratio of 0.65, 90% power, a one-sided significance of 20% and assuming 10% loss to follow-up. Assuming an estimated PFS in the control arm of 5.4 months, 98 events and 120 patients were required. It was expected that at least 30% of participants would have tRET overexpression [4, 18], and these tumours may respond better to vandetanib treatment. With 160 participants, a subgroup analysis was planned of approximately 50 participants with tRET overexpression to be evaluated for activity, and to detect a hazard ratio (HR) of 0.5 in this subgroup, keeping all other sample size parameters the same.

PFS analyses were done on an intention-to-treat basis, including all participants. Safety analyses included all participants who had received at least one dose of the study drug. Event time distributions were estimated with the Kaplan–Meier method. PFS was compared with a one-sided unadjusted log-rank test (the primary analysis). Cox regression was used to estimate HRs with confidence intervals and *P* values; and to adjust the estimates for the randomisation minimisation variables. OS was analysed in the same way as PFS. The proportion of patients with ORR and CBR was summarised by the trial arm and analysed using logistic regression.

Analyses of PFS, OS, ORR and CBR were also conducted by tRET status, as prespecified in the protocol.

Analyses were done using Stata (Version 16) [20].

## Results

### Recruitment

**Fig. 1:**
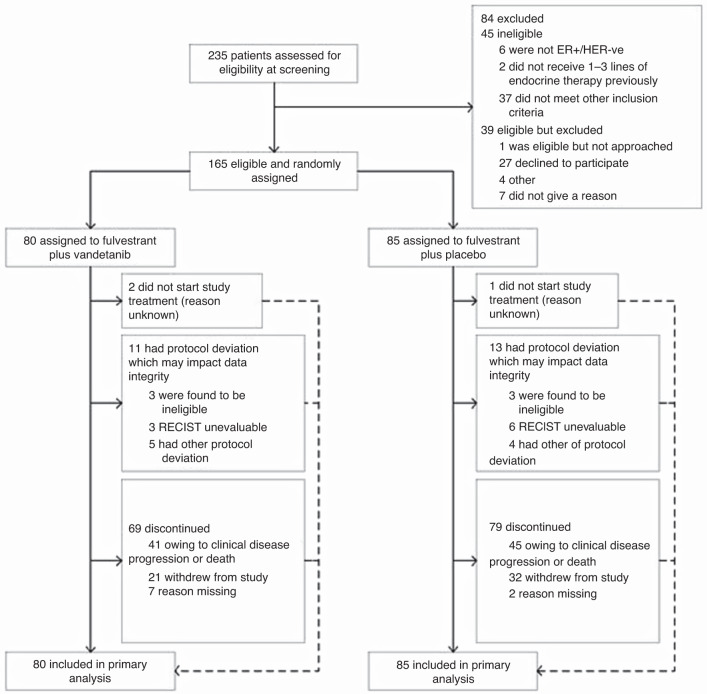
Consort flow diagram of patient eligibility and assignment. Consort diagram.

Between April 2015 and October 2017, 165 participants were randomly assigned in FURVA to receive fulvestrant plus vandetanib (*n* = 80; vandetanib arm), or fulvestrant plus placebo (*n* = 85; placebo arm). The CONSORT flow diagram is presented in Fig. 1.

All participants were included in both primary efficacy and safety analyses. Participants were followed-up until all had had at least six months follow-up and the minimum 98 disease progression events required for analysis were confirmed. Median PFS follow-up was 5.5 months (95% CI 3.7–8.0). Treatment arms were well-balanced for baseline characteristics (Table 1).

**Table 1:** Baseline characteristics by treatment group.

	Fulvestrant + vandetanib	Fulvestrant + placebo
	*N* = 80	*N* = 85
Age—*n*, median (IQR)	80, 64.5 (55.0, 70.5)	85, 65.0 (58.0, 72.0)
Weight—median (IQR)	71.8 (63.0, 82.0)	73.6 (64.5, 83.5)
ECOG status—*N* (%)		
0	55 (69%)	50 (59%)
1	24 (30%)	29 (34%)
2	1 (1%)	4 (5%)
3	0 (0%)	0 (0%)
4	0 (0%)	0 (0%)
Not done	0 (0%)	2 (2%)
Histopathological subtype		
Invasive ductal carcinoma	56 (70%)	66 (79%)
Invasive lobular cancer	16 (20%)	10 (12%)
Other	8 (10%)	8 (10%)
Stage		
III inoperable	3 (4%)	2 (2%)
IV	77 (96%)	83 (98%)
Brain metastases		
Yes	1 (1%)	0 (0%)
No	79 (99%)	85 (100%)
Liver metastases		
Yes	30 (38%)	33 (39%)
No	50 (63%)	52 (61%)
Lung metastases		
Yes	30 (38%)	34 (40%)
No	50 (63%)	51 (60%)
Bone metastases		
Yes	57 (71%)	61 (72%)
No	23 (29%)	24 (28%)
Lymph nodes		
Yes	35 (44%)	33 (39%)
No	45 (56%)	52 (61%)
Other metastases		
Yes	27 (34%)	19 (22%)
No	53 (66%)	66 (78%)
Bone-only disease		
Yes	12 (15%)	17 (20%)
No	68 (85%)	68 (80%)
RECIST measurable disease—*N* (%)		
Yes	61 (76%)	60 (71%)
No	19 (24%)	25 (29%)
Primary or secondary AI resistance—*N* (%)		
Primary	12 (15%)	14 (16%)
Secondary	68 (85%)	71 (84%)

No patients previously received CDK4/6 inhibitors.

### Efficacy

**Fig. 2:**
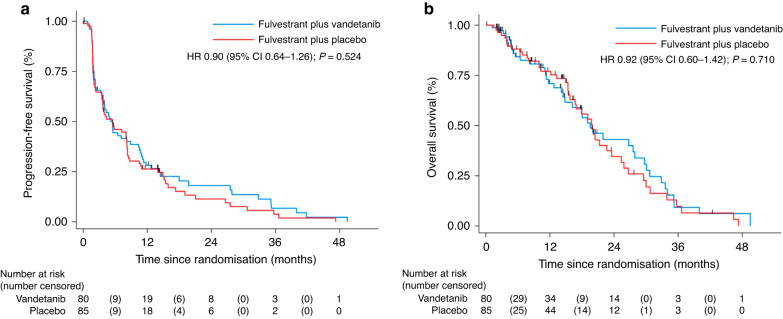
Kaplan-Meier curves. Progression-free survival (**a**) and overall survival (**b**).

At the time of primary analysis, there had been 138 events, 65/80 (81%) in the vandetanib arm compared with 73/85 (86%) in the placebo arm. Median PFS in the vandetanib arm and the placebo arm were 5.5 months (95% CI 3.6–8.9) vs 5.5 months (95% CI 3.5–8.1), respectively, giving an unadjusted hazard ratio (HR) of 0.88 (95% CI 0·63–1.23; two-sided *P* = 0.220) and adjusted HR 0.90 (95% CI 0·64–1.26; two-sided *P* = 0·524; Fig. 2).

A total of 121 (73%) of participants had measurable disease, of whom 113 had evaluable RECIST assessments during follow-up. The ORR in participants with measurable disease was 7% (4/55) in the vandetanib arm compared with 10% (6/59) in the placebo arm (odds ratio 0.71 [95% CI 0.19–2.65]; two-sided *P* = 0.607). The median duration of response was 1.8 months (95% CI 1.8–1.9) and 4.6 months (95% CI 2.7–6.5) for participants in the vandetanib and placebo arms, respectively. Including all participants, the clinical benefit rate was 28% (22/80) in the vandetanib arm vs 35% (30/85) in the placebo arm (OR 0.70 [95% CI 0.36–1.35]; two-sided *P* = 0.282).

Median follow-up for overall survival was 6.5 months, ranging from 0.1 to 42 months, for those who were alive at the point of analysis. In total, 86 death events were recorded. The median OS was 19.5 months (95% CI 14.5–27.9) and 19.9 months (95% CI 15.7–23.6) for the vandetanib and placebo arms, respectively with a HR of 0.92 (0·60–1·42; two-sided *P* = 0·71; Fig. 2).

**Fig. 3:**
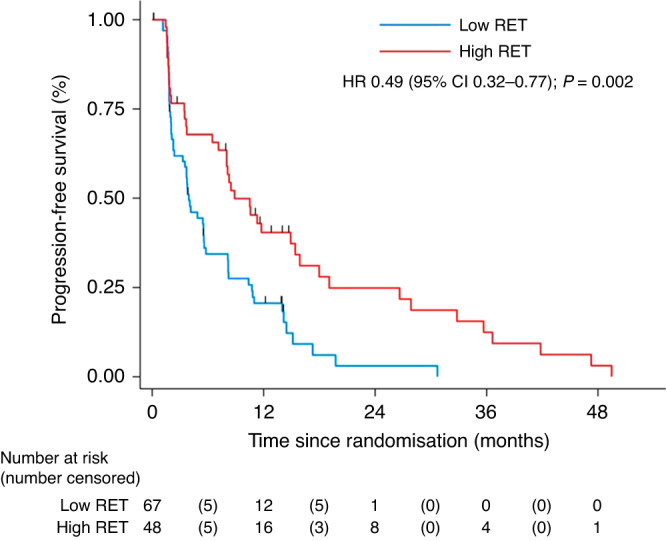
Progression-free survival by RET.

The influence of tRET signalling on participant outcomes was a prespecified exploratory endpoint of the trial, and expression of tRET by IHC was used as an indicator of this. In the RET analysis, a tRET h-score of >166 was defined as RET overexpression using MaxStat methodology [17]. Overall, 42% (48/115) of participants had tumours with high-RET expression, compared to 58% (67/115) without; 44% of participants in the vandetanib group had tumours with high-RET expression compared to 40% in the placebo group. Participants who had tumours with high tRET expression had a median PFS of 8.9 months (95% CI 7.1–15.4) compared to 3.9 months (95% CI 2.4–5.6) in low expressors (HR 0.49 [95% CI 0.31–0.77]) indicating high expression was associated with improved outcomes in FURVA. This was also reflected in the OS analysis which favoured those with tumours of high tRET expression (HR 0.58 [95% CI 0.34–1.00]). However, a test for interaction between RET expression and the treatment group did not demonstrate that the treatment effect was altered by RET expression (Fig. 3 and Supplementary Fig. S1). Thus, the favourable effect of high tRET expression on outcome was seen irrespective of whether participants received fulvestrant plus vandetanib or fulvestrant plus placebo. Overall baseline characteristics were well-balanced between the RET high and low groups, although there were some differences in the site of metastatic disease (Table 2).

**Table 2:** Baseline characteristics by RET.

	HIGH tRET		LOW tRET	
	Fulvestrant + vandetanib	Fulvestrant + placebo	Fulvestrant + vandetanib	Fulvestrant + placebo
	*N* = 23	*N* = 25	*N* = 29	*N* = 38
Age—*n*, median (IQR)	68.0 (58.0, 76.0)	69.0 (60.0, 72.0)	62.0 (55.0, 70.0)	65.0 (56.0, 73.0)
Weight—median (IQR)	72.0 (67.0, 82.9)	71.9 (63.0, 86.1)	66.3 (58.0, 79.7)	74.6 (64.2, 83.5)
ECOG status—*N* (%)				
0	18 (78%)	12 (48%)	19 (66%)	24 (63%)
1	5 (22%)	13 (52%)	10 (34%)	10 (26%)
2	0 (0%)	0 (0%)	0 (0%)	2 (5%)
3	0 (0%)	0 (0%)	0 (0%)	0 (0%)
4	0 (0%)	0 (0%)	0 (0%)	0 (0%)
Not done	0 (0%)	0 (0%)	0 (0%)	2 (5%)
Brain metastases				
Yes	0 (0%)	0 (0%)	1 (3%)	0 (0%)
No	23 (100%)	25 (100%)	28 (97%)	38 (100%)
Liver metastases				
Yes	7 (30%)	9 (36%)	12 (41%)	14 (37%)
No	16 (70%)	16 (64%)	17 (59%)	24 (63%)
Lung metastases				
Yes	6 (26%)	9 (36%)	15 (52%)	16 (42%)
No	17 (74%)	16 (64%)	14 (48%)	22 (58%)
Bone metastases				
Yes	18 (78%)	16 (64%)	19 (66%)	25 (66%)
No	5 (22%)	9 (36%)	10 (34%)	13 (34%)
Lymph nodes				
Yes	9 (39%)	10 (40%)	14 (48%)	18 (47%)
No	14 (61%)	15 (60%)	15 (52%)	20 (53%)
Other metastases				
Yes	9 (39%)	7 (28%)	8 (28%)	7 (18%)
No	14 (61%)	18 (72%)	21 (72%)	31 (82%)
Bone-only disease				
Yes	5 (22%)	5 (20%)	5 (17%)	6 (16%)
No	18 (78%)	20 (80%)	24 (83%)	32 (84%)
RECIST of target tumour lesions, measurable disease—*N* (%)				
Yes	16 (70%)	17 (68%)	22 (76%)	29 (76%)
No	7 (30%)	8 (32%)	7 (24%)	9 (24%)
Primary or secondary AI resistance—*N* (%)				
Primary	0 (0%)	5 (20%)	5 (17%)	7 (18%)
Secondary	23 (100%)	20 (80%)	24 (83%)	31 (82%)

### Study drug administration and tolerability

The median duration of fulvestrant treatment was 2.8 months (range = 0–45.4) in the vandetanib arm compared with 3.7 months (range = 0.5–33.3) in the placebo arm. The median duration of vandetanib itself was shorter at 1.8 months (range = 0–18.5). The median duration of placebo administration was 3.7 months (range = 0–33.3).

Overall, 28 participants (35%) had a vandetanib dose reduction compared with 4 (5%) in the placebo arm; more specifically, 17 had one, eight had two, and three had more than two vandetanib dose reductions. The most common toxicities leading to a dose reduction of vendetanib were renal dysfunction (7 participants), diarrhoea (5 participants), and electrocardiogram (ECG) changes (5 participants). No participants had a fulvestrant dose reduction.

All grade toxicities affecting more than 10% of the study population, irrespective of causality, are presented in Table 3 (full toxicity data in Supplementary Table S1). The proportion of participants experiencing grade 3–5 AEs was 37/80 (46%) in the vandetanib arm and 24/85 (28%) in the placebo arm. Two participants in the vandetanib group had grade 5 toxicities (one intracranial haemorrhage and one pneumonia), which were both classed as unrelated to the investigational therapy. All cases of severe diarrhoea and vomiting were no more than a grade 3. Most cases of rashes were grade 1–3, but one participant in the vandetanib arm had a grade 4 rash. Serious adverse reactions in the vandetanib arm were: confusional state (*n* = 1), dehydration (*n* = 1), diarrhoea (*n* = 1), infection (*n* = 1), left ventricular dysfunction (*n* = 1), photosensitivity reaction (*n* = 1), seizure (*n* = 1) and toxic epidermal necrolysis (*n* = 1).

**Table 3:** Reported toxicities by randomised group affecting ≥10% of participants, irrespective of causality, in order of magnitude.

	Grade reported, *N* (%)									
	Fulvestrant plus vandetanib (*n* = 80)					Fulvestrant plus placebo (*n* = 85)				
	1	2	3	4	5	1	2	3	4	5
Patients with any AE (worst-reported grade)	8 (10%)	34 (42.5%)	34 (42.5%)	1 (1.3%)	2 (2.5%)	22 (25.9%)	38 (44.7%)	18 (21.2%)	6 (70.6%)	0 (0.0%)
Fatigue/lethargy	23 (28.8%)	34 (42.5%)	4 (5.0%)	0 (0.0%)	0 (0.0%)	38 (44.7%)	16 (18.8%)	3 (3.5%)	0 (0.0%)	0 (0.0%)
Diarrhoea	32 (40.0%)	15 (18.8%)	9 (11.3%)	0 (0.0%)	0 (0.0%)	22 (25.9%)	2 (2.4%)	1 (1.2%)	0 (0.0%)	0 (0.0%)
Nausea	33 (41.3%)	11 (13.8%)	1 (1.3%)	0 (0.0%)	0 (0.0%)	31 (36.5%)	7 (8.2%)	1 (1.2%)	0 (0.0%)	0 (0.0%)
Rash/pruritis/dry skin	33 (41.3%)	19 (23.8%)	4 (5.0%)	1 (1.3%)	0 (0.0%)	23 (27.1%)	2 (2.4%)	0 (0.0%)	0 (0.0%)	0 (0.0%)
Liver function test	34 (42.5%)	9 (11.3%)	2 (2.5%)	0 (0.0%)	0 (0.0%)	24 (28.2%)	2 (2.4%)	2 (2.4%)	1 (1.2%)	0 (0.0%)
Hypertension	4 (5.0%)	19 (23.8%)	6 (7.5%)	0 (0.0%)	0 (0.0%)	2 (2.4%)	6 (7.1%)	3 (3.5%)	0 (0.0%)	0 (0.0%)
Headache	16 (20.0%)	4 (5.0%)	1 (1.3%)	0 (0.0%)	0 (0.0%)	19 (22.4%)	2 (2.4%)	0 (0.0%)	0 (0.0%)	0 (0.0%)
Dyspnoea	13 (16.3%)	4 (5.0%)	1 (1.3%)	0 (0.0%)	0 (0.0%)	11 (12.9%)	6 (7.1%)	2 (2.4%)	0 (0.0%)	0 (0.0%)
Arthralgia	13 (16.3%)	2 (2.5%)	0 (0.0%)	0 (0.0%)	0 (0.0%)	15 (17.6%)	8 (9.4%)	0 (0.0%)	0 (0.0%)	0 (0.0%)
Vomiting	20 (25.0%)	4 (5.0%)	0 (0.0%)	0 (0.0%)	0 (0.0%)	12 (14.1%)	2 (2.4%)	0 (0.0%)	0 (0.0%)	0 (0.0%)
Constipation	13 (16.3%)	6 (7.5%)	0 (0.0%)	0 (0.0%)	0 (0.0%)	12 (14.1%)	4 (4.7%)	0 (0.0%)	0 (0.0%)	0 (0.0%)
Cough	12 (15.0%)	5 (6.3%)	0 (0.0%)	0 (0.0%)	0 (0.0%)	8 (9.4%)	5 (5.9%)	0 (0.0%)	0 (0.0%)	0 (0.0%)
Decrease appetite	15 (18.8%)	9 (11.3%)	0 (0.0%)	0 (0.0%)	0 (0.0%)	12 (14.1%)	5 (5.9%)	0 (0.0%)	0 (0.0%)	0 (0.0%)
Hot flush	16 (20.0%)	3 (3.8%)	0 (0.0%)	0 (0.0%)	0 (0.0%)	13 (15.3%)	1 (1.2%)	0 (0.0%)	0 (0.0%)	0 (0.0%)
Injection site reactions	15 (18.8%)	2 (2.5%)	0 (0.0%)	0 (0.0%)	0 (0.0%)	13 (15.3%)	3 (3.5%)	0 (0.0%)	0 (0.0%)	0 (0.0%)
Back pain	5 (6.3%)	7 (8.8%)	0 (0.0%)	0 (0.0%)	0 (0.0%)	13 (15.3%)	3 (3.5%)	0 (0.0%)	0 (0.0%)	0 (0.0%)
Mucosal inflammation	9 (11.3%)	6 (7.5%)	0 (0.0%)	0 (0.0%)	0 (0.0%)	9 (10.6%)	0 (0.0%)	0 (0.0%)	0 (0.0%)	0 (0.0%)
Dizziness	9 (11.3%)	2 (2.5%)	0 (0.0%)	0 (0.0%)	0 (0.0%)	5 (5.9%)	1 (1.2%)	0 (0.0%)	0 (0.0%)	0 (0.0%)
Musculoskeletal pain	13 (16.3%)	6 (7.5%)	0 (0.0%)	0 (0.0%)	0 (0.0%)	15 (17.6%)	6 (7.1%)	0 (0.0%)	0 (0.0%)	0 (0.0%)
Respiratory tract infection/pneumonia	1 (1.3%)	7 (8.8%)	2 (2.5%)	0 (0.0%)	1 (1.3%)	1 (1.2%)	8 (9.4%)	2 (2.4%)	0 (0.0%)	0 (0.0%)

## Discussion

Multiple potential mechanisms of endocrine resistance in breast cancer have been defined pre-clinically [7], including increased glial cell line-derived neurotrophic factor receptor (GFR)-coupled RET signalling. A number of in vitro models have indicated targeting RET can inhibit the growth of endocrine-resistant cells. In addition, vandetanib shows evidence of a dose response-dependent tumour regression in mouse models [5, 8, 11, 12, 14, 15]. In contrast, two previously published randomised trials testing the efficacy of 100 mg dose of vandetanib alongside chemotherapy or fulvestrant [18] found no improvement in clinical or laboratory outcomes. However, there were a number of concerns about these two clinical studies. The chemotherapy trial was small (*n* = 64) and insufficiently powered to observe differences. The fulvestrant trial was predominantly in patients with bone metastases and used urinary N-telopeptide NTx bone markers as the primary outcome measure rather than clinical outcomes. In this study, only 61 participants had RECIST measurable disease, compared to 121 in FURVA, further compromising the ability to draw any meaningful conclusions from the clinical outcome data. Neither study performed a subgroup analysis according to tRET status and in both cases, the 100 mg vandetanib dose used might have been subtherapeutic. Data from a number of trials have shown that a vandetanib dose of 300 mg is tolerable and optimal for efficacy and therefore this dose was chosen for the FURVA study [21–24]. Moreover, neither of the trials studied an AI-resistant cohort, and so FURVA represents the first AI-resistant patient cohort to be studied with vandetanib. However, in the intention-to-treat population, no difference in median PFS in the vandetanib arm was seen compared to the placebo arm (5.5 months in both cases). There was no discernible difference in OS between the two arms with median of 19.5 and 19.9 months, although the trial was not powered to show this. We conclude that, even with optimal dosing, vandetanib does not improve the progression-free or overall survival for AI-resistant patients over and above fulvestrant alone. Toxicity profiles were as expected for the two arms. It should be noted that the patient population treated in this study were naive to CDK4/6 inhibitors which are now used routinely in the first-line treatment of metastatic disease alongside aromatase inhibitors.

This study was also designed to examine clinical outcomes for FURVA in relation to tRET expression. Previous data has indicated that high tRET expression is associated with poor prognosis in ER-positive breast cancer [4], and also that tRET targeting can improve endocrine response in vitro, suggesting RET can drive the subsequent emergence of endocrine-resistant cells. Therefore the addition of vandetanib may be particularly beneficial in this ER+ve/RET+ve patient subgroup. In the subgroup analysis of FURVA participant outcomes by tRET expression, overall 42% of cases were classed as being high-RET expressors. However, a test for interaction between tRET expression and treatment group did not demonstrate that vandetanib conferred a greater advantage over fulvestrant alone in the high tRET expression subgroup than the overall intention-to-treat population. It may be that monitoring tRET expression in the archival sample is unable to reflect tRET signalling status once AI resistance develops (where biopsying was unfortunately not possible). We acknowledge the limitations of immunohistochemistry, and it may be that other methods, such as genomic analysis, might capture clinically more meaningful RET alterations. However, an interesting and unexpected finding in our analysis was that a high tRET score in the archival samples was associated with a longer PFS (8.87 months) compared to a low tRET score (3.94 months) regardless of treatment allocation: (HR 0.493: 95% CI 0.32–0.77; *P* = 0.002). This was further supported by a trend towards an OS advantage of 21.95 vs 18.04 (HR 0.584; 95% CI 0.34–1.00; *P* = 0.051).

The improved outcomes in the high tRET group is surprising given the trial hypothesised that high tRET expression might be associated with endocrine resistance and given that previous reports indicate high tRET is associated with worse prognosis in breast cancer [4]. In a post hoc analysis, we were unable to identify other features in the low tRET group that may have contributed to poor outcomes for this cohort. For example, patients with tumours expressing high tRET were not more heavily pre-treated in the metastatic setting, and this group was not enriched with patients who had measurable disease. A predictive effect of high tRET for fulvestrant benefit cannot be determined in this study. There might be several other reasons for this observation including differences in RET expression in the primary tumour and metastases, differences in detection of RET expression across the various antibodies employed or simply spurious results. It is however notable that the relationship between RET, ER and endocrine treatments remains complex and controversial. For example, while elevated tRET has been reported in some models that have developed resistance to prolonged endocrine treatment [5], tRET has also been shown in other studies to be an oestrogen/ER-regulated gene with ERE sequences in its promoter and so inhibited by endocrine treatment [24]. It is possible that a high tRET score predicts for benefit from fulvestrant over patients with tumours harbouring low tRET expression, although we cannot confidently conclude this is the case. Nevertheless, our findings tentatively imply that tRET testing might have a utility in clinical practice to determine whether patients should receive fulvestrant after prior endocrine therapy – the implication being that patients with a low tRET score might not be expected to have clinical benefit from fulvestrant and might be better served by palliative chemotherapy or other treatment options. The study was not initially designed with this in mind, and it would require further validation with a defined study involving patients who did or did not receive fulvestrant, but, if confirmed, IHC-monitored tRET expression might feasibly have potential as a laboratory biomarker of response to second-line fulvestrant therapy in the clinic.

## Supplementary information

FURVA Supplementary Material

## Data Availability

Qualified researchers may request access to de-identified individual participant data by completing the Sample or Sample and Data Application Form available on the CTR website (https://www.cardiff.ac.uk/centre-for-studies-research) and submitting a copy to CTR@cardiff.ac.uk which will be reviewed for scientific merit and feasibility in compliance with CTR policies/SOPs and regulatory requirements.
