# Supplementary material for: Fulvestrant plus vandetanib versus placebo for the treatment of patients with metastatic breast cancer resistant to aromatase inhibitor therapy (FURVA): a multicentre, Phase 2, randomised controlled trial
Source: BJC Rep. Author manuscript; Available in PMC 2025 Feb 24. (PMC11523984; doi:10.1038/s44276-023-00016-8)
Supplement: Supplemental material [file EMS193620-supplement-Supplemental_material_.pdf]

FURVA Supplementary Material

Figure S1. Progression-free survival by RET

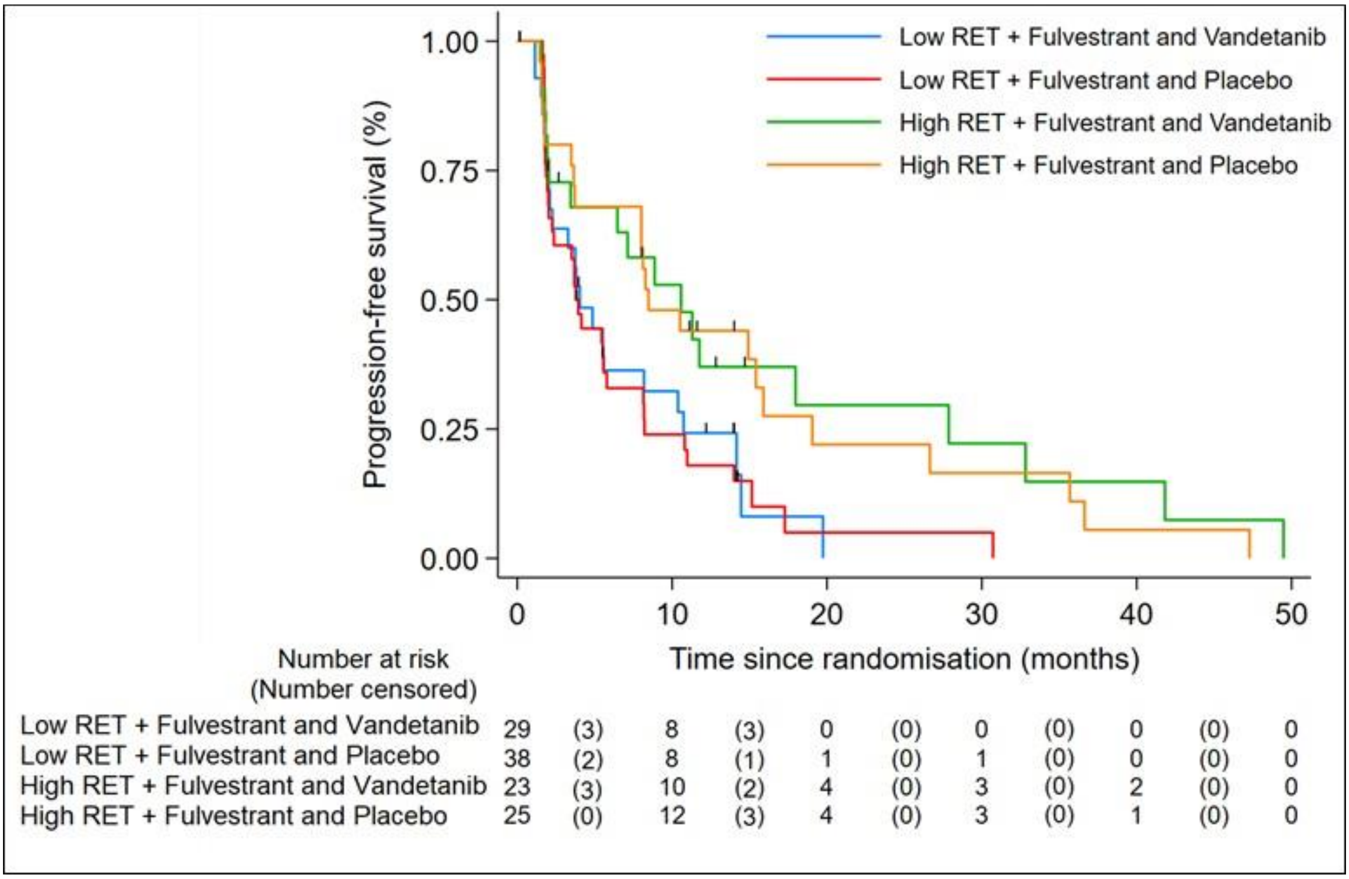

Kaplan-Meier curves were plotted to show the percentage of patients who were progression-free over time, by treatment group and RET group.

Table S1. Reported toxicities

	Fulvestrant plus vandetanib arm (n=80)					Fulvestrant plus Placebo arm (n=85)				
	Grade Reported N					Grade Reported N				
	1	2	3	4	5	1	2	3	4	5
**Total number of AEs**	741	283	58	3	2	581	165	34	7	0
**Patients with any AE (worst reported grade)**	8 (10.0%)	34 (42.5%)	34 (42.5%)	1 (1.3%)	2 (2.5%)	22 (25.9%)	38 (44.7%)	18 (21.2%)	6 (7.1%)	0 (0.0%)
**Patients with AE leading to death**	0 (0.0%)	0 (0.0%)	0 (0.0%)	0 (0.0%)	2 (2.5%)	0 (0.0%)	0 (0.0%)	0 (0.0%)	0 (0.0%)	0 (0.0%)
Haemorrhage intracranial	0 (0.0%)	0 (0.0%)	0 (0.0%)	0 (0.0%)	1 (1.3%)	0 (0.0%)	0 (0.0%)	0 (0.0%)	0 (0.0%)	0 (0.0%)
Pneumonia	0 (0.0%)	0 (0.0%)	0 (0.0%)	0 (0.0%)	1 (1.3%)	0 (0.0%)	0 (0.0%)	0 (0.0%)	0 (0.0%)	0 (0.0%)
**Patients with Serious AEs**										
Abdominal pain	0 (0%)	0 (0%)	1 (1.3%)	0 (0%)	0 (0%)	0 (0%)	0 (0%)	0 (0%)	0 (0%)	0 (0%)
Arthritis bacterial	0 (0%)	0 (0%)	1 (1.3%)	0 (0%)	0 (0%)	0 (0%)	0 (0%)	0 (0%)	0 (0%)	0 (0%)
Atrial fibrillation	0 (0%)	1 (1.3%)	0 (0%)	0 (0%)	0 (0%)	0 (0%)	0 (0%)	0 (0%)	0 (0%)	0 (0%)
Blindness	0 (0%)	0 (0%)	0 (0%)	0 (0%)	0 (0%)	0 (0%)	0 (0%)	1 (1.2%)	0 (0%)	0 (0%)
Bone pain	0 (0%)	0 (0%)	1 (1.3%)	0 (0%)	0 (0%)	0 (0%)	0 (0%)	0 (0%)	0 (0%)	0 (0%)
Cellulitis	0 (0%)	0 (0%)	1 (1.3%)	0 (0%)	0 (0%)	0 (0%)	0 (0%)	0 (0%)	0 (0%)	0 (0%)
Clavicle fracture	0 (0%)	0 (0%)	0 (0%)	0 (0%)	0 (0%)	0 (0%)	1 (1.2%)	0 (0%)	0 (0%)	0 (0%)
Colitis	0 (0%)	0 (0%)	1 (1.3%)	0 (0%)	0 (0%)	0 (0%)	0 (0%)	0 (0%)	0 (0%)	0 (0%)
Duodenal ulcer perforation	0 (0%)	0 (0%)	0 (0%)	0 (0%)	0 (0%)	0 (0%)	0 (0%)	0 (0%)	1 (1.2%)	0 (0%)
Dyspnoea	0 (0%)	0 (0%)	0 (0%)	0 (0%)	0 (0%)	0 (0%)	2 (2.4%)	0 (0%)	0 (0%)	0 (0%)
Haemorrhage intracranial	0 (0%)	0 (0%)	0 (0%)	0 (0%)	1 (1.3%)	0 (0%)	0 (0%)	0 (0%)	0 (0%)	0 (0%)
Headache	0 (0%)	0 (0%)	1 (1.3%)	0 (0%)	0 (0%)	0 (0%)	0 (0%)	0 (0%)	0 (0%)	0 (0%)
Hyponatraemia	0 (0%)	0 (0%)	0 (0%)	0 (0%)	0 (0%)	0 (0%)	0 (0%)	0 (0%)	1 (1.2%)	0 (0%)
Infection	0 (0%)	0 (0%)	1 (1.3%)	0 (0%)	0 (0%)	0 (0%)	0 (0%)	0 (0%)	0 (0%)	0 (0%)
Lung infection	0 (0%)	0 (0%)	1 (1.3%)	0 (0%)	0 (0%)	0 (0%)	0 (0%)	0 (0%)	0 (0%)	0 (0%)
Malaise	0 (0%)	0 (0%)	0 (0%)	0 (0%)	0 (0%)	0 (0%)	1 (1.2%)	0 (0%)	0 (0%)	0 (0%)
Muscular weakness	0 (0%)	0 (0%)	1 (1.3%)	0 (0%)	0 (0%)	0 (0%)	0 (0%)	0 (0%)	0 (0%)	0 (0%)
Nausea	0 (0%)	0 (0%)	0 (0%)	0 (0%)	0 (0%)	0 (0%)	1 (1.2%)	0 (0%)	0 (0%)	0 (0%)
Pleural effusion	0 (0%)	0 (0%)	1 (1.3%)	0 (0%)	0 (0%)	0 (0%)	1 (1.2%)	1 (1.2%)	0 (0%)	0 (0%)
Pneumonia	0 (0%)	0 (0%)	0 (0%)	0 (0%)	1 (1.3%)	0 (0%)	0 (0%)	0 (0%)	0 (0%)	0 (0%)
Rectal haemorrhage	0 (0%)	0 (0%)	1 (1.3%)	0 (0%)	0 (0%)	0 (0%)	0 (0%)	0 (0%)	0 (0%)	0 (0%)
Small intestinal obstruction	0 (0%)	0 (0%)	0 (0%)	0 (0%)	0 (0%)	0 (0%)	0 (0%)	1 (1.2%)	0 (0%)	0 (0%)
Stomatitis	0 (0%)	0 (0%)	0 (0%)	0 (0%)	0 (0%)	0 (0%)	0 (0%)	1 (1.2%)	0 (0%)	0 (0%)

Urinary tract infection	0 (0%)	0 (0%)	1 (1.3%)	0 (0%)	0 (0%)	0 (0%)	0 (0%)	0 (0%)	0 (0%)	0 (0%)
Viral infection	0 (0%)	0 (0%)	0 (0%)	0 (0%)	0 (0%)	0 (0%)	0 (0%)	1 (1.2%)	0 (0%)	0 (0%)
**Serious Adverse Reactions**										
Confusional state	0 (0%)	1 (1.3%)	0 (0%)	0 (0%)	0 (0%)	0 (0%)	0 (0%)	0 (0%)	0 (0%)	0 (0%)
Dehydration	0 (0%)	0 (0%)	1 (1.3%)	0 (0%)	0 (0%)	0 (0%)	0 (0%)	0 (0%)	0 (0%)	0 (0%)
Diarrhoea	0 (0%)	0 (0%)	2 (2.5%)	0 (0%)	0 (0%)	0 (0%)	0 (0%)	0 (0%)	0 (0%)	0 (0%)
Infection	0 (0%)	0 (0%)	1 (1.3%)	0 (0%)	0 (0%)	0 (0%)	0 (0%)	0 (0%)	0 (0%)	0 (0%)
Left ventricular dysfunction	0 (0%)	0 (0%)	1 (1.3%)	0 (0%)	0 (0%)	0 (0%)	0 (0%)	0 (0%)	0 (0%)	0 (0%)
Photosensitivity reaction	1 (1.3%)	0 (0%)	1 (1.3%)	0 (0%)	0 (0%)	0 (0%)	0 (0%)	0 (0%)	0 (0%)	0 (0%)
Pulmonary embolism	0 (0%)	0 (0%)	0 (0%)	0 (0%)	0 (0%)	0 (0%)	0 (0%)	1 (1.2%)	0 (0%)	0 (0%)
Seizure	0 (0%)	1 (1.3%)	0 (0%)	0 (0%)	0 (0%)	0 (0%)	1 (1.2%)	0 (0%)	0 (0%)	0 (0%)
Toxic epidermal necrolysis	0 (0%)	0 (0%)	0 (0%)	1 (1.3%)	0 (0%)	0 (0%)	0 (0%)	0 (0%)	0 (0%)	0 (0%)
**Adverse events**										
Blood and lymphatic system disorders										
anaemia	3 (3.8%)	0 (0%)	2 (2.5%)	0 (0%)	0 (0%)	3 (3.5%)	2 (2.4%)	2 (2.4%)	0 (0%)	0 (0%)
thrombocytopenia	0 (0%)	0 (0%)	0 (0%)	0 (0%)	0 (0%)	0 (0%)	1 (1.2%)	0 (0%)	0 (0%)	0 (0%)
Cardiac disorders										
arrythmia	2 (2.5%)	3 (3.8%)	1 (1.3%)	0 (0%)	0 (0%)	3 (3.5%)	3 (3.5%)	0 (0%)	0 (0%)	0 (0%)
atrial fibrillation	0 (0%)	1 (1.3%)	0 (0%)	0 (0%)	0 (0%)	0 (0%)	0 (0%)	0 (0%)	0 (0%)	0 (0%)
bundle branch block left	0 (0%)	0 (0%)	0 (0%)	0 (0%)	0 (0%)	1 (1.2%)	0 (0%)	0 (0%)	0 (0%)	0 (0%)
chest pain	1 (1.3%)	0 (0%)	0 (0%)	0 (0%)	0 (0%)	1 (1.2%)	0 (0%)	0 (0%)	0 (0%)	0 (0%)
electrocardiogram abnormal	1 (1.3%)	0 (0%)	0 (0%)	0 (0%)	0 (0%)	0 (0%)	0 (0%)	0 (0%)	0 (0%)	0 (0%)
electrocardiogram qt interval abnormal	0 (0%)	1 (1.3%)	0 (0%)	0 (0%)	0 (0%)	0 (0%)	0 (0%)	0 (0%)	0 (0%)	0 (0%)
electrocardiogram qt prolonged	2 (2.5%)	1 (1.3%)	0 (0%)	0 (0%)	0 (0%)	0 (0%)	0 (0%)	1 (1.2%)	0 (0%)	0 (0%)
left ventricular dysfunction	0 (0%)	0 (0%)	1 (1.3%)	0 (0%)	0 (0%)	0 (0%)	0 (0%)	0 (0%)	0 (0%)	0 (0%)
left ventricular systolic dysfunction	1 (1.3%)	2 (2.5%)	0 (0%)	0 (0%)	0 (0%)	0 (0%)	0 (0%)	0 (0%)	0 (0%)	0 (0%)
palpitations	6 (7.5%)	0 (0%)	0 (0%)	0 (0%)	0 (0%)	0 (0%)	0 (0%)	0 (0%)	0 (0%)	0 (0%)
sinus tachycardia	1 (1.3%)	0 (0%)	0 (0%)	0 (0%)	0 (0%)	1 (1.2%)	0 (0%)	0 (0%)	0 (0%)	0 (0%)
tachycardia	0 (0%)	0 (0%)	0 (0%)	0 (0%)	0 (0%)	0 (0%)	1 (1.2%)	0 (0%)	0 (0%)	0 (0%)
ventricular extrasystoles	0 (0%)	1 (1.3%)	0 (0%)	0 (0%)	0 (0%)	0 (0%)	0 (0%)	0 (0%)	0 (0%)	0 (0%)
Ear and labyrinth disorders										
deafness	1 (1.3%)	0 (0%)	0 (0%)	0 (0%)	0 (0%)	0 (0%)	0 (0%)	0 (0%)	0 (0%)	0 (0%)
Eye disorders										
amblyopia	1 (1.3%)	0 (0%)	0 (0%)	0 (0%)	0 (0%)	0 (0%)	0 (0%)	0 (0%)	0 (0%)	0 (0%)
blindness	0 (0%)	0 (0%)	0 (0%)	0 (0%)	0 (0%)	0 (0%)	0 (0%)	1 (1.2%)	0 (0%)	0 (0%)

conjunctivititis viral	1 (1.3%)	0 (0%)	0 (0%)	0 (0%)	0 (0%)	0 (0%)	0 (0%)	0 (0%)	0 (0%)	0 (0%)
diplopia	0 (0%)	1 (1.3%)	0 (0%)	0 (0%)	0 (0%)	0 (0%)	0 (0%)	0 (0%)	0 (0%)	0 (0%)
dry eye	1 (1.3%)	1 (1.3%)	0 (0%)	0 (0%)	0 (0%)	3 (3.5%)	0 (0%)	0 (0%)	0 (0%)	0 (0%)
eye pain	1 (1.3%)	0 (0%)	0 (0%)	0 (0%)	0 (0%)	0 (0%)	0 (0%)	0 (0%)	0 (0%)	0 (0%)
eyelid ptosis	0 (0%)	0 (0%)	0 (0%)	0 (0%)	0 (0%)	1 (1.2%)	0 (0%)	0 (0%)	0 (0%)	0 (0%)
haemorrhage	0 (0%)	0 (0%)	0 (0%)	0 (0%)	0 (0%)	1 (1.2%)	0 (0%)	0 (0%)	0 (0%)	0 (0%)
miosis	0 (0%)	0 (0%)	0 (0%)	0 (0%)	0 (0%)	1 (1.2%)	0 (0%)	0 (0%)	0 (0%)	0 (0%)
photopsia	1 (1.3%)	0 (0%)	0 (0%)	0 (0%)	0 (0%)	0 (0%)	0 (0%)	0 (0%)	0 (0%)	0 (0%)
photosensitivity reaction	0 (0%)	0 (0%)	0 (0%)	0 (0%)	0 (0%)	1 (1.2%)	0 (0%)	0 (0%)	0 (0%)	0 (0%)
pruritus	1 (1.3%)	0 (0%)	0 (0%)	0 (0%)	0 (0%)	0 (0%)	0 (0%)	0 (0%)	0 (0%)	0 (0%)
vision blurred	1 (1.3%)	2 (2.5%)	0 (0%)	0 (0%)	0 (0%)	1 (1.2%)	2 (2.4%)	0 (0%)	0 (0%)	0 (0%)
visual impairment	2 (2.5%)	0 (0%)	0 (0%)	0 (0%)	0 (0%)	0 (0%)	0 (0%)	0 (0%)	0 (0%)	0 (0%)
Gastrointestinal disorders										
abdominal distension	2 (2.5%)	1 (1.3%)	0 (0%)	0 (0%)	0 (0%)	3 (3.5%)	0 (0%)	0 (0%)	0 (0%)	0 (0%)
abdominal pain	8 (10%)	1 (1.3%)	1 (1.3%)	0 (0%)	0 (0%)	6 (7.1%)	1 (1.2%)	1 (1.2%)	0 (0%)	0 (0%)
abdominal tenderness	0 (0%)	0 (0%)	0 (0%)	0 (0%)	0 (0%)	1 (1.2%)	0 (0%)	0 (0%)	0 (0%)	0 (0%)
ascites	0 (0%)	0 (0%)	0 (0%)	0 (0%)	0 (0%)	0 (0%)	0 (0%)	1 (1.2%)	0 (0%)	0 (0%)
colitis	0 (0%)	0 (0%)	1 (1.3%)	0 (0%)	0 (0%)	0 (0%)	0 (0%)	0 (0%)	0 (0%)	0 (0%)
constipation	13 (16.3%)	6 (7.5%)	0 (0%)	0 (0%)	0 (0%)	12 (14.1%)	4 (4.7%)	0 (0%)	0 (0%)	0 (0%)
decreased appetite	0 (0%)	0 (0%)	0 (0%)	0 (0%)	0 (0%)	2 (2.4%)	0 (0%)	0 (0%)	0 (0%)	0 (0%)
diarrhoea	32 (40.0%)	15 (18.8%)	9 (11.3%)	0 (0%)	0 (0%)	22 (25.9%)	2 (2.4%)	1 (1.2%)	0 (0%)	0 (0%)
diverticulitis	0 (0%)	0 (0%)	0 (0%)	0 (0%)	0 (0%)	0 (0%)	1 (1.2%)	0 (0%)	0 (0%)	0 (0%)
dry mouth	6 (7.5%)	1 (1.25%)	0 (0%)	0 (0%)	0 (0%)	4 (4.7%)	0 (0%)	0 (0%)	0 (0%)	0 (0%)
duodenal ulcer perforation	0 (0%)	0 (0%)	0 (0%)	0 (0%)	0 (0%)	0 (0%)	0 (0%)	0 (0%)	1 (1.2%)	0 (0%)
duodenogastric reflux	0 (0%)	0 (0%)	0 (0%)	0 (0%)	0 (0%)	1 (1.2%)	0 (0%)	0 (0%)	0 (0%)	0 (0%)
dysgeusia	3 (3.8%)	0 (0%)	0 (0%)	0 (0%)	0 (0%)	2 (2.4%)	0 (0%)	0 (0%)	0 (0%)	0 (0%)
dyspepsia	9 (11.3%)	3 (3.8%)	0 (0%)	0 (0%)	0 (0%)	0 (0%)	1 (1.2%)	0 (0%)	0 (0%)	0 (0%)
dysphagia	0 (0%)	0 (0%)	0 (0%)	0 (0%)	0 (0%)	1 (1.2%)	0 (0%)	0 (0%)	0 (0%)	0 (0%)
dysplasia	1 (1.3%)	0 (0%)	0 (0%)	0 (0%)	0 (0%)	0 (0%)	0 (0%)	0 (0%)	0 (0%)	0 (0%)
early satiety	1 (1.3%)	0 (0%)	0 (0%)	0 (0%)	0 (0%)	0 (0%)	0 (0%)	0 (0%)	0 (0%)	0 (0%)
flatulence	3 (3.8%)	0 (0%)	0 (0%)	0 (0%)	0 (0%)	0 (0%)	0 (0%)	0 (0%)	0 (0%)	0 (0%)
frequent bowel movements	1 (1.3%)	0 (0%)	0 (0%)	0 (0%)	0 (0%)	0 (0%)	0 (0%)	0 (0%)	0 (0%)	0 (0%)
gingival bleeding	2 (2.5%)	0 (0%)	0 (0%)	0 (0%)	0 (0%)	0 (0%)	0 (0%)	0 (0%)	0 (0%)	0 (0%)
gingival blister	0 (0%)	0 (0%)	0 (0%)	0 (0%)	0 (0%)	0 (0%)	1 (1.2%)	0 (0%)	0 (0%)	0 (0%)
gingival infection	0 (0%)	1 (1.3%)	0 (0%)	0 (0%)	0 (0%)	0 (0%)	0 (0%)	0 (0%)	0 (0%)	0 (0%)
gingival pain	2 (2.5%)	0 (0%)	0 (0%)	0 (0%)	0 (0%)	0 (0%)	0 (0%)	0 (0%)	0 (0%)	0 (0%)
haematemesis	0 (0%)	0 (0%)	0 (0%)	0 (0%)	0 (0%)	0 (0%)	0 (0%)	0 (0%)	0 (0%)	0 (0%)

haemorrhoids	0 (0%)	0 (0%)	0 (0%)	0 (0%)	0 (0%)	1 (1.2%)	0 (0%)	0 (0%)	0 (0%)	0 (0%)
mouth ulceration	3 (3.8%)	0 (0%)	0 (0%)	0 (0%)	0 (0%)	2 (2.4%)	0 (0%)	0 (0%)	0 (0%)	0 (0%)
mucosal inflammation	9 (11.3%)	5 (6.3%)	0 (0%)	0 (0%)	0 (0%)	8 (9.4%)	0 (0%)	0 (0%)	0 (0%)	0 (0%)
nausea	33 (41.3%)	11 (13.8%)	1 (1.3%)	0 (0%)	0 (0%)	31 (36.5%)	7 (8.2%)	1 (1.2%)	0 (0%)	0 (0%)
odynophagia	1 (1.3%)	0 (0%)	0 (0%)	0 (0%)	0 (0%)	0 (0%)	0 (0%)	0 (0%)	0 (0%)	0 (0%)
oral candidiasis	1 (1.3%)	1 (1.3%)	0 (0%)	0 (0%)	0 (0%)	1 (1.2%)	0 (0%)	0 (0%)	0 (0%)	0 (0%)
oral pain	1 (1.3%)	0 (0%)	0 (0%)	0 (0%)	0 (0%)	2 (2.4%)	0 (0%)	0 (0%)	0 (0%)	0 (0%)
oropharyngeal pain	0 (0%)	0 (0%)	0 (0%)	0 (0%)	0 (0%)	1 (1.2%)	0 (0%)	0 (0%)	0 (0%)	0 (0%)
rectal haemorrhage	0 (0%)	0 (0%)	1 (1.3%)	0 (0%)	0 (0%)	1 (1.2%)	0 (0%)	0 (0%)	0 (0%)	0 (0%)
retching	1 (1.3%)	0 (0%)	0 (0%)	0 (0%)	0 (0%)	0 (0%)	0 (0%)	0 (0%)	0 (0%)	0 (0%)
small intestinal obstruction	0 (0%)	0 (0%)	0 (0%)	0 (0%)	0 (0%)	0 (0%)	0 (0%)	1 (1.2%)	0 (0%)	0 (0%)
stomatitis	3 (3.8%)	0 (0%)	0 (0%)	0 (0%)	0 (0%)	3 (3.5%)	0 (0%)	1 (1.2%)	0 (0%)	0 (0%)
tongue coated	1 (1.3%)	0 (0%)	0 (0%)	0 (0%)	0 (0%)	0 (0%)	0 (0%)	0 (0%)	0 (0%)	0 (0%)
tooth injury	1 (1.3%)	0 (0%)	0 (0%)	0 (0%)	0 (0%)	0 (0%)	0 (0%)	0 (0%)	0 (0%)	0 (0%)
toothache	1 (1.3%)	1 (1.3%)	0 (0%)	0 (0%)	0 (0%)	0 (0%)	0 (0%)	0 (0%)	0 (0%)	0 (0%)
vomiting	20 (25.0%)	4 (5.0%)	0 (0%)	0 (0%)	0 (0%)	12 (14.1%)	2 (2.4%)	0 (0%)	0 (0%)	0 (0%)
General disorders and administration site conditions										
axillary pain	2 (2.5%)	0 (0%)	0 (0%)	0 (0%)	0 (0%)	0 (0%)	0 (0%)	0 (0%)	0 (0%)	0 (0%)
back pain	1 (1.3%)	1 (1.3%)	0 (0%)	0 (0%)	0 (0%)	0 (0%)	0 (0%)	0 (0%)	0 (0%)	0 (0%)
body temperature increased	1 (1.3%)	0 (0%)	0 (0%)	0 (0%)	0 (0%)	0 (0%)	0 (0%)	0 (0%)	0 (0%)	0 (0%)
chest pain	0 (0%)	1 (1.3%)	0 (0%)	0 (0%)	0 (0%)	1 (1.2%)	0 (0%)	0 (0%)	0 (0%)	0 (0%)
chills	0 (0%)	1 (1.3%)	0 (0%)	0 (0%)	0 (0%)	0 (0%)	0 (0%)	0 (0%)	0 (0%)	0 (0%)
discomfort	1 (1.3%)	0 (0%)	0 (0%)	0 (0%)	0 (0%)	0 (0%)	0 (0%)	0 (0%)	0 (0%)	0 (0%)
facial pain	0 (0%)	0 (0%)	0 (0%)	0 (0%)	0 (0%)	1 (1.2%)	0 (0%)	0 (0%)	0 (0%)	0 (0%)
fatigue	23 (28.8%)	34 (42.5%)	4 (5%)	0 (0%)	0 (0%)	37 (43.5%)	16 (18.8%)	3 (3.5%)	0 (0%)	0 (0%)
gait disturbance	1 (1.3%)	0 (0%)	0 (0%)	0 (0%)	0 (0%)	0 (0%)	0 (0%)	0 (0%)	0 (0%)	0 (0%)
influenza like illness	2 (2.5%)	1 (1.3%)	0 (0%)	0 (0%)	0 (0%)	1 (1.2%)	0 (0%)	0 (0%)	0 (0%)	0 (0%)
injection site discomfort	0 (0%)	0 (0%)	0 (0%)	0 (0%)	0 (0%)	2 (2.4%)	0 (0%)	0 (0%)	0 (0%)	0 (0%)
injection site pain	2 (2.5%)	0 (0%)	0 (0%)	0 (0%)	0 (0%)	0 (0%)	1 (1.2%)	0 (0%)	0 (0%)	0 (0%)
injection site reactions	12 (15.0%)	2 (2.5%)	0 (0%)	0 (0%)	0 (0%)	12 (14.1%)	2 (2.4%)	0 (0%)	0 (0%)	0 (0%)
injection site swelling	1 (1.3%)	0 (0%)	0 (0%)	0 (0%)	0 (0%)	0 (0%)	0 (0%)	0 (0%)	0 (0%)	0 (0%)
lethargy	0 (0%)	0 (0%)	0 (0%)	0 (0%)	0 (0%)	1 (1.2%)	0 (0%)	0 (0%)	0 (0%)	0 (0%)
lymphoedema	0 (0%)	0 (0%)	0 (0%)	0 (0%)	0 (0%)	1 (1.2%)	0 (0%)	0 (0%)	0 (0%)	0 (0%)
malaise	0 (0%)	0 (0%)	0 (0%)	0 (0%)	0 (0%)	0 (0%)	1 (1.2%)	0 (0%)	0 (0%)	0 (0%)
mouth ulceration	1 (1.3%)	0 (0%)	0 (0%)	0 (0%)	0 (0%)	0 (0%)	0 (0%)	0 (0%)	0 (0%)	0 (0%)
muscoskeletal pain	1 (1.3%)	0 (0%)	0 (0%)	0 (0%)	0 (0%)	1 (1.2%)	1 (1.2%)	0 (0%)	0 (0%)	0 (0%)
musculoskeletal chest pain	1 (1.3%)	1 (1.3%)	0 (0%)	0 (0%)	0 (0%)	0 (0%)	0 (0%)	0 (0%)	0 (0%)	0 (0%)

musculoskeletal pain	1 (1.3%)	0 (0%)	0 (0%)	0 (0%)	0 (0%)	0 (0%)	0 (0%)	0 (0%)	0 (0%)	0 (0%)
nasal discomfort	0 (0%)	1 (1.3%)	0 (0%)	0 (0%)	0 (0%)	0 (0%)	0 (0%)	0 (0%)	0 (0%)	0 (0%)
nasopharyngitis	2 (2.5%)	0 (0%)	0 (0%)	0 (0%)	0 (0%)	3 (3.5%)	0 (0%)	0 (0%)	0 (0%)	0 (0%)
night sweats	0 (0%)	2 (2.5%)	0 (0%)	0 (0%)	0 (0%)	0 (0%)	0 (0%)	0 (0%)	0 (0%)	0 (0%)
non-cardiac chest pain	0 (0%)	0 (0%)	0 (0%)	0 (0%)	0 (0%)	1 (1.2%)	0 (0%)	0 (0%)	0 (0%)	0 (0%)
odema peripheral	0 (0%)	1 (1.3%)	0 (0%)	0 (0%)	0 (0%)	1 (1.2%)	0 (0%)	0 (0%)	0 (0%)	0 (0%)
oedema peripheral	1 (1.3%)	1 (1.3%)	0 (0%)	0 (0%)	0 (0%)	1 (1.2%)	0 (0%)	0 (0%)	0 (0%)	0 (0%)
oral pain	0 (0%)	1 (1.3%)	0 (0%)	0 (0%)	0 (0%)	0 (0%)	0 (0%)	0 (0%)	0 (0%)	0 (0%)
pain	1 (1.3%)	2 (2.5%)	0 (0%)	0 (0%)	0 (0%)	1 (1.2%)	0 (0%)	0 (0%)	0 (0%)	0 (0%)
pain in extremity	0 (0%)	1 (1.3%)	0 (0%)	0 (0%)	0 (0%)	1 (1.2%)	0 (0%)	0 (0%)	0 (0%)	0 (0%)
pleuritic pain	0 (0%)	0 (0%)	0 (0%)	0 (0%)	0 (0%)	1 (1.2%)	0 (0%)	0 (0%)	0 (0%)	0 (0%)
pruritus	1 (1.3%)	0 (0%)	0 (0%)	0 (0%)	0 (0%)	0 (0%)	0 (0%)	0 (0%)	0 (0%)	0 (0%)
pyrexia	3 (3.8%)	0 (0%)	0 (0%)	0 (0%)	0 (0%)	0 (0%)	0 (0%)	0 (0%)	0 (0%)	0 (0%)
swelling face	0 (0%)	0 (0%)	0 (0%)	0 (0%)	0 (0%)	1 (1.2%)	0 (0%)	0 (0%)	0 (0%)	0 (0%)
Hepatobiliary disorders										
cholecystitis	0 (0%)	0 (0%)	0 (0%)	0 (0%)	0 (0%)	0 (0%)	1 (1.2%)	0 (0%)	0 (0%)	0 (0%)
gamma-glutamyltransferase increased	1 (1.3%)	0 (0%)	0 (0%)	0 (0%)	0 (0%)	0 (0%)	0 (0%)	0 (0%)	1 (1.2%)	0 (0%)
Infections and infestations										
arthiritis bacterial	0 (0%)	0 (0%)	1 (1.3%)	0 (0%)	0 (0%)	0 (0%)	0 (0%)	0 (0%)	0 (0%)	0 (0%)
biliary sepsis	0 (0%)	0 (0%)	1 (1.3%)	0 (0%)	0 (0%)	0 (0%)	0 (0%)	0 (0%)	0 (0%)	0 (0%)
cellulitis	0 (0%)	0 (0%)	1 (1.3%)	0 (0%)	0 (0%)	0 (0%)	1 (1.2%)	0 (0%)	0 (0%)	0 (0%)
foreign body trauma	2 (2.5%)	0 (0%)	0 (0%)	0 (0%)	0 (0%)	0 (0%)	0 (0%)	0 (0%)	0 (0%)	0 (0%)
infection	1 (1.3%)	0 (0%)	3 (3.8%)	0 (0%)	0 (0%)	0 (0%)	0 (0%)	0 (0%)	0 (0%)	0 (0%)
kidney infection	0 (0%)	0 (0%)	0 (0%)	0 (0%)	0 (0%)	0 (0%)	1 (1.2%)	0 (0%)	0 (0%)	0 (0%)
localised infection	0 (0%)	0 (0%)	0 (0%)	0 (0%)	0 (0%)	1 (1.2%)	0 (0%)	0 (0%)	0 (0%)	0 (0%)
lower respiratory tract infection	0 (0%)	6 (7.5%)	0 (0%)	0 (0%)	0 (0%)	0 (0%)	4 (4.7%)	1 (1.2%)	0 (0%)	0 (0%)
lung infection	0 (0%)	0 (0%)	1 (1.3%)	0 (0%)	0 (0%)	0 (0%)	2 (2.4%)	0 (0%)	0 (0%)	0 (0%)
mouth ulceration	0 (0%)	0 (0%)	0 (0%)	0 (0%)	0 (0%)	1 (1.2%)	0 (0%)	0 (0%)	0 (0%)	0 (0%)
mucosal inflammation	0 (0%)	1 (1.3%)	0 (0%)	0 (0%)	0 (0%)	0 (0%)	0 (0%)	0 (0%)	0 (0%)	0 (0%)
nail infection	0 (0%)	1 (1.3%)	0 (0%)	0 (0%)	0 (0%)	0 (0%)	1 (1.2%)	0 (0%)	0 (0%)	0 (0%)
nasopharyngitis	0 (0%)	0 (0%)	0 (0%)	0 (0%)	0 (0%)	1 (1.2%)	0 (0%)	0 (0%)	0 (0%)	0 (0%)
oral candidiasis	1 (1.3%)	0 (0%)	0 (0%)	0 (0%)	0 (0%)	0 (0%)	0 (0%)	0 (0%)	0 (0%)	0 (0%)
oral herpes	1 (1.3%)	0 (0%)	0 (0%)	0 (0%)	0 (0%)	0 (0%)	0 (0%)	0 (0%)	0 (0%)	0 (0%)
osteomyelitis	0 (0%)	0 (0%)	0 (0%)	0 (0%)	0 (0%)	0 (0%)	1 (1.2%)	0 (0%)	0 (0%)	0 (0%)
pharyngitis	1 (1.3%)	0 (0%)	0 (0%)	0 (0%)	0 (0%)	0 (0%)	0 (0%)	0 (0%)	0 (0%)	0 (0%)
pneumonia	0 (0%)	0 (0%)	0 (0%)	0 (0%)	1 (1.3%)	0 (0%)	0 (0%)	1 (1.2%)	0 (0%)	0 (0%)
pyrexia	0 (0%)	0 (0%)	0 (0%)	0 (0%)	0 (0%)	1 (1.2%)	0 (0%)	0 (0%)	0 (0%)	0 (0%)

rhinorrhea	1 (1.3%)	0 (0%)	0 (0%)	0 (0%)	0 (0%)	0 (0%)	0 (0%)	0 (0%)	0 (0%)	0 (0%)
sepsis	0 (0%)	0 (0%)	1 (1.3%)	0 (0%)	0 (0%)	0 (0%)	0 (0%)	0 (0%)	0 (0%)	0 (0%)
sinusitis	0 (0%)	0 (0%)	0 (0%)	0 (0%)	0 (0%)	1 (1.2%)	0 (0%)	0 (0%)	0 (0%)	0 (0%)
skin infection	0 (0%)	0 (0%)	0 (0%)	0 (0%)	0 (0%)	1 (1.2%)	0 (0%)	0 (0%)	0 (0%)	0 (0%)
tinae pedis	0 (0%)	0 (0%)	0 (0%)	0 (0%)	0 (0%)	1 (1.2%)	0 (0%)	0 (0%)	0 (0%)	0 (0%)
tonsillitis	0 (0%)	1 (1.3%)	0 (0%)	0 (0%)	0 (0%)	1 (1.2%)	0 (0%)	0 (0%)	0 (0%)	0 (0%)
upper respiratory tract infection	2 (2.5%)	0 (0%)	0 (0%)	0 (0%)	0 (0%)	1 (1.2%)	1 (1.2%)	0 (0%)	0 (0%)	0 (0%)
urinary tract infection	0 (0%)	5 (6.3%)	2 (2.5%)	0 (0%)	0 (0%)	2 (2.4%)	2 (2.4%)	0 (0%)	0 (0%)	0 (0%)
urosepsis	0 (0%)	0 (0%)	0 (0%)	0 (0%)	0 (0%)	0 (0%)	0 (0%)	1 (1.2%)	0 (0%)	0 (0%)
viral infection	0 (0%)	0 (0%)	0 (0%)	0 (0%)	0 (0%)	0 (0%)	0 (0%)	1 (1.2%)	0 (0%)	0 (0%)
vulvivaginal candidiasis	0 (0%)	0 (0%)	0 (0%)	0 (0%)	0 (0%)	0 (0%)	1 (1.2%)	0 (0%)	0 (0%)	0 (0%)
wound infection	0 (0%)	0 (0%)	0 (0%)	0 (0%)	0 (0%)	0 (0%)	1 (1.2%)	0 (0%)	0 (0%)	0 (0%)
Injury, poisoning and procedural complications										
clavicle fracture	0 (0%)	0 (0%)	0 (0%)	0 (0%)	0 (0%)	0 (0%)	1 (1.2%)	0 (0%)	0 (0%)	0 (0%)
contusion	1 (1.3%)	0 (0%)	0 (0%)	0 (0%)	0 (0%)	2 (2.4%)	0 (0%)	0 (0%)	0 (0%)	0 (0%)
fall	0 (0%)	0 (0%)	0 (0%)	0 (0%)	0 (0%)	1 (1.2%)	0 (0%)	0 (0%)	0 (0%)	0 (0%)
fracture	0 (0%)	0 (0%)	0 (0%)	0 (0%)	0 (0%)	0 (0%)	1 (1.2%)	0 (0%)	0 (0%)	0 (0%)
Investigations										
blood alanine transaminase increased	32 (40.0%)	8 (10%)	0 (0%)	0 (0%)	0 (0%)	21 (24.7%)	3 (3.5%)	1 (1.2%)	1 (1.2%)	0 (0%)
blood albumin increased	1 (1.3%)	0 (0%)	0 (0%)	0 (0%)	0 (0%)	0 (0%)	0 (0%)	0 (0%)	0 (0%)	0 (0%)
blood alkaline phosphatase increased	26 (32.5%)	7 (8.8%)	1 (1.3%)	0 (0%)	0 (0%)	23 (27.1%)	6 (7.1%)	0 (0%)	1 (1.2%)	0 (0%)
blood aspartate aminotransferase increased	8 (10%)	2 (2.5%)	1 (1.3%)	0 (0%)	0 (0%)	6 (7.1%)	0 (0%)	0 (0%)	0 (0%)	0 (0%)
blood bilirubin increased	7 (8.8%)	3 (3.8%)	2 (2.5%)	0 (0%)	0 (0%)	2 (2.4%)	1 (1.2%)	2 (2.4%)	0 (0%)	0 (0%)
blood creatinine	2 (2.5%)	0 (0%)	0 (0%)	0 (0%)	0 (0%)	0 (0%)	0 (0%)	0 (0%)	0 (0%)	0 (0%)
blood creatinine increased	7 (8.8%)	0 (0%)	0 (0%)	0 (0%)	0 (0%)	1 (1.2%)	0 (0%)	0 (0%)	0 (0%)	0 (0%)
blood sodium decreased	0 (0%)	0 (0%)	0 (0%)	0 (0%)	0 (0%)	1 (1.2%)	0 (0%)	0 (0%)	0 (0%)	0 (0%)
blood urea increased	1 (1.3%)	0 (0%)	0 (0%)	0 (0%)	0 (0%)	1 (1.2%)	0 (0%)	0 (0%)	0 (0%)	0 (0%)
carbohydrate antigen 15-3 increased	0 (0%)	0 (0%)	0 (0%)	0 (0%)	0 (0%)	1 (1.2%)	0 (0%)	0 (0%)	0 (0%)	0 (0%)
electrocardiogram qt prolonged	5 (6.3%)	3 (3.8%)	0 (0%)	0 (0%)	0 (0%)	0 (0%)	0 (0%)	0 (0%)	0 (0%)	0 (0%)
haemoglobin increased	1 (1.3%)	0 (0%)	0 (0%)	0 (0%)	0 (0%)	1 (1.2%)	0 (0%)	0 (0%)	0 (0%)	0 (0%)
international normalised ratio increased	0 (0%)	0 (0%)	0 (0%)	0 (0%)	0 (0%)	1 (1.2%)	0 (0%)	0 (0%)	0 (0%)	0 (0%)
lymphocyte count decreased	0 (0%)	0 (0%)	0 (0%)	0 (0%)	0 (0%)	1 (1.2%)	0 (0%)	0 (0%)	0 (0%)	0 (0%)
monocyte count increased	0 (0%)	0 (0%)	0 (0%)	0 (0%)	0 (0%)	1 (1.2%)	0 (0%)	0 (0%)	0 (0%)	0 (0%)

neutrophil count decreased	1 (1.3%)	0 (0%)	0 (0%)	0 (0%)	0 (0%)	0 (0%)	0 (0%)	0 (0%)	0 (0%)	0 (0%)
neutrophil count increased	1 (1.3%)	0 (0%)	0 (0%)	0 (0%)	0 (0%)	0 (0%)	0 (0%)	0 (0%)	0 (0%)	0 (0%)
platelet count decreased	1 (1.3%)	1 (1.3%)	0 (0%)	0 (0%)	0 (0%)	0 (0%)	0 (0%)	0 (0%)	0 (0%)	0 (0%)
thrombocytosis	1 (1.3%)	0 (0%)	0 (0%)	0 (0%)	0 (0%)	0 (0%)	0 (0%)	0 (0%)	0 (0%)	0 (0%)
white blood cell count decreased	0 (0%)	0 (0%)	0 (0%)	0 (0%)	0 (0%)	0 (0%)	1 (1.2%)	0 (0%)	0 (0%)	0 (0%)
white blood cell count increased	1 (1.3%)	0 (0%)	0 (0%)	0 (0%)	0 (0%)	1 (1.2%)	0 (0%)	0 (0%)	0 (0%)	0 (0%)
Metabolism and nutrition disorders										
blood glucose increased	0 (0%)	0 (0%)	0 (0%)	0 (0%)	0 (0%)	0 (0%)	0 (0%)	0 (0%)	1 (1.2%)	0 (0%)
blood magnesium increased	1 (1.3%)	0 (0%)	0 (0%)	0 (0%)	0 (0%)	0 (0%)	0 (0%)	0 (0%)	0 (0%)	0 (0%)
blood potassium increased	0 (0%)	0 (0%)	0 (0%)	0 (0%)	0 (0%)	1 (1.2%)	0 (0%)	0 (0%)	0 (0%)	0 (0%)
decreased appetite	15 (18.8%)	9 (11.3%)	0 (0%)	0 (0%)	0 (0%)	10 (11.8%)	5 (5.9%)	0 (0%)	0 (0%)	0 (0%)
dehydration	0 (0%)	0 (0%)	1 (1.3%)	0 (0%)	0 (0%)	0 (0%)	0 (0%)	0 (0%)	0 (0%)	0 (0%)
dyspepsia	0 (0%)	0 (0%)	0 (0%)	0 (0%)	0 (0%)	1 (1.2%)	0 (0%)	0 (0%)	0 (0%)	0 (0%)
dysphagia	0 (0%)	0 (0%)	0 (0%)	0 (0%)	0 (0%)	1 (1.2%)	0 (0%)	0 (0%)	0 (0%)	0 (0%)
hyperalbuminemia	1 (1.3%)	0 (0%)	0 (0%)	0 (0%)	0 (0%)	0 (0%)	0 (0%)	0 (0%)	0 (0%)	0 (0%)
hypercalcaemia	0 (0%)	0 (0%)	0 (0%)	0 (0%)	0 (0%)	1 (1.2%)	0 (0%)	0 (0%)	0 (0%)	0 (0%)
hyperkalaemia	2 (2.5%)	0 (0%)	0 (0%)	0 (0%)	0 (0%)	1 (1.2%)	0 (0%)	0 (0%)	0 (0%)	0 (0%)
hypoalbuminaemia	1 (1.3%)	0 (0%)	0 (0%)	0 (0%)	0 (0%)	2 (2.4%)	0 (0%)	0 (0%)	0 (0%)	0 (0%)
hypocalcaemia	1 (1.3%)	0 (0%)	0 (0%)	0 (0%)	0 (0%)	0 (0%)	0 (0%)	1 (1.2%)	0 (0%)	0 (0%)
hypoglycaemia	0 (0%)	0 (0%)	0 (0%)	0 (0%)	0 (0%)	1 (1.2%)	0 (0%)	0 (0%)	0 (0%)	0 (0%)
hypokalaemia	0 (0%)	0 (0%)	0 (0%)	0 (0%)	0 (0%)	1 (1.2%)	0 (0%)	0 (0%)	0 (0%)	0 (0%)
hypomagnesemia	1 (1.3%)	0 (0%)	0 (0%)	0 (0%)	0 (0%)	2 (2.4%)	0 (0%)	0 (0%)	0 (0%)	0 (0%)
hyponatraemia	0 (0%)	0 (0%)	1 (1.3%)	0 (0%)	0 (0%)	1 (1.2%)	0 (0%)	0 (0%)	1 (1.2%)	0 (0%)
weight decreased	2 (2.5%)	3 (3.8%)	0 (0%)	0 (0%)	0 (0%)	1 (1.2%)	1 (1.2%)	0 (0%)	0 (0%)	0 (0%)
weight increased	1 (1.3%)	0 (0%)	0 (0%)	0 (0%)	0 (0%)	0 (0%)	0 (0%)	0 (0%)	0 (0%)	0 (0%)
Musculoskeletal and connective tissue disorders										
arthralgia	13 (16.25%)	2 (2.5%)	0 (0%)	0 (0%)	0 (0%)	15 (17.6%)	8 (9.4%)	0 (0%)	0 (0%)	0 (0%)
axillary pain	1 (1.3%)	0 (0%)	0 (0%)	0 (0%)	0 (0%)	0 (0%)	0 (0%)	0 (0%)	0 (0%)	0 (0%)
back pain	4 (5%)	6 (7.5%)	0 (0%)	0 (0%)	0 (0%)	13 (15.3%)	3 (3.5%)	0 (0%)	0 (0%)	0 (0%)
bone pain	1 (1.3%)	0 (0%)	0 (0%)	0 (0%)	0 (0%)	3 (3.5%)	1 (1.2%)	0 (0%)	0 (0%)	0 (0%)
burning sensation	0 (0%)	0 (0%)	0 (0%)	0 (0%)	0 (0%)	1 (1.2%)	0 (0%)	0 (0%)	0 (0%)	0 (0%)
chest discomfort	0 (0%)	0 (0%)	0 (0%)	0 (0%)	0 (0%)	1 (1.2%)	0 (0%)	0 (0%)	0 (0%)	0 (0%)
chest pain	0 (0%)	0 (0%)	0 (0%)	0 (0%)	0 (0%)	1 (1.2%)	0 (0%)	0 (0%)	0 (0%)	0 (0%)
costochondritis	0 (0%)	1 (1.3%)	0 (0%)	0 (0%)	0 (0%)	2 (2.4%)	0 (0%)	0 (0%)	0 (0%)	0 (0%)
flank pain	0 (0%)	0 (0%)	0 (0%)	0 (0%)	0 (0%)	1 (1.2%)	0 (0%)	0 (0%)	0 (0%)	0 (0%)

groin pain	1 (1.3%)	0 (0%)	0 (0%)	0 (0%)	0 (0%)	2 (2.4%)	0 (0%)	0 (0%)	0 (0%)	0 (0%)
ligament rupture	0 (0%)	0 (0%)	0 (0%)	0 (0%)	0 (0%)	1 (1.2%)	0 (0%)	0 (0%)	0 (0%)	0 (0%)
mobility decreased	0 (0%)	0 (0%)	0 (0%)	0 (0%)	0 (0%)	0 (0%)	1 (1.2%)	0 (0%)	0 (0%)	0 (0%)
muscle spasms	5 (6.25%)	0 (0%)	0 (0%)	0 (0%)	0 (0%)	5 (5.9%)	0 (0%)	0 (0%)	0 (0%)	0 (0%)
muscle twitching	1 (1.3%)	0 (0%)	0 (0%)	0 (0%)	0 (0%)	0 (0%)	0 (0%)	0 (0%)	0 (0%)	0 (0%)
muscoskeletal pain	4 (5%)	4 (5%)	0 (0%)	0 (0%)	0 (0%)	4 (4.7%)	3 (3.5%)	0 (0%)	0 (0%)	0 (0%)
muscular weakness	1 (1.3%)	1 (1.3%)	1 (1.3%)	0 (0%)	0 (0%)	0 (0%)	0 (0%)	0 (0%)	0 (0%)	0 (0%)
musculoskeletal chest pain	2 (2.5%)	1 (1.3%)	0 (0%)	0 (0%)	0 (0%)	1 (1.2%)	2 (2.4%)	0 (0%)	0 (0%)	0 (0%)
musculoskeletal pain	6 (7.5%)	1 (1.3%)	0 (0%)	0 (0%)	0 (0%)	8 (9.4%)	1 (1.2%)	0 (0%)	0 (0%)	0 (0%)
myalgia	2 (2.5%)	2 (2.5%)	0 (0%)	0 (0%)	0 (0%)	5 (5.9%)	1 (1.2%)	0 (0%)	0 (0%)	0 (0%)
neck pain	2 (2.5%)	1 (1.3%)	0 (0%)	0 (0%)	0 (0%)	3 (3.5%)	0 (0%)	0 (0%)	0 (0%)	0 (0%)
osteomyelitis	0 (0%)	1 (1.3%)	0 (0%)	0 (0%)	0 (0%)	0 (0%)	0 (0%)	0 (0%)	0 (0%)	0 (0%)
osteonecrosis	0 (0%)	1 (1.3%)	0 (0%)	0 (0%)	0 (0%)	0 (0%)	1 (1.2%)	0 (0%)	0 (0%)	0 (0%)
pain	0 (0%)	1 (1.3%)	0 (0%)	0 (0%)	0 (0%)	0 (0%)	1 (1.2%)	0 (0%)	0 (0%)	0 (0%)
pain in extremity	3 (3.8%)	0 (0%)	0 (0%)	0 (0%)	0 (0%)	1 (1.2%)	0 (0%)	0 (0%)	0 (0%)	0 (0%)
pelvic pain	0 (0%)	0 (0%)	0 (0%)	0 (0%)	0 (0%)	0 (0%)	1 (1.2%)	0 (0%)	0 (0%)	0 (0%)
sciatica	0 (0%)	0 (0%)	0 (0%)	0 (0%)	0 (0%)	1 (1.2%)	1 (1.2%)	0 (0%)	0 (0%)	0 (0%)
spinal cord compression	0 (0%)	0 (0%)	1 (1.3%)	1 (1.3%)	0 (0%)	0 (0%)	0 (0%)	0 (0%)	0 (0%)	0 (0%)
Neoplasms benign, malignant and unspecified (incl cysts and polyps)										
bone pain	0 (0%)	0 (0%)	1 (1.3%)	0 (0%)	0 (0%)	0 (0%)	0 (0%)	0 (0%)	0 (0%)	0 (0%)
cancer pain	1 (1.3%)	0 (0%)	0 (0%)	0 (0%)	0 (0%)	0 (0%)	0 (0%)	0 (0%)	0 (0%)	0 (0%)
Nervous system disorders										
aphasia	0 (0%)	0 (0%)	0 (0%)	0 (0%)	0 (0%)	0 (0%)	1 (1.2%)	0 (0%)	0 (0%)	0 (0%)
convulsion	0 (0%)	0 (0%)	0 (0%)	0 (0%)	0 (0%)	1 (1.2%)	0 (0%)	0 (0%)	0 (0%)	0 (0%)
dizziness	9 (11.3%)	2 (2.5%)	0 (0%)	0 (0%)	0 (0%)	5 (5.9%)	1 (1.2%)	0 (0%)	0 (0%)	0 (0%)
dysaesthesia	1 (1.3%)	0 (0%)	0 (0%)	0 (0%)	0 (0%)	0 (0%)	0 (0%)	0 (0%)	0 (0%)	0 (0%)
dysarthria	0 (0%)	0 (0%)	0 (0%)	0 (0%)	0 (0%)	2 (2.4%)	0 (0%)	0 (0%)	0 (0%)	0 (0%)
dysgeusia	6 (7.5%)	2 (2.5%)	0 (0%)	0 (0%)	0 (0%)	0 (0%)	0 (0%)	0 (0%)	0 (0%)	0 (0%)
dyskinesia	0 (0%)	1 (1.3%)	0 (0%)	0 (0%)	0 (0%)	0 (0%)	0 (0%)	0 (0%)	0 (0%)	0 (0%)
encephalopathy	0 (0%)	0 (0%)	0 (0%)	0 (0%)	0 (0%)	0 (0%)	0 (0%)	0 (0%)	1 (1.2%)	0 (0%)
facial nerve disorder	0 (0%)	1 (1.3%)	0 (0%)	0 (0%)	0 (0%)	0 (0%)	0 (0%)	0 (0%)	0 (0%)	0 (0%)
gait disturbance	0 (0%)	0 (0%)	0 (0%)	0 (0%)	0 (0%)	1 (1.2%)	0 (0%)	0 (0%)	0 (0%)	0 (0%)
haemorrhage intracranial	0 (0%)	1 (1.3%)	0 (0%)	0 (0%)	1 (1.3%)	0 (0%)	0 (0%)	0 (0%)	0 (0%)	0 (0%)
headache	16 (20.0%)	4 (5%)	1 (1.3%)	0 (0%)	0 (0%)	19 (22.4%)	2 (2.4%)	0 (0%)	0 (0%)	0 (0%)
hypoaesthesia	1 (1.3%)	0 (0%)	0 (0%)	0 (0%)	0 (0%)	2 (2.4%)	0 (0%)	0 (0%)	0 (0%)	0 (0%)
lethargy	0 (0%)	3 (3.75%)	0 (0%)	0 (0%)	0 (0%)	1 (1.2%)	0 (0%)	0 (0%)	0 (0%)	0 (0%)

migraine	1 (1.3%)	0 (0%)	0 (0%)	0 (0%)	0 (0%)	0 (0%)	0 (0%)	0 (0%)	0 (0%)	0 (0%)
muscle spasms	1 (1.3%)	0 (0%)	0 (0%)	0 (0%)	0 (0%)	1 (1.2%)	0 (0%)	0 (0%)	0 (0%)	0 (0%)
myalgia	0 (0%)	0 (0%)	0 (0%)	0 (0%)	0 (0%)	1 (1.2%)	0 (0%)	0 (0%)	0 (0%)	0 (0%)
myelitis	0 (0%)	0 (0%)	1 (1.3%)	0 (0%)	0 (0%)	0 (0%)	0 (0%)	0 (0%)	0 (0%)	0 (0%)
myokymia	0 (0%)	0 (0%)	0 (0%)	0 (0%)	0 (0%)	1 (1.2%)	0 (0%)	0 (0%)	0 (0%)	0 (0%)
nervousness	1 (1.3%)	1 (1.3%)	0 (0%)	0 (0%)	0 (0%)	0 (0%)	0 (0%)	0 (0%)	0 (0%)	0 (0%)
neuralgia	2 (2.5%)	0 (0%)	0 (0%)	0 (0%)	0 (0%)	0 (0%)	1 (1.2%)	0 (0%)	0 (0%)	0 (0%)
neuropathy peripheral	5 (6.3%)	1 (1.3%)	0 (0%)	0 (0%)	0 (0%)	1 (1.2%)	2 (2.4%)	0 (0%)	0 (0%)	0 (0%)
night sweats	1 (1.3%)	0 (0%)	0 (0%)	0 (0%)	0 (0%)	0 (0%)	0 (0%)	0 (0%)	0 (0%)	0 (0%)
paraesthesia	2 (2.5%)	1 (1.3%)	0 (0%)	0 (0%)	0 (0%)	5 (5.9%)	0 (0%)	0 (0%)	0 (0%)	0 (0%)
peripheral sensory neuropathy	0 (0%)	0 (0%)	0 (0%)	0 (0%)	0 (0%)	1 (1.2%)	0 (0%)	0 (0%)	0 (0%)	0 (0%)
seizure	0 (0%)	1 (1.3%)	0 (0%)	0 (0%)	0 (0%)	1 (1.2%)	0 (0%)	0 (0%)	0 (0%)	0 (0%)
somnolence	1 (1.3%)	1 (1.3%)	0 (0%)	0 (0%)	0 (0%)	0 (0%)	0 (0%)	0 (0%)	0 (0%)	0 (0%)
spinal cord compression	0 (0%)	0 (0%)	1 (1.3%)	0 (0%)	0 (0%)	0 (0%)	0 (0%)	0 (0%)	0 (0%)	0 (0%)
syncope	1 (1.3%)	0 (0%)	0 (0%)	0 (0%)	0 (0%)	1 (1.2%)	0 (0%)	0 (0%)	0 (0%)	0 (0%)
Psychiatric disorders										
anxiety	2 (2.5%)	1 (1.3%)	0 (0%)	0 (0%)	0 (0%)	1 (1.2%)	0 (0%)	0 (0%)	0 (0%)	0 (0%)
confusional state	1 (1.3%)	1 (1.3%)	0 (0%)	0 (0%)	0 (0%)	0 (0%)	1 (1.2%)	1 (1.2%)	0 (0%)	0 (0%)
depressed mood	3 (3.8%)	1 (1.3%)	0 (0%)	0 (0%)	0 (0%)	4 (4.7%)	1 (1.2%)	0 (0%)	0 (0%)	0 (0%)
depression	2 (2.5%)	0 (0%)	0 (0%)	0 (0%)	0 (0%)	0 (0%)	0 (0%)	0 (0%)	0 (0%)	0 (0%)
insomnia	4 (5%)	6 (7.5%)	0 (0%)	0 (0%)	0 (0%)	3 (3.5%)	2 (2.4%)	0 (0%)	0 (0%)	0 (0%)
mood altered	1 (1.3%)	0 (0%)	0 (0%)	0 (0%)	0 (0%)	0 (0%)	0 (0%)	0 (0%)	0 (0%)	0 (0%)
panic attacks	0 (0%)	1 (1.3%)	0 (0%)	0 (0%)	0 (0%)	1 (1.2%)	0 (0%)	0 (0%)	0 (0%)	0 (0%)
poor quality sleep	0 (0%)	0 (0%)	0 (0%)	0 (0%)	0 (0%)	1 (1.2%)	0 (0%)	0 (0%)	0 (0%)	0 (0%)
restless legs syndrome	0 (0%)	0 (0%)	0 (0%)	0 (0%)	0 (0%)	1 (1.2%)	0 (0%)	0 (0%)	0 (0%)	0 (0%)
restlessness	1 (1.3%)	0 (0%)	0 (0%)	0 (0%)	0 (0%)	0 (0%)	0 (0%)	0 (0%)	0 (0%)	0 (0%)
Renal and urinary disorders										
blood creatinine increased	0 (0%)	0 (0%)	0 (0%)	0 (0%)	0 (0%)	1 (1.2%)	0 (0%)	0 (0%)	0 (0%)	0 (0%)
cystitis	1 (1.3%)	0 (0%)	0 (0%)	0 (0%)	0 (0%)	2 (2.4%)	0 (0%)	0 (0%)	0 (0%)	0 (0%)
dysuria	1 (1.3%)	0 (0%)	0 (0%)	0 (0%)	0 (0%)	0 (0%)	0 (0%)	0 (0%)	0 (0%)	0 (0%)
glomerular filtration rate decreased	1 (1.3%)	1 (1.3%)	1 (1.3%)	0 (0%)	0 (0%)	1 (1.2%)	0 (0%)	0 (0%)	0 (0%)	0 (0%)
haematuria	0 (0%)	0 (0%)	0 (0%)	0 (0%)	0 (0%)	0 (0%)	1 (1.2%)	0 (0%)	0 (0%)	0 (0%)
hydronephrosis repair	0 (0%)	1 (1.3%)	0 (0%)	0 (0%)	0 (0%)	0 (0%)	0 (0%)	0 (0%)	0 (0%)	0 (0%)
pollakiuria	0 (0%)	0 (0%)	0 (0%)	0 (0%)	0 (0%)	1 (1.2%)	0 (0%)	0 (0%)	0 (0%)	0 (0%)
renal failure acute	1 (1.3%)	0 (0%)	0 (0%)	0 (0%)	0 (0%)	0 (0%)	0 (0%)	0 (0%)	0 (0%)	0 (0%)
renal failure chronic	0 (0%)	1 (1.3%)	0 (0%)	0 (0%)	0 (0%)	0 (0%)	0 (0%)	0 (0%)	0 (0%)	0 (0%)
urinary incontinence	1 (1.3%)	0 (0%)	0 (0%)	0 (0%)	0 (0%)	0 (0%)	0 (0%)	0 (0%)	0 (0%)	0 (0%)

urinary tract infection	1 (1.3%)	2 (2.5%)	0 (0%)	0 (0%)	0 (0%)	0 (0%)	0 (0%)	0 (0%)	0 (0%)	0 (0%)
Reproductive system and breast disorders										
bartholin's abscess	0 (0%)	0 (0%)	0 (0%)	0 (0%)	0 (0%)	0 (0%)	1 (1.2%)	0 (0%)	0 (0%)	0 (0%)
breast cancer in situ	0 (0%)	0 (0%)	0 (0%)	0 (0%)	0 (0%)	0 (0%)	0 (0%)	1 (1.2%)	0 (0%)	0 (0%)
breast pain	1 (1.3%)	0 (0%)	0 (0%)	0 (0%)	0 (0%)	0 (0%)	0 (0%)	0 (0%)	0 (0%)	0 (0%)
erythema	1 (1.3%)	0 (0%)	0 (0%)	0 (0%)	0 (0%)	0 (0%)	0 (0%)	0 (0%)	0 (0%)	0 (0%)
pelvic pain	0 (0%)	0 (0%)	0 (0%)	0 (0%)	0 (0%)	1 (1.2%)	0 (0%)	0 (0%)	0 (0%)	0 (0%)
vaginal haemorrhage	0 (0%)	0 (0%)	0 (0%)	0 (0%)	0 (0%)	1 (1.2%)	0 (0%)	0 (0%)	0 (0%)	0 (0%)
vulvovsginal dryness	0 (0%)	0 (0%)	0 (0%)	0 (0%)	0 (0%)	1 (1.2%)	0 (0%)	0 (0%)	0 (0%)	0 (0%)
Respiratory, thoracic and mediastinal disorders										
aphonia	1 (1.3%)	0 (0%)	0 (0%)	0 (0%)	0 (0%)	0 (0%)	1 (1.2%)	0 (0%)	0 (0%)	0 (0%)
chest discomfort	2 (2.5%)	0 (0%)	0 (0%)	0 (0%)	0 (0%)	0 (0%)	0 (0%)	0 (0%)	0 (0%)	0 (0%)
chest pain	0 (0%)	0 (0%)	0 (0%)	0 (0%)	0 (0%)	2 (2.4%)	0 (0%)	0 (0%)	0 (0%)	0 (0%)
cough	12 (15.0%)	5 (6.3%)	0 (0%)	0 (0%)	0 (0%)	7 (8.2%)	5 (5.9%)	0 (0%)	0 (0%)	0 (0%)
dysphonia	2 (2.5%)	0 (0%)	0 (0%)	0 (0%)	0 (0%)	0 (0%)	1 (1.2%)	0 (0%)	0 (0%)	0 (0%)
dyspnoea	13 (16.3%)	4 (5%)	1 (1.3%)	0 (0%)	0 (0%)	11 (12.9%)	6 (7.1%)	2 (2.4%)	0 (0%)	0 (0%)
epistaxis	2 (2.5%)	0 (0%)	0 (0%)	0 (0%)	0 (0%)	0 (0%)	0 (0%)	0 (0%)	0 (0%)	0 (0%)
hypoxia	0 (0%)	0 (0%)	0 (0%)	0 (0%)	0 (0%)	0 (0%)	1 (1.2%)	0 (0%)	0 (0%)	0 (0%)
lower respiratory tract infection	0 (0%)	1 (1.3%)	0 (0%)	0 (0%)	0 (0%)	0 (0%)	0 (0%)	0 (0%)	0 (0%)	0 (0%)
lung infection	0 (0%)	0 (0%)	1 (1.3%)	0 (0%)	0 (0%)	0 (0%)	0 (0%)	0 (0%)	0 (0%)	0 (0%)
mucosal inflammation	0 (0%)	0 (0%)	0 (0%)	0 (0%)	0 (0%)	1 (1.2%)	0 (0%)	0 (0%)	0 (0%)	0 (0%)
nasal congestion	0 (0%)	0 (0%)	0 (0%)	0 (0%)	0 (0%)	1 (1.2%)	0 (0%)	0 (0%)	0 (0%)	0 (0%)
nasal discomfort	1 (1.3%)	0 (0%)	0 (0%)	0 (0%)	0 (0%)	0 (0%)	0 (0%)	0 (0%)	0 (0%)	0 (0%)
nasal dryness	1 (1.3%)	0 (0%)	0 (0%)	0 (0%)	0 (0%)	0 (0%)	0 (0%)	0 (0%)	0 (0%)	0 (0%)
nasopharyngitis	1 (1.3%)	0 (0%)	0 (0%)	0 (0%)	0 (0%)	0 (0%)	0 (0%)	0 (0%)	0 (0%)	0 (0%)
non-cardiac chest pain	0 (0%)	0 (0%)	0 (0%)	0 (0%)	0 (0%)	1 (1.2%)	0 (0%)	0 (0%)	0 (0%)	0 (0%)
oropharyngeal pain	5 (6.3%)	0 (0%)	0 (0%)	0 (0%)	0 (0%)	2 (2.4%)	0 (0%)	0 (0%)	0 (0%)	0 (0%)
pleural effusion	0 (0%)	1 (1.3%)	1 (1.3%)	0 (0%)	0 (0%)	2 (2.4%)	1 (1.2%)	4 (4.7%)	0 (0%)	0 (0%)
pleuritic pain	0 (0%)	0 (0%)	0 (0%)	0 (0%)	0 (0%)	1 (1.2%)	1 (1.2%)	0 (0%)	0 (0%)	0 (0%)
productive cough	0 (0%)	0 (0%)	0 (0%)	0 (0%)	0 (0%)	2 (2.4%)	0 (0%)	0 (0%)	0 (0%)	0 (0%)
prothrombin time prolonged	0 (0%)	0 (0%)	0 (0%)	0 (0%)	0 (0%)	1 (1.2%)	0 (0%)	0 (0%)	0 (0%)	0 (0%)
pulmonary embolism	0 (0%)	0 (0%)	0 (0%)	0 (0%)	0 (0%)	0 (0%)	0 (0%)	1 (1.2%)	0 (0%)	0 (0%)
pulmonary fibrosis	1 (1.3%)	0 (0%)	0 (0%)	0 (0%)	0 (0%)	0 (0%)	0 (0%)	0 (0%)	0 (0%)	0 (0%)
respiratory failure	0 (0%)	0 (0%)	0 (0%)	0 (0%)	0 (0%)	0 (0%)	0 (0%)	0 (0%)	1 (1.2%)	0 (0%)
sneezing	1 (1.3%)	0 (0%)	0 (0%)	0 (0%)	0 (0%)	0 (0%)	0 (0%)	0 (0%)	0 (0%)	0 (0%)
upper respiratory tract infection	0 (0%)	0 (0%)	0 (0%)	0 (0%)	0 (0%)	0 (0%)	1 (1.2%)	0 (0%)	0 (0%)	0 (0%)

wheezing	0 (0%)	0 (0%)	0 (0%)	0 (0%)	0 (0%)	1 (1.2%)	0 (0%)	0 (0%)	0 (0%)	0 (0%)
Skin and subcutaneous tissue disorders										
acne	1 (1.3%)	0 (0%)	0 (0%)	0 (0%)	0 (0%)	0 (0%)	0 (0%)	0 (0%)	0 (0%)	0 (0%)
alopecia	4 (5%)	0 (0%)	0 (0%)	0 (0%)	0 (0%)	2 (2.4%)	1 (1.2%)	0 (0%)	0 (0%)	0 (0%)
burning sensation	0 (0%)	0 (0%)	0 (0%)	0 (0%)	0 (0%)	1 (1.2%)	0 (0%)	0 (0%)	0 (0%)	0 (0%)
decubitius ulcer	0 (0%)	0 (0%)	0 (0%)	0 (0%)	0 (0%)	1 (1.2%)	0 (0%)	0 (0%)	0 (0%)	0 (0%)
dermatitis acneiform	3 (3.8%)	0 (0%)	0 (0%)	0 (0%)	0 (0%)	1 (1.2%)	0 (0%)	0 (0%)	0 (0%)	0 (0%)
dry skin	8 (10%)	1 (1.3%)	0 (0%)	0 (0%)	0 (0%)	6 (7.1%)	0 (0%)	0 (0%)	0 (0%)	0 (0%)
erythema	1 (1.3%)	0 (0%)	0 (0%)	0 (0%)	0 (0%)	1 (1.2%)	0 (0%)	0 (0%)	0 (0%)	0 (0%)
folliculitis	0 (0%)	0 (0%)	0 (0%)	0 (0%)	0 (0%)	1 (1.2%)	0 (0%)	0 (0%)	0 (0%)	0 (0%)
foreign body trauma	1 (1.3%)	0 (0%)	0 (0%)	0 (0%)	0 (0%)	0 (0%)	0 (0%)	0 (0%)	0 (0%)	0 (0%)
generalised oedema	0 (0%)	0 (0%)	0 (0%)	0 (0%)	0 (0%)	1 (1.2%)	0 (0%)	0 (0%)	0 (0%)	0 (0%)
hair texture abnormal	1 (1.3%)	0 (0%)	0 (0%)	0 (0%)	0 (0%)	0 (0%)	0 (0%)	0 (0%)	0 (0%)	0 (0%)
hot flush	2 (2.5%)	0 (0%)	0 (0%)	0 (0%)	0 (0%)	0 (0%)	0 (0%)	0 (0%)	0 (0%)	0 (0%)
lip blister	0 (0%)	1 (1.3%)	0 (0%)	0 (0%)	0 (0%)	0 (0%)	0 (0%)	0 (0%)	0 (0%)	0 (0%)
milia	2 (2.5%)	1 (1.3%)	0 (0%)	0 (0%)	0 (0%)	2 (2.4%)	0 (0%)	0 (0%)	0 (0%)	0 (0%)
nail disorder	2 (2.5%)	0 (0%)	0 (0%)	0 (0%)	0 (0%)	0 (0%)	0 (0%)	0 (0%)	0 (0%)	0 (0%)
oedema peripheral	0 (0%)	0 (0%)	0 (0%)	0 (0%)	0 (0%)	1 (1.2%)	0 (0%)	0 (0%)	0 (0%)	0 (0%)
onychoclasis	1 (1.3%)	0 (0%)	0 (0%)	0 (0%)	0 (0%)	0 (0%)	0 (0%)	0 (0%)	0 (0%)	0 (0%)
oral herpes	1 (1.3%)	0 (0%)	0 (0%)	0 (0%)	0 (0%)	1 (1.2%)	0 (0%)	0 (0%)	0 (0%)	0 (0%)
oral pain	1 (1.3%)	0 (0%)	0 (0%)	0 (0%)	0 (0%)	0 (0%)	0 (0%)	0 (0%)	0 (0%)	0 (0%)
pain in extremity	0 (0%)	0 (0%)	0 (0%)	0 (0%)	0 (0%)	1 (1.2%)	0 (0%)	0 (0%)	0 (0%)	0 (0%)
pain of skin	1 (1.3%)	1 (1.3%)	0 (0%)	0 (0%)	0 (0%)	0 (0%)	0 (0%)	0 (0%)	0 (0%)	0 (0%)
palmar-plantar erythrodysaesthesia syndrome	2 (2.5%)	0 (0%)	0 (0%)	0 (0%)	0 (0%)	1 (1.2%)	0 (0%)	0 (0%)	0 (0%)	0 (0%)
photosensitivity reaction	2 (2.5%)	0 (0%)	1 (1.3%)	0 (0%)	0 (0%)	0 (0%)	0 (0%)	0 (0%)	0 (0%)	0 (0%)
pruritus	2 (2.5%)	2 (2.5%)	0 (0%)	0 (0%)	0 (0%)	6 (7.1%)	0 (0%)	0 (0%)	0 (0%)	0 (0%)
rash	32 (40.0%)	18 (22.5%)	4 (5.0%)	1 (1.3%)	0 (0%)	20 (23.5%)	2 (2.4%)	0 (0%)	0 (0%)	0 (0%)
rash erythematous	0 (0%)	1 (1.3%)	0 (0%)	0 (0%)	0 (0%)	0 (0%)	0 (0%)	0 (0%)	0 (0%)	0 (0%)
skin necrosis	2 (2.5%)	0 (0%)	0 (0%)	0 (0%)	0 (0%)	0 (0%)	1 (1.2%)	0 (0%)	0 (0%)	0 (0%)
skin nodule	0 (0%)	0 (0%)	0 (0%)	0 (0%)	0 (0%)	1 (1.2%)	0 (0%)	0 (0%)	0 (0%)	0 (0%)
skin ulcer	0 (0%)	1 (1.3%)	0 (0%)	0 (0%)	0 (0%)	0 (0%)	0 (0%)	1 (1.2%)	0 (0%)	0 (0%)
subcutaneous nodule	0 (0%)	0 (0%)	0 (0%)	0 (0%)	0 (0%)	1 (1.2%)	0 (0%)	0 (0%)	0 (0%)	0 (0%)
sunburn	2 (2.5%)	0 (0%)	0 (0%)	0 (0%)	0 (0%)	1 (1.2%)	0 (0%)	0 (0%)	0 (0%)	0 (0%)
telangiectasia	0 (0%)	0 (0%)	0 (0%)	0 (0%)	0 (0%)	1 (1.2%)	0 (0%)	0 (0%)	0 (0%)	0 (0%)
toxic epidermal necrolysis	0 (0%)	0 (0%)	0 (0%)	1 (1.3%)	0 (0%)	0 (0%)	0 (0%)	0 (0%)	0 (0%)	0 (0%)
Social circumstances										

partner stress	0 (0%)	0 (0%)	0 (0%)	0 (0%)	0 (0%)	1 (1.2%)	0 (0%)	0 (0%)	0 (0%)	0 (0%)
Surgical and medical procedures										
dental operation	0 (0%)	0 (0%)	0 (0%)	0 (0%)	0 (0%)	0 (0%)	1 (1.2%)	0 (0%)	0 (0%)	0 (0%)
hip arthroplasty	0 (0%)	1 (1.3%)	0 (0%)	0 (0%)	0 (0%)	0 (0%)	0 (0%)	0 (0%)	0 (0%)	0 (0%)
tooth abscess	0 (0%)	0 (0%)	0 (0%)	0 (0%)	0 (0%)	0 (0%)	1 (1.2%)	0 (0%)	0 (0%)	0 (0%)
tooth extraction	0 (0%)	0 (0%)	0 (0%)	0 (0%)	0 (0%)	1 (1.2%)	0 (0%)	0 (0%)	0 (0%)	0 (0%)
Vascular disorders										
deep vein thrombosis	0 (0%)	0 (0%)	0 (0%)	0 (0%)	0 (0%)	0 (0%)	1 (1.2%)	0 (0%)	0 (0%)	0 (0%)
embolism	0 (0%)	0 (0%)	1 (1.3%)	0 (0%)	0 (0%)	0 (0%)	0 (0%)	0 (0%)	0 (0%)	0 (0%)
hot flush	13 (16.3%)	1 (1.3%)	0 (0%)	0 (0%)	0 (0%)	13 (15.3%)	1 (1.2%)	0 (0%)	0 (0%)	0 (0%)
hyperhidrosis	0 (0%)	1 (1.3%)	0 (0%)	0 (0%)	0 (0%)	1 (1.2%)	0 (0%)	0 (0%)	0 (0%)	0 (0%)
hypertension	4 (5%)	19 (23.8%)	6 (7.5%)	0 (0%)	0 (0%)	2 (2.4%)	6 (7.1%)	3 (3.5%)	0 (0%)	0 (0%)
lymphoedema	0 (0%)	0 (0%)	0 (0%)	0 (0%)	0 (0%)	0 (0%)	4 (4.7%)	0 (0%)	0 (0%)	0 (0%)
orthostatic hypotension	0 (0%)	1 (1.3%)	0 (0%)	0 (0%)	0 (0%)	0 (0%)	0 (0%)	0 (0%)	0 (0%)	0 (0%)
phlebitis	0 (0%)	1 (1.3%)	0 (0%)	0 (0%)	0 (0%)	0 (0%)	0 (0%)	0 (0%)	0 (0%)	0 (0%)

Table S2. FURVA recruiting centres

Trust	PI	Total recruits
NHS Lothian	Dr Larry Hayward	23
Royal United Hospital Bath	Dr Mark Beresford	18
Royal Devon and Exeter NHS Foundation Trust	Dr David Hwang	17
Somerset NHS Foundation Trust	Dr Saiqa Spensley	17
Velindre University NHS Trust	Dr Simon Waters	15
Royal Cornwall Hospital	Dr Duncan Wheatley	15
North West Anglia NHS Foundation Trust (Peterborough City Hospital)	Dr Karen McAdam	10
University Hospitals Dorset NHS Foundation Trust	Dr Tamas Hickish	9
Worcestershire Acute Hospitals NHS Trust	Dr Mark Churn	6
Sandwell and West Birmingham Hospitals	Dr Mariam Jafri	6
University Hospitals of Derby and Burton NHS Foundation Trust	Dr Mojca Persic	6
Gloucestershire Hospitals NHS Foundation Trust	Dr Peter Jenkins	5
University Hospitals Bristol and Weston NHS Foundation Trust (Bristol Haematology and Oncology Centre)	Dr Vivek Mohan	5
North West Anglia NHS Foundation Trust (Hinchingbrooke Hospital)	Dr Cheryl Palmer	5
The Beatson West of Scotland Cancer Centre	Dr Iain MacPherson	3
East Suffolk and North Essex NHS Foundation Trust	Dr Mukesh Bindlish Mukesh	3
University Hospitals Bristol and Weston NHS Foundation Trust (Weston General Hospital)	Dr Thomas Wells	2
Dorset County Hospital NHS Foundation Trust	Dr Amithaba Chakrabarti	0
King's College Hospital NHS Foundation Trust	Dr Mark Harries	0

Short Title: Fulvestrant +/- vandetanib in advanced aromatase inhibitor resistant breast cancer

Full Title: A randomised double blind placebo controlled phase II study of fulvestrant with or without the addition of vandetanib as treatment for patients with metastatic breast cancer resistant to aromatase inhibitor therapy

Clinical Trial Protocol

Version: 3.0

Date: 09 December 2016

EudraCT No: 2014-001208-23

ClinicalTrials.gov Number: NCT02530411

ISRCTN No.: ISRCTN13663157

Funder: CRUK/Astrazeneca

Funder No: CRUK/13/029

Name of Sponsor: Velindre NHS Trust

Sponsor No: 2014/VCC/0013

Authorised by:

Name:	Mark Beresford	Role:	Joint Chief Investigator
Signature:	______________________	Date:	09/12/2016
Name:	Robert Jones	Role:	Joint Chief Investigator
Signature:	______________________	Date:	09/12/2016
Name:	Richard Adams	Role:	Director, WCTU
Signature:	______________________	Date:	09/12/2016

Developed on behalf of the NCRI Breast Clinical Studies Group

CONFIDENTIAL

This material is the property of the Wales Cancer Trials Unit. Do not disclose or use except as authorised.

General Information

This protocol describes the FURVA clinical trial, and provides information about the procedures for entering participants into the trial. The protocol should not be used as a guide, or as an aide-memoire for the treatment of other patients. Every care has been taken in drafting this protocol; however, corrections or amendments may be necessary. These will be circulated to the known Investigators in the trial, but sites entering patients for the first time are advised to contact the Wales Cancer Trials Unit (WCTU) in Cardiff to confirm that they have the most up-to-date version of the protocol in their possession. Problems relating to the trial should be referred, in the first instance, to the WCTU.

Compliance

This trial will adhere to the conditions and principles of Good Clinical Practice which apply to all clinical trials as outlined in the Medicines for Human Use (Clinical Trials) Regulations 2004 (SI 1031), as amended, EU Directive 2001/20/EC, EU Directive 2005/28/EC. It will be conducted in compliance with the protocol, the Declaration of Helsinki (South Africa, 1996), the Research Governance Framework for Health and Social Care (Welsh Assembly Government 2009 and Department of Health 2nd July 2005), the Data Protection Act 1998, and other regulatory requirements as appropriate.

Funding

The FURVA trial is being jointly funded by the Clinical Trials Advisory and Awards Committee (CTACC), on behalf of Cancer Research UK and AstraZeneca.

SAE Reporting

Where the adverse event meets one of the serious categories, an SAE form should be completed by the responsible clinician and submitted to the Wales Cancer Trials Unit (WCTU) at the Centre for Trials Research (CTR), Cardiff University within 24 hours of becoming aware of the event (See section 10 for more details).

Email: CTR-Safety@cardiff.ac.uk

Fax (emergency use only) : 029 2064 4488

CONFIDENTIAL

This material is the property of the Wales Cancer Trials Unit. Do not disclose or use except as authorised.

Trial Co-ordination

The FURVA trial is being coordinated by the WCTU, a National Cancer Research Institute (NCRI) accredited, and United Kingdom Clinical Research Collaboration (UKCRC) registered trials unit.

This protocol has been developed by the FURVA Trial Management Group (TMG) on behalf of the NCRI Breast Clinical Studies Group (CSG).

Wales Cancer Trials Unit (WCTU)	Tel: +44 (0) 29 2068 7500
Centre for Trials Research (CTR)	Fax: +44 (0) 29 2068 7501
College of Biomedical & Life Sciences	
Cardiff University	Email: FURVA@cardiff.ac.uk
6th Floor	Website: www.cardiff.ac.uk/centre-for-trials-research
Neuadd Meirionnydd	
Heath Park	
Cardiff	
CF14 4YS	

FURVA trial staff

For all queries please contact the FURVA Trial Manager. Any clinical queries will be directed through the Trial Manager to either the Chief Investigator or one of the clinical Co-Investigators.

Trial Manager:	Dr Joanna Smith	Tel: +44 (0) 29 2068 790<br>Email: sealjd@cardiff.ac.uk
Senior Statistician:	Ms Angela Casbard	Email: casbardac@cardiff.ac.uk
Associate Clinical Director:	Prof Fergus MacBeth	Email: macbethfr@cardiff.ac.uk
Director:	Dr Richard Adams	Email: Richard.Adams@wales.nhs.uk
Safety Desk:		Tel: +44 (0) 29 2068 7469<br>Email: WCTU-safety@cardiff.ac.uk

This material is the property of the Wales Cancer Trials Unit. Do not disclose or use except as authorised.

Co-Chief Investigators

Dr Mark Beresford

Department of Oncology, Royal United Hospital, Combe Park, Bath BA1 3NG

Dr Robert Jones

Velindre Hospital, Velindre Road, Cardiff CF14 2TL

Co-investigators

Professor Peter Barrett-Lee

Velindre Hospital
Velindre Road
Cardiff
CF14 2TL

Professor Gareth Griffiths

Director
University of Southampton Clinical Trials Unit
Southampton General Hospital
Tremona Road
Southampton
Hants
SO16 6YD

Collaborators

Dr Sacha Howell

The University of Manchester
Department of Medical Oncology
The Christie NHS Foundation Trust
Manchester
M204BX

Professor David Cameron

The University of Edinburgh
Crewe Road South
Edinburgh
EH4 2XR

Nursing Advisor

Kay Wilson

Clinical Trials Unit
Velindre Cancer Centre
Whitchurch
Cardiff
CF14 2TL

Pharmacy Advisor

Bethan Tranter
Velindre Hospital
Velindre Road
Cardiff
CF14 2TL

This material is the property of the Wales Cancer Trials Unit. Do not disclose or use except as authorised.

Translational collaborator

Dr Julia Gee

Breast Cancer Molecular Pharmacology group
Cardiff School of Pharmacy & Pharmaceutical Sciences
Cardiff University
Room 2.54, Redwood Building
King Edward VII Avenue
Cardiff CF10 3NB

Consumer representatives

Lesley Radley

c/o WCTU

This material is the property of the Wales Cancer Trials Unit. Do not disclose or use except as authorised.

Table of contents

Abbreviations and glossary 9
1.0 Trial schema 12
2.0 Trial synopsis 13
2.1 Lay summary 20
3.0 Background, rationale and objectives 21
4.0 Study design 24
4.1 Risk assessment 25
5.0 Participating site selection 26
6.0 Participant eligibility 28
6.1 Screening procedures 28
6.2 Inclusion criteria 28
6.3 Exclusion criteria 30
6.4 Restrictions 31
6.5 Informed consent 32
7.0 Randomisation 34
8.0 Trial treatments 34
8.1 Fulvestrant and vandetanib/placebo 34
8.2 Blinding 45
8.3 Dose banding 46
9.0 Trial assessments 46
9.1 Screening assessments 46
9.2 Baseline assessments 47
9.3 Assessments on treatment (weeks 1-60) 47
9.4 Assessments on treatment (weeks 61+) 48
9.5 End of treatment assessments 48
9.6 End of study assessments 48
9.7 Unscheduled assessments 48
9.8 Completion of CRFs 49
9.9 Schedule of trial treatments, scheduled and unscheduled assessments, and translational sample collection timepoints 50
10.0 Safety reporting and pharmacovigilance 52

This material is the property of the Wales Cancer Trials Unit. Do not disclose or use except as authorised.

10.1 Causality Assessments 53
10.2 Expectedness Assessments........ 53
10.3 SAE reporting 54
10.4 SUSAR reporting 55
10.5 Safety Reports........ 56
10.6 Flowchart for Serious Adverse Event reporting 57
11.0 Trial management........ 58
11.1 Trial committees and trial management 58
11.2 Monitoring........ 58
11.3 Participant Withdrawal........ 58
11.4 Lost to follow-up........ 60
11.5 Protocol/GCP non-compliance 60
11.6 The End of the Trial........ 61
11.7 Archiving 61
12.0 Statistical considerations........ 62
12.1 Randomisation........ 62
12.2 Outcome measures........ 62
12.3 Sample size calculation........ 62
12.4 Statistical analyses........ 62
12.5 Sub-group analyses........ 63
13.0 Translational research 64
14.0 Publication policy........ 66
15.0 Ethical and regulatory considerations, and Informed Consent........ 67
15.1 Ethical approval 67
15.2 Clinical Trial Authorisation (CTA) 67
15.3 Regulatory considerations........ 67
15.4 Research governance approval 67
15.5 Sponsorship 67
15.6 Indemnity........ 68
15.7 Data protection........ 68
15.8 Finance........ 68
16.0 References 70
APPENDIX 1: RECIST v1.1 72

APPENDIX 2: New York Heart Association (NYHA) classification of heart disease 82
APPENDIX 3: A guide to performing causality assessments 83
APPENDIX 4: Cockcroft and Gault Formula to calculate GFR 84

CONFIDENTIAL

This material is the property of the Wales Cancer Trials Unit. Do not disclose or use except as authorised.

Abbreviations and glossary

**FURVA**	Fulvestrant +/- vandetanib in advanced aromatase inhibitor resistant breast cancer
**ABC**	Advanced Breast Cancer
**ABPI**	Association of the British Pharmaceutical Industry
**AE**	Adverse Event
**AI**	Aromatase Inhibitor
**ALP**	Alkaline phosphatase
**ALT**	Aspartate aminotransferase
**ANC**	Absolute Neutrophil Count
**AR**	Adverse reaction
**AST**	Alanine aminotransferase
**AWMGL**	All Wales Molecular Genetics Laboratory
**BC**	Breast Cancer
**C634W**	RET activating mutation in TT cell line
**CEP17**	Chromosome 17 centromere
**CI**	Chief Investigator
**CR**	Complete Response
**CRF**	Case Report Form
**CR-UK**	Cancer Research UK
**CSG**	Clinical Studies Group
**CT**	Computerised axial tomography
**CTA**	Clinical Trial Authorisation
**CTCAE**	Common Terminology Criteria for Adverse Events Version 4.03
**CTIMP**	Clinical Trial of an Investigational Medicinal Product.<br>A clinical trial that is within the scope of the UK Medicines for Human Use (Clinical Trials) Regulations 2004.
**CYP3A4**	Cytochrome P450 3A4
**DH**	Department of Health
**DSUR**	Development Safety Update Report
**ECG**	Electrocardiogram

This material is the property of the Wales Cancer Trials Unit. Do not disclose or use except as authorised.

**ECOG**	Eastern Cooperative Oncology Group
**EGFR**	Epidermal Growth Factor Receptor
**ER+ve**	Estrogen-receptor positive breast cancer
**EudraCT**	European Union Drug Regulatory Agency Clinical Trial
**FISH/CISH/D-DISH**	Fluorescence in situ hybridisation / Chromogenic in situ hybridisation/ Dual-colour dual-hapten brightfield in situ hybridisation
**FSH**	Follicle-Stimulating Hormone
**GCP**	Good Clinical Practice
**gDNA**	Genomic DNA
**GFR**	Glial Cell Line-Derived Neurotrophic Factor Receptor
**GP**	General Practitioner
**GnRH**	Gonadotropin-releasing hormone
**IB**	Investigator's Brochure
**IHC**	Immunohistochemistry
**IP**	Investigational Product
**IM**	Intramuscular
**INR**	International Normalized Ratio
**IWRS**	Interactive Web Response System
**HER2**	Human epidermal growth factor receptor 2
**Hgb**	Haemoglobin
**ICH-GCP**	International Conference on Harmonisation – Good Clinical Practice
**IDMC**	Independent Data Monitoring Committee
**IMP**	Investigational Medicinal Product<br>A pharmaceutical form of an active substance or placebo being tested or used in a clinical trial, including products already with a marketing authorisation, but used or assembled (formulated or packaged) in a way different to the authorised form, or when used for an unauthorised indication, or when used to gain more information about the authorised form.
**Individual**	An individual who may be eligible for the trial but has not yet consented to participate in any trial related activities.
**ISF**	Investigator Site File

This material is the property of the Wales Cancer Trials Unit. Do not disclose or use except as authorised.

**ISRCTN**	International Standard Randomised Controlled Trial Number
**KDR**	Kinase insert domain receptor
**MBC**	Metastatic Breast Cancer
**MHRA**	Medicines and Healthcare products Regulatory Agency
**MREC**	Main Research Ethics Committee
**MTC**	Medullary thyroid carcinoma
**NCIN**	National Cancer Intelligence Network
**NCRI**	National Cancer Research Institute
**NHS**	National Health Service
**NIHR**	National Institute for Health Research
**nIMP**	non-Investigational Medicinal Product<br>Medicinal products that are not the object of investigation (i.e. other than the tested product, placebo or active comparator) supplied to the patients participating in the trial and used in accordance with the protocol. E.g. background treatment, rescue medication.
**NTx**	N-telopeptide
**NYHA**	New York Heart Association
**od**	Once daily
**OS**	Overall Survival
**Patient**	A patient under care who may be eligible for the trial but has not yet consented to participate in any trial related activities.
**Participant**	An individual who has given written informed consent and is participating in trial related activities.
**PFS**	Progression-Free Survival
**PI**	Principal Investigator
**plts**	Platelets
**po**	*Per os*, meaning taken orally or by mouth
**PR**	Partial Response
**PT**	Prothrombin Time
**q28**	28-day cycle
**QT and QTc**	The QT interval in the heart's electrical cycle
**R&D**	Research and Development

This material is the property of the Wales Cancer Trials Unit. Do not disclose or use except as authorised.

**RECIST**	Response Evaluation Criteria in Solid Tumours
**RET**	[Receptor tyrosine kinase RET] REarranged during Transfection
**RSI**	Reference Safety Information (Investigators brochure or Summary of Products Characteristics)
**SAE**	Serious Adverse Event
**SAR**	Serious Adverse Reaction
**SD**	Stable disease
**siDNA**	Signal Interferring DNA (deoxyribonucleic acid)
**Sponsor**	The primary organisation that oversees and is responsible for the clinical trial
**SPC**	Summary of Product Characteristics
**SUSAR**	Suspected Unexpected Serious Adverse Reaction
**TMF**	Trial Master File
**TMG**	Trial Management Group
**TSC**	Trial Steering Committee
**TSF**	Trial Site File
**UKCRC**	United Kingdom Clinical Research Collaboration
**ULN**	Upper Limit Normal
**VEGFR-2**	Vascular Endothelial Growth Factor Receptor 2
**WCB**	Wales Cancer Bank
**WCTU**	Wales Cancer Trials Unit
**WNL**	Within Normal Limits
**WHO**	World Health Organisation

1.0 Trial schema

160 ER+/HER2-ve advanced breast cancer patients

Previous treatment with 1-3 lines of endocrine therapy

This material is the property of the Wales Cancer Trials Unit. Do not disclose or use except as authorised.

REGISTRATION / ENROLMENT/ RANDOMISATION 1:1

Arm 1

Fulvestrant 500mg IM D1, D15, then D1 of every 28 day cycle

Vandetanib 300 mg po daily

Clinician review D1, D15, weeks 4, 8, 12, 16, 20, 24 then 12 weekly. CT at week 8, 16, 24 then 12 weekly.

Arm 2

Fulvestrant 500mg IM D1, D15, then D1 of every 28 day cycle

Placebo po daily

Clinician review D1, D15, weeks 4, 8, 12, 16, 20, 24 then 12 weekly. CT at week 8, 16, 24 then 12 weekly.

Continue treatment until progression, excess toxicity or withdrawal

Safety review after 20 and 40 patients have completed 1 cycle of treatment

Primary outcome measure:

- Progression-free survival (PFS - time to event) based on Response Evaluation Criteria in Solid Tumours (RECIST) v1.1. - Time from randomisation to any progression and/or death (from any cause).

Secondary outcome measures:

- Safety, tolerability (side effects) and feasibility of use (number of participants requiring dose delays or reductions and/or treatment withdrawal).
- Objective response rate and clinical benefit rate as assessed by RECIST v1.1.
- Overall survival (OS), time from enrolment to death with those still alive censored at date last seen.
- Exploratory analysis: The influence of RET signalling pathway components expression on vandetanib activity.

CONFIDENTIAL

This material is the property of the Wales Cancer Trials Unit. Do not disclose or use except as authorised.

2.0 Trial synopsis

Study title:	A randomised, double blind, placebo controlled phase II study of fulvestrant with or without the addition of vandetanib as treatment for patients with metastatic breast cancer resistant to aromatase inhibitor therapy.				
Study acronym:	FURVA				
Short title:	Fulvestrant +/- vandetanib in advanced aromatase inhibitor resistant breast cancer.				
EudraCT No:	2014-001208-23				
ClinicalTrials.gov Number:	NCT02530411				
ISRCTN No:	ISRCTN13663157				
Funder:	AstraZeneca and Cancer Research UK	Funder's No:	CRUK/13/029		
Chief Investigator:	Mark Beresford and Robert Jones				
Sponsor:	Velindre NHS Trust	Sponsor No:	2014/VCC/0013		
Study period:	3 years	Phase:	II	Number of arms:	2
Number of participants:	160 patients				
Investigational Medicinal Product(s) (IMP)	Vandetanib				
Non-Investigational Medicinal Product(s) (nIMP)	Fulvestrant				

Objectives:

Primary

Progression-free survival (PFS - time to event) based on Response Evaluation Criteria in Solid Tumours (RECIST) v1.1. - Time from randomisation to any progression and/or death (from any cause).

Secondary

- Safety, tolerability (side effects) and feasibility of use (number of participants requiring dose delays or reductions and/or treatment withdrawal).
- Objective response rate and clinical benefit rate as assessed by RECIST v1.1.

This material is the property of the Wales Cancer Trials Unit. Do not disclose or use except as authorised.

- Overall survival (OS), time from enrolment to death with those still alive censored at date last seen.
- Exploratory analysis: The influence of RET signalling pathway components expression on vandetanib activity.

Main inclusion criteria:

- Adult female patients, aged ≥ 18 years.
- Post-menopausal women.
- Minimum life expectancy of 12 weeks.
- Histological confirmation of ER+ve, breast cancer.
- Histological confirmation of HER2 negative breast cancer.
- Clinical or histological confirmation of metastatic or locally advanced disease not amenable to surgical resection.
- ECOG performance status 0 to 2.
- Measurable or non-measurable disease.
- Adequate bone marrow, renal and hepatic function.
- Progressive disease whilst receiving an aromatase inhibitor (AI) for advanced breast cancer (ABC), although this does not need to be the most recent therapy, or relapsed with ABC whilst receiving an AI in the adjuvant setting.
- Radiological or objective clinical evidence of recurrence or progression on or after the last systemic therapy prior to enrolment.
- No more than 3 prior lines of endocrine therapy for ABC.
- No more than 1 line of chemotherapy for ABC.
- Suitable for further endocrine therapy.
- Provision of archival tumour sample for exploratory analysis.
- Informed consent.

Main exclusion criteria:

- Previous treatment with fulvestrant or RET inhibitor therapy.
- Treatment with chemotherapy, immunotherapy or targeted, biologic or tumour embolisation within 21 days of study drug administration.
- Palliative radiotherapy within 7 days of study drug.
- Rapidly progressive visceral disease not suitable for further endocrine therapy.

CONFIDENTIAL

This material is the property of the Wales Cancer Trials Unit. Do not disclose or use except as authorised.

- Spinal cord compression or brain/leptomeningeal metastases that have not been controlled with surgery or radiotherapy.
- Any co-existing medical condition precluding trial entry including significant cardiac disease:
- Significant cardiac event
- History of arrhythmia
- QTc prolongation
- The following electrolyte values (the rationale is due to the increased risk of prolonged QTc):
  - Potassium <4.0 mmol/L, despite supplementation during screening, or above the CTCAE v4.03 Grade 1 upper limit
  - Magnesium below the normal range or above the CTCAE v4.03 Grade 1 upper limit
  - Calcium (ionised or serum) below the normal range despite supplementation, or above the Grade 1 upper limit
- Creatinine clearance <30 ml/min
- Major surgery within 4 weeks before the first dose of study treatment.
- Evidence of severe or uncontrolled systemic diseases
- Elevated ALP in the absence of bone metastasis
- History of hypersensitivity to vandetanib or fulvestrant
- Unresolved toxicities from prior therapy
- Evidence of dementia, altered mental status or any psychiatric condition that would prohibit understanding or rendering of informed consent.
- Participation in another clinical study with an investigational product (IP) during the last 30 days.
- Inability or unwillingness to comply with study procedures, including the inability to take regular oral medication.
- Previous or concomitant malignancies at any other site except:
  - Benign basal cell carcinoma.
  - Benign low grade transitional cell carcinoma of the bladder.
  - Other effectively treated malignancy that has been in remission for more than 5 years and is considered to be cured.

Treatments: 16 x 28 day Cycles

Arm 1

Fulvestrant 500mg intramuscular (IM) D1, D15, then D1 of each 28 day cycle

This material is the property of the Wales Cancer Trials Unit. Do not disclose or use except as authorised.

Vandetanib 300 mg po daily

Arm 2 (control arm)

Fulvestrant 500mg IM D1, D15, then D1 of each 28 day cycle

Placebo po daily

Trial assessments:

Screening (inclusive of eligibility screening collected after participant consent – within 28 days of date of randomisation)

- Medical History
- Physical examination
- ECOG performance status
- Concomitant medications
- Co-morbidities
- Collection of screening blood samples for screening toxicity assessment
- Screening cardiac assessments (ECG) repeated 3 times
- Clinical disease assessment
- Radiological disease assessment

Baseline (after consent and randomisation and prior to Cycle 1 Day 1 treatment)

- Physical examination
- ECOG performance status
- Concomitant medications
- Collection of baseline blood samples (within 72 hours prior to Cycle 1 Day 1 treatment)
- Baseline toxicity assessment
- Collection of NHS IC flagging data (only if consent given)
- Collection of timepoint 1 (baseline) translational blood sample
- Collection of archival tumour tissue sample (paraffin block)

Treatment weeks 1-60 (assessed until disease progression)

Participants will be reviewed by a clinician on Cycle 1, Days 1 and 15 and on weeks 4, 8, 12, 16, 20, 24 and then every 12 weeks. A trials nurse will review the participant at each fulvestrant administration. CT scans will be performed at 8, 16 and 24 weeks after

This material is the property of the Wales Cancer Trials Unit. Do not disclose or use except as authorised.

randomisation and at 12 weekly intervals thereafter. Participants will be treated until disease progression (or unacceptable toxicity or withdrawal of consent). The following assessments will be made:

- Treatment details including medication kit numbers
- Physical examination
- ECOG performance status
- Concomitant medication
- Blood tests: full blood count, urea and electrolytes (including potassium), liver function tests, calcium and magnesium
- Toxicity assessment
- ECG repeated 3 times (weeks 2, 4, 8, 24, 36, 48, 60)
- Clinical disease assessment (weeks 2, 4, 8, 12, 16, 20, 24, 36, 48 and 60)
- Radiological disease assessment (weeks 8, 16, 24, 36, 48, and 60)
- Timepoint 2 translational blood sample (week 8)

Treatment weeks 64+ (assessed until disease progression)

If participants have not progressed by week 60 they can remain on trial therapy from week 64 onwards. Assessment of disease will be according to local PI's practice and trial based monitoring will change to a three monthly review, collecting data on date of progression, SAEs, trial withdrawal or death only:

- Treatment details including medication kit numbers
- Physical examination
- ECOG performance status
- Toxicity assessment
- ECG repeated 3 times
- Clinical disease assessment

End of treatment (assessed when a participant withdraws due to disease progression)

- Physical examination
- Co-morbidities
- Concomitant medication
- Toxicity assessment
- Clinical disease assessment
- Radiological disease assessment

CONFIDENTIAL

This material is the property of the Wales Cancer Trials Unit. Do not disclose or use except as authorised.

- Timepoint 3 translational blood sample

End of study (assessed 30 days after end of treatment)

- Physical examination
- Concomitant medication
- Toxicity assessment

Data will be collected for the following unscheduled events

- Unsechduled additional ECGsWithdrawal
- Death
- SAEs

Endpoints:

Primary outcome measure

Progression-free survival (PFS - time to event) based on Response Evaluation Criteria in Solid Tumours (RECIST) v1.1.

Secondary outcome measures

Safety, tolerability (side effects) and feasibility of use (number of participants requiring dose delays or reductions and/or treatment withdrawal).

Objective response rate and clinical benefit rate as assessed by RECIST v1.1.

Overall survival (OS), time from enrolment to death with those still alive censored at date last seen.

Exploratory analysis: The influence of RET signalling pathway components expression on vandetanib activity.

This material is the property of the Wales Cancer Trials Unit. Do not disclose or use except as authorised.

2.1 Lay summary

For patients with advanced breast cancer (ABC) that has spread around the body, hormone therapy is often the best treatment. As well as being very effective, this means that patients do not experience the toxicity and inconvenience of chemotherapy. However, eventually the cancer is likely to become resistant to hormone therapy and in this situation, although other hormone drugs can be used, they sometimes do not work very well. So it is important to find ways of getting the cancer to respond to hormone treatment again.

There are many different ways in which breast cancer cells become resistant to hormone treatments, including a 'signalling' pathway in the cells called RET. Research has shown increased activity of RET signalling pathways in hormone resistant cancer cells.

Vandetanib is an oral drug that inhibits RET signalling in cells and has been shown in laboratory studies to prevent the growth of breast cancer cells which have become resistant to hormone therapy. Hormone therapy drugs include tamoxifen, and the aromatase inhibitors (anastrozole, letrozole and exemestane). We therefore believe that giving vandetanib together with hormone therapy may help prevent resistance to treatment in patients with breast cancer. In this trial we will combine this drug with fulvestrant, another hormone therapy drug which is sometimes used alone in patients who have developed resistance to aromatase inhibitors, or tamoxifen. So patients entering the trial will have one drug, fulvestrant, which is known to work and may also be given the experimental drug, vandetanib.

To properly determine if vandetanib works as we believe, this study will compare the activity of vandetanib combined with fulvestrant with fulvestrant combined with an inactive, 'placebo' tablet in a group of patients for whom treatment with single agent fulvestrant is thought appropriate. We plan to recruit a total of 160 patients. Half of them will be given fulvestrant and vandetanib and half will be given fulvestrant and placebo, and the treatment a particular patient will get will be chosen by random chance. Neither the patient nor her doctor will know whether she is getting vandetanib or the inactive placebo. The most important measure of effect will be the time until the cancer grows again, but the study will also look closely at the side effects of the drugs. We will also look at whether the way in which an individual responds relates to the results laboratory studies on the RET pathways carried out on previously stored tumour samples. This will mean that patients will not need to have additional biopsy samples taken.

This material is the property of the Wales Cancer Trials Unit. Do not disclose or use except as authorised.

3.0 Background, rationale and objectives

Estrogen Receptor Positive Metastatic Breast Cancer

In 2010, 49,564 women in the UK were diagnosed with invasive breast cancer and 11,556 died [1]. The majority of early breast cancers are estrogen-receptor positive breast cancer (ER+ve) and women with such tumours receive adjuvant endocrine therapy. Such endocrine therapy will cure approximately 30% of women with undetected micro-metastatic disease but 70% will relapse and subsequently die from ER+ve endocrine resistant metastatic breast cancer (MBC) [2] . In ER+ve ABC, endocrine therapy is the treatment of choice due to its improved toxicity profile and comparable efficacy when compared with cytotoxic chemotherapy. However, half of such cancers will progress through first line therapy (primary endocrine resistance) and half will progress after an initial period of disease control (secondary or acquired endocrine resistance). There is a significant need to improve upon current endocrine therapies by circumventing these resistance mechanisms.

Endocrine Therapy for ER+ve Advanced Breast Cancer

Aromatase inhibitors (AIs) have, until recently, been the treatment of choice for de novo metastatic disease as well as for patients treated with adjuvant tamoxifen [3]. Increasingly, however, AIs are also being used in the adjuvant setting and the most efficacious sequence of endocrine therapies in this situation has not been defined. Fulvestrant at a dose of 250mg q28 days was shown to be as effective as the steroidal AI exemestane following failure of a non-steroidal AI (anastrazole or letrozole) in postmenopausal women with metastatic breast cancer [4]. Subsequently fulvestrant at a dose of 500mg q28 days with an extra loading dose on day 15 was shown to be superior to the 250mg dose given according to the same schedule and is currently licenced at the higher dose [5] .

The use of third-generation aromatase inhibitors in endocrine-naive advanced disease has recently been challenged. In the FIRST study, fulvestrant at the 500mg dose was shown to be more effective than anastrazole [6]. Fulvestrant treatment resulted in longer progression-free survival (PFS) than anastrazole (23.4 months versus 13.1 months, HR 0.66; 95% CI 0.47–0.92, P<0.01) despite no improvement in clinical benefit rate [72.5% versus 67.0%, respectively (odds ratio, 1.30; 95% CI, 0.72–2.38; P=0.386)]. A randomised phase III trial in the same patient population is currently recruiting. Fulvestrant is therefore a very reasonable choice of drug for the patient group under investigation in this study.

Rationale for vandetanib in breast cancer

Vandetanib is an oral inhibitor of vascular endothelial growth factor receptor 2 (VEGFR-2), epidermal growth factor receptor (EGFR) and RET tyrosine kinases. Multiple potential mechanisms of endocrine resistance in breast cancer have been defined pre-clinically [7] and include epidermal growth factor receptor (EGFR) and Glial Cell Line-Derived Neurotrophic Factor Receptor (GFR)-coupled RET signalling. The importance of the RET signalling pathway has become increasingly evident from emerging data. For example microarray studies have shown increased activity of RET and GFR 3 signalling pathways in hormone resistant cell lines [8]. siRNA studies have shown that down regulation of either GFR 1 or GFR 3 RET signalling leads to inhibition of growth in hormone resistant cell lines. In addition RET and GFR 1 were detected in 29.7% and 59.4% of breast cancers, preferentially in ER positive specimens [9, 10].

CONFIDENTIAL
This material is the property of the Wales Cancer Trials Unit. Do not disclose or use except as authorised.

Other studies indicate that RET levels are increased in hormone resistant breast cancer specimens. More recently, in-vitro work with vandetanib has shown it to be superior to Gefitinib (an EGFR inhibitor) in producing sustained inhibition of endocrine resistant cell lines (Julia Gee personal communication). Together these data present a strong rationale for the use of vandetanib in patients with endocrine therapy resistant breast cancer.

Selection of 300mg dose for Vandetanib

Pre-clinical data

A number of studies have looked at the activity of vandetanib in mouse models. Gustafson *et al* [11] showed that the Cmax derived from a dose of 25mg per kg p.o. in the mouse approximates to the steady state levels achieved in man at the 300mg once daily dose. Preclinical data from a number of xenograft models [12] show a clear dose response from 12.5mg per kg to 100mg per kg p.o. with the incidence of tumour regression increasing between 50 and 100mg per kg doses. Vitagliano *et al* [13] reported on the activity of vandetanib in preclinical models of MTC, using MTC cell lines TT and MZ-CRC1 that carry C634W and M918T activating RET mutations respectively. Vandetanib inhibited proliferation and RET EGFR and KDR phosphorylation in both cell lines in vitro at concentrations that would be achievable with clinical doses. Supplementary data in the same paper showed that inhibition of RET phosphorylation in TT xenografts accompanied inhibition of tumour growth following treatment with 50mg per kg vandetanib.

Clinical information

Data from a number of trials have shown that a daily dose of 300mg vandetanib is tolerable and optimal for efficacy (see Investigator Brochure (IB) for details).

In breast cancer there have been two randomised phase II studies published (one in abstract form only). A study by Boer *et al* [14] examined the efficacy and safety of 100mg vandetanib with docetaxel as second-line treatment for advanced breast cancer. The addition of vandetanib to docetaxel did not affect the risk of disease progression compared with placebo plus docetaxel. The safety and tolerability profile of the combination therapy reflected those of both drugs as monotherapy agents. However the trial had small patient numbers (64 in total) and was not sufficiently powered to observe differences. The ZAMBONEY trial [15] randomised postmenopausal women with bone only or predominantly bone metastases to fulvestrant + 100 mg vandetanib or fulvestrant + placebo. There was no observed difference between the arms in the primary outcome measure - a decrease in urinary N-telopeptide (NTx) bone markers. So there are currently no clinical data suggesting a 100mg dose of vandetanib has significant activity in breast cancer patients. In addition neither of these studies performed a subgroup analysis according to RET pathway activation.

Given the combination pre-clinical efficacy data and clinical/safety data, a dose of 300mg od vandetanib has been selected for this study.

Pharmacokinetics

CONFIDENTIAL
This material is the property of the Wales Cancer Trials Unit. Do not disclose or use except as authorised.

No specific interactions are expected between fulvestrant and vandetanib. Further details are available in the IBs for both drugs.

Research hypotheses

The addition of vandetanib to fulvestrant will be more efficacious than fulvestrant alone - as demonstrated by an increase in PFS in patients with ER+ve advanced or metastatic breast cancer.

Breast cancers that have activation of RET signalling will have increased sensitivity to vandetanib and, therefore, demonstrate greater improvement in PFS to vandetanib when combined with fulvestrant than those without such molecular aberrations.

Study Objectives

To establish whether the combination of vandetanib and fulvestrant will improve clinical outcome in patients with endocrine resistant advanced breast cancer (RECIST v1.1 Appendix 1) compared to fulvestrant alone.

To determine whether the combination of vandetanib and fulvestrant is tolerable, safe and feasible to deliver.

To determine whether vandetanib activity is related to the activation status of the tumour RET signalling pathway.

CONFIDENTIAL

This material is the property of the Wales Cancer Trials Unit. Do not disclose or use except as authorised.

4.0 Study design

This is a double-blind, randomised, placebo controlled, two arm phase II trial. Eligible patients are post-menopausal women with locally advanced or metastatic ER+ve breast cancer not suitable for surgical resection. Patients should be suitable for endocrine treatment, but have received no more than three previous lines of endocrine treatment and up to one line of chemotherapy for ABC. They will also have had progressive disease during treatment with a third generation aromatase inhibitor or have relapsed on an AI in the adjuvant setting.

The participant's research nurse and/or doctor will screen the participant to ensure that they meet the trial eligibility criteria, before obtaining the participant's consent. The research nurse will then randomise the patient to either vandetanib or placebo through the electronic interactive web response system (IWRS). The IWRS system will require confirmation of the eligibility criteria before allocating the treatment and providing the allocated trial participant number to the research nurse or doctor. Each centre will be provided with a user guide and login details for the IWRS.

Participants will receive fulvestrant in combination with either placebo or vandetanib until disease progression. Each cycle will be 28 days in duration. Randomisation will have a 1:1 allocation and use the method of minimisation (with a random element) to ensure treatment arms are balanced on important stratification criteria including:

- Resistance to AI therapy
- Measurable vs non-measurable disease

Primary resistance is defined as either 1) disease relapse during or within 6 months (i.e., 26 weeks) of completing AI treatment in the adjuvant setting, or 2) disease progression within 6 months of starting AI treatment and no response to AI treatment in the metastatic setting. Secondary resistance is defined as stable disease (SD) for a minimum of 6 months, or complete response (CR) or partial response (PR) to prior metastatic AI treatment.

The archival tissue sample will be collected from each patient to perform a retrospective exploratory analysis looking at the association of vandetanib activity with expression of key components of the RET signalling pathway in primary specimens.

Safety review

Two safety reviews will be performed after 20 and 40 participants have received at least 1 cycle of treatment. The WCTU will request that all toxicity forms and any serious adverse events (SAEs) are submitted promptly following the first cycle. Recruitment will continue as normal during these reviews.

This material is the property of the Wales Cancer Trials Unit. Do not disclose or use except as authorised.

Completion of Treatment

Participants in both stages of the trial may continue vandetanib/placebo until disease progression or until intolerable treatment toxicity or consent withdrawal. It is estimated that participants will complete trial treatment by week 64; however participants may be treated beyond this if they have not yet progressed (See Section 8.1.5 Drug supply, distribution and storage).

Assessment of disease will then be according to local PI practice and the research nurse will be required to complete 3-monthly trial assessment forms to record data on date of progression, SAEs, trial withdrawal or death. These assessments will be required until the trial is closed.

4.1 Risk assessment

A Trial Risk Assessment has been completed by the WCTU to identify the potential hazards associated with the trial and to assess the likelihood of those hazards occurring and resulting in harm. This risk assessment has been completed in accordance with the MRC/DH/MHRA Joint project guidance document 'Risk-adapted approaches to the management of Clinical Trials of Investigational Medicinal Products' and includes:

- The risk to participant safety in relation to the IMP.
- All other risks related to the design and methods of the trial (including risks to participant safety and rights as well as data integrity).
- The potential risks have been balanced against the level of risk that a trial participant would be exposed to outside of the trial. This trial has been categorised as a TYPE B, where the level of risk is somewhat higher than the risk of standard medical care. A copy of the trial risk assessment may be requested from the WCTU Trial Manager. The trial risk assessment is used to determine the intensity and focus of monitoring activity (see section 11.2).

CONFIDENTIAL

This material is the property of the Wales Cancer Trials Unit. Do not disclose or use except as authorised.

5.0 Participating site selection.

This study will be carried out at participating sites within the UK. All sites who are interested in participating in the trial will be required to complete a registration form to confirm that they have adequate resources and experience to conduct the trial.

The following documentation must be completed and received by the WCTU in order for a site to begin recruitment:

- Confirmation of local R&D approval.
- Favourable opinion of host care organisation/PI from Main Ethics committee.
- Signed Model Agreement for non-commercial research between the host care organisation and Sponsor.
- Signed Material Transfer Agreement between the host care organisation and Sponsor.
- Current Curriculum Vitae and GCP training certificate of the Principal Investigator (PI).
- A copy of the most recent approved version of the Participant Information Sheet(s) and Consent Form(s) on host care organisation headed paper.
- A copy of the most recent approved GP letter on host care organisation headed paper.
- A copy of the most recent Pregnancy Information Sheet(s) and Consent Form(s) on host care organisation headed paper.
- Completed Delegation Log (signature list and delegation of responsibilities).
- Full contact details for all personnel from the host organisation involved, indicating preferred contact.
- A set of laboratory normal ranges and laboratory certification/accreditation from the host care organisation laboratory being used for analyses.
- Completed Self-Evident Correction Log.
- Confirmation that site has access to the IWRS system including the unblinding forms.

Once all the documentation has been received at the WCTU, confirmation of site approval will be sent by the WCTU to the site PI and the Clinical Supplies department of AstraZeneca who are providing the vandetanib and fulvestrant.

All documentation must be stored in the Investigator Site File (ISF) at the site and in the Trial Site File (TSF) at the WCTU. The WCTU must be notified of any changes in the trial personnel and their responsibilities during the running of the trial and the respective trial files must contain this up-to-date information.

Occasionally during the trial, amendments may be made to the trial documentation listed above. WCTU will issue the site with the latest version of the documents as soon as they become available. It is the responsibility of the site to ensure that they obtain local R&D approval for the new documents, and that

This material is the property of the Wales Cancer Trials Unit. Do not disclose or use except as authorised.

all relevant staff, including pharmacy staff, are working to the current versions once R&D approval has been obtained.

Site initiation will be by by teleconference.

This material is the property of the Wales Cancer Trials Unit. Do not disclose or use except as authorised.

6.0 Participant eligibility

Any queries about whether a patient is eligible to enter the trial should be discussed with the WCTU before randomisation. Any issues will then be raised with the CI or one of the clinical Co-Investigators in the CI's absence.

Patients are eligible for the trial if all the inclusion criteria (Section 6.2) are met and none of the exclusion criteria (Section 6.3) apply:

6.1 Screening procedures

Before any trial related procedures are undertaken, the patient's written informed consent must be obtained. The patient should be given a minimum of 24 hours after initial invitation to participate before being asked to sign the consent form.

Screening logs should be completed for every patient considered for the trial. Copies of these screening logs should be sent to the WCTU upon request.

The screening assessments outlined in Section 9.1 of this Protocol should be performed within 28 days before inclusion in the study to confirm eligibility.

6.2 Inclusion criteria

Patients meeting all of the following criteria may be included in the trial:

1. Adult female patients, aged ≥ 18 years.
2. Post-menopausal patients. Post-menopausal can be defined as either of the following criteria:
   a. Amenorrhoeic throughout AND after therapy with a third generation AI, without a GnRH analogue (eg. goserelin) AND screening FSH and estradiol in institutional post-menopausal ranges.

   OR

   b. Treatment of early or metastatic breast cancer with a third generation AI and GnRH analogue, with discontinuation of the GnRH analogue for at least 6 months AND no resumption of menstruation AND screening FSH and estradiol in institutional post-menopausal ranges.
3. Patients that do not meet these criteria but are considered post-menopausal due to additional clinical evidence may be considered eligible at the local PIs discretion. Patients that have had a hysterectomy are eligible providing both ovaries have been surgically removed. Minimum life expectancy of 12 weeks.
4. Histological confirmation of ER+ve breast cancer on primary tumour at diagnosis or on biopsy of a metastasis. ER is considered positive if ≥10% of tumour cells stain positive for ER (whatever the intensity of staining). If no percentage score is available then a Quick (Allred) Score of ≥4/8 or H Score of >150/300 (i.e. 50% positive cells) will be considered ER positive.

This material is the property of the Wales Cancer Trials Unit. Do not disclose or use except as authorised.

5. Histological confirmation of HER2 negative breast cancer on primary tumour at diagnosis or on biopsy of a metastasis. HER2 is considered negative by IHC if scored 0 or 1+ by Herceptest or similar assay. If HER2 is scored 2+ or 2+/3+ by IHC then HER2 gene amplification must be assessed by FISH/CISH/D-DISH and the ratio of HER2 to CEP17 probes must be <2.0.
6. Clinical or histological confirmation of metastatic or locally advanced disease not amenable to curative surgical resection.
7. ECOG performance status 0 to 2 with no deterioration over the previous 2 weeks.
8. Measurable or non-measurable disease.
9. Patient has adequate bone marrow and organ function as defined by the following:
    a. Absolute Neutrophil Count (ANC) ≥ 1.0 x 10^9/L.
    b. Platelets (plts) ≥ 100 x 10^9/L.
    c. Haemoglobin (Hgb) ≥ 9 g/dl [Note: any blood transfusion must be >14 days prior to the determination of haemoglobin].
    d. Prothrombin time (seconds) ≤ 1.5 x ULN.
    e. Potassium, calcium (corrected for serum albumin) and magnesium within normal limits (WNL) for the institution.
    f. Serum creatinine ≤ 1.5xULN.
    g. Alanine aminotransferase (ALT) or aspartate aminotransferase (AST) ≤ 2.5xULN (or < 5.0 x ULN if liver metastases are present).
    h. Total bilirubin ≤1.5 times ULN.
    i. Alkaline Phosphatase (ALP) <5XULN.
10. Progressive disease whilst receiving a third generation aromatase inhibitor (exemestane, anastrazole or letrozole) for locally advanced or metastatic BC or relapsed with metastatic disease whilst receiving a third generation AI in the adjuvant setting. The AI does not need to be the last treatment immediately prior to recruitment.
11. Radiological or objective clinical evidence of recurrence or progression on or after the last systemic therapy prior to enrolment.
12. Up to 3 prior lines of endocrine therapy for ABC (both incurable locally advanced or metastatic breast cancer). If an attempt to downstage a locally advanced tumour with endocrine therapy was made in the absence of MBC, and the tumour operated upon, then this does not count as a line of therapy for ABC. In contrast, if the tumour remained inoperable then this treatment should be included as a line of therapy for ABC.
13. No more than 1 line of cytotoxic chemotherapy for ABC (see inclusion criterion 12 for note on definition of lines of therapy).
14. Suitable for further endocrine therapy according to the treating clinician.
15. Availability of archival tumour sample or fresh biopsy for exploratory analysis.
16. Provision of informed consent prior to any study specific procedures.

CONFIDENTIAL

This material is the property of the Wales Cancer Trials Unit. Do not disclose or use except as authorised.

6.3 Exclusion criteria

If any of the following criteria apply, patients cannot be included in the trial:

1. Previous treatment with fulvestrant or inhibitors of the RET pathway.
2. Last dose chemotherapy, immunotherapy targeted therapy, biological therapy or tumour embolisation less than 21 days (less than 6 weeks for nitrosurea or mitomycin C) prior to the first dose of study treatment. *Note: endocrine (hormone) therapy is not considered a targeted or biological therapy and does not require a wash out period for the purposes of this study. Denosumab and bisphosphonate treatment are accepted concomitant medications as long as they are started at least 14 days prior to study drug commencement.*
3. Last dose of palliative radiotherapy less than 7 days prior to the first dose of study treatment.
4. Rapidly progressive visceral disease not suitable for further endocrine therapy.
5. Spinal cord compression or brain/meningeal metastases unless asymptomatic, treated and stable and not requiring steroids for at least 4 weeks before starting study treatment.
6. Clinical evidence of abnormal cardiac function or significant hypertension as defined by one or more of the following criteria:
    a. Significant cardiac event (e.g., myocardial infarction), superior vena cava syndrome, New York Heart Association (NYHA) classification of heart disease ≥2 within 12 weeks before randomisation (see Appendix 2), or presence of cardiac disease that in the opinion of the Investigator increases the risk of ventricular arrhythmia.
    b. History of arrhythmia (multifocal premature ventricular contractions, bigeminy, trigeminy, ventricular tachycardia), which is symptomatic or requires treatment (CTCAE v 4.03 Grade 3), symptomatic or uncontrolled atrial fibrillation despite treatment, or asymptomatic sustained ventricular tachycardia. Patients with atrial fibrillation controlled by medication are permitted.
    c. Congenital long QT syndrome.
    d. History of QT prolongation associated with other medications that required discontinuation of that medication
    e. QTc >480msec on screening ECG. (Note: The screening ECG must be repeated three times 5 minutes apart. *The average QTc from the three screening ECGs must be ≤480 ms in order for the patient to be eligible for the study. If the average QTc is >480ms, the ECGs may be repeated at least 24 hours later, and the average must be ≤480 ms.* If a patient is receiving a medication that has a risk of Torsades de Pointes, and cannot be discontinued before study entry, refer to Section 8.1.4 Concomitant medications/procedures of this Protocol for additional guidance on eligible screening QTc values and additional ECG screening requirements whilst on treatment.)
7. Patients with the following electrolyte values (the rational is due to the increased risk of prolonged QTc):
    a) Potassium <4.0 mmol/L despite supplementation, or above the CTCAE Grade 1 upper limit, at the time of randomisation. A patient may have a serum potassium level of <4.0mmol/L and continue through screening providing they receive potassium supplements. If, despite

This material is the property of the Wales Cancer Trials Unit. Do not disclose or use except as authorised.

this supplementation, their serum potassium remains <4.0mmol/L prior to randomisation then they will be ineligible for study entry.

b) Magnesium below the normal range despite supplementation, or above the CTCAE Grade 1 upper limit, at the time of randomisation.

c) Calcium (ionised or serum) below the normal range despite supplementation, or above the Grade 1 upper limit, at the time of randomisation. If serum calcium is used, correction should be applied to account for hypoalbuminemia, if present, where the corrected serum calcium (mmol/L) is equal to measured serum Ca (mmol/L) + 0.8 x (4 - serum albumin g/L).

8. Creatinine clearance <30 ml/min (calculated by Cockcroft-Gault formula, see Appendix 4). Patients with creatinine clearance <50 µmol/min will start at a permanently reduced vandetanib dose of 200 mg.
9. Major surgery (excluding placement of vascular access) within 4 weeks before the first dose of study treatment.
10. As judged by the Investigator, any evidence of severe or uncontrolled systemic diseases, including uncontrolled hypertension, active bleeding diatheses, or active infection including hepatitis B, hepatitis C and human immunodeficiency virus (HIV). Screening for chronic conditions is not required.
11. With the exception of alopecia, any unresolved toxicities from previous therapy greater than CTCAE grade 1 at the time of starting study treatment.
12. Elevated ALP in the absence of bone metastasis. *If the patient has elevated (ALP) in the presence of bone metastasis and liver function is otherwise considered adequate in the investigator's judgement, then the patient is not exluded.*
13. History of hypersensitivity to active or inactive excipients of vandetanib or fulvestrant.
14. Evidence of dementia, altered mental status or any psychiatric condition that would prohibit understanding or rendering of informed consent.
15. Participation in another clinical study with an investigational product (IP) during the last 30 days.
16. Inability or unwillingness to comply with study procedures, including the inability to take regular oral medication.
17. Previous or concomitant malignancies at any other site with the exception of the following:
    a. Benign basal cell carcinoma.
    b. Benign low grade transitional cell carcinoma of the bladder.
    c. Other effectively treated malignancy that has been in remission for more than 5 years and is considered to be cured.

6.4 Restrictions

1. The concomitant use of known potent CYP3A4 inducers (e.g. barbiturates, rifampicin, phenytoin, carbamazepine, troglitazone, St. John's Wort) must be avoided 2 weeks before and for the duration of the study (dexamethasone (or equivalent) may be given as a pre-medication for chemotherapy). A non-definitive list of CYP3A4 inducers may be found at https://liferaftgroup.org/long-list-of-inhibitors-and-inducers-of-cyp3a4-and-cyp2d6/.

CONFIDENTIAL
This material is the property of the Wales Cancer Trials Unit. Do not disclose or use except as authorised.

2. Concomitant use of medications with a known risk of Torsade de Pointes should only be used when the benefits far outweigh the risks. Alternative medications should be considered and if not possible, increased ECG monitoring may be undertaken (i.e. ECG prior to use of the new medication, at its steady state and as clinically indicated afterwards). For a composite list of all drugs that prolong QT and/or cause Torsades de Pointes please see https://crediblemeds.org/pdftemp/pdf/CombinedList.pdfand for drugs to be avoided by congenital long QT patients please see: http://crediblemeds.org/pdftemp/pdf/DrugsToAvoidList.pdf).
3. Patients should not take any additional medication without the prior consent of the investigator other than those listed under "permitted medications" in Section 8.1.4 Concomitant medications/procedures.
4. Patients with hypertension (elevated BP) associated to white coat syndrome remain eligible for study entry providing the ECG is normal with no arrhythmias or QTc prolongation. Blood pressure should be monitored regularly and treated if it remains raised.
5. Patients with lactose intolerance remain eligible providing the patient is well nourished and not malabsorbing.
6. To avoid cutaneous toxicity, it is strongly recommended that all patients follow a program of sun protective measures (wearing additional clothing and/or SPF50 sunscreen) while receiving study treatment and for 3 to 4 weeks after discontinuing study treatment. The aim is to reduce the risk of development of skin rash, or minimise the severity of skin rash, and to minimise the requirement for dose reduction of study therapy. If a patient develops a skin rash, the following actions are recommended to the Investigator for the management of this reaction:

    - A variety of agents can be used to manage skin rashes. These include mild to moderate strength steroid creams or systemic glucocorticoids, either topical or systemic antibiotics, topical or systemic antihistamines, and occasionally retinoid creams.
    - The rash should be graded as soon as possible according to the CTCAE cutaneous toxicity criteria.
    - If a rash of CTCAE Grade 2 or higher is detected, immediate symptomatic treatment should be provided.
    - If a rash of CTCAE Grade 3 or 4 is detected, vandetanib should be withheld until recovery to CTCAE Grade 1 or baseline; vandetanib can then be restarted at a permanently reduced dose, see section 8.1.2 and table 1.

6.5 Informed consent

The patient's written informed consent must be obtained using the FURVA trial Consent Form, which follows the PIS. The patient should be given a minimum of 24 hours after the initial invitation to participate before being asked to sign the Consent Form. Please note, only when written informed consent has been obtained from the patient and they have been randomised/enrolled into the trial can they be considered a trial participant.

This material is the property of the Wales Cancer Trials Unit. Do not disclose or use except as authorised.

The patient’s consent to participate in the trial should be obtained by the treating doctor or a suitable qualified delegate (doctor or research nurse) after a full explanation has been given of the treatment options, including the conventional and generally accepted methods of treatment. All patients must be informed of the aims of the study, the possible adverse events, the procedures and possible hazards to which they may be exposed. They will be informed of the strict confidentiality of their patient data, but that their medical records may be reviewed for trial purposes by authorised individuals other than their treating physician.

Patient’s consent will be sought to notify their GP of their involvement in the trial. Patients should be given a minimum of 24 hours after being given the trial Participant Information Sheet to consider and discuss participation in the trial with friends and family. A contact number for someone at the site should be given to the patient should they wish to discuss any aspect of the trial. Following this, the investigator should determine that the patient is fully informed of the trial and their participation, is in accordance with the principles of Good Clinical Practice. Patients should always be asked to sign a consent form. One copy should be given to the participant but the original copy should be kept in the investigator site file and a further copy should be kept with participant’s hospital notes.

Optional participant consent is requested to collect NHS Numbers to use NHS data for future research, through Cancer Research UK and the National Cancer Intelligence Network (NCIN).

The right of the participant to refuse to participate in the trial without giving reasons must be respected. After the patient has entered the trial, the investigator must remain free to give alternative treatment to that specified in the protocol, at any stage, if he/she feels it to be in the best interest of the participant. However, the reason for doing so should be recorded and the participant will remain within the trial for the purpose of follow up and data analysis according to the treatment option to which he/she has been allocated. Similarly, the participant must remain free to withdraw at any time from the protocol treatment without giving reasons and without prejudicing his/her further treatment.
This is a randomised controlled trial; therefore neither the participants nor their physicians will be able to choose the participant’s treatment. Treatment will be allocated randomly using a computer-based algorithm. This is to ensure that the groups of participants receiving each of the different treatments are similar.

This material is the property of the Wales Cancer Trials Unit. Do not disclose or use except as authorised.

7.0 Randomisation

The participant's research nurse and/or doctor will screen the participant to ensure that they meet the trial eligibility criteria, prior to obtaining participant consent. The research nurse will then randomise the patient to either vandetanib or placebo through the patient randomisation function of the electronic interactive web response system (IWRS). The IWRS system will require confirmation of the eligibility criteria before allocating treatment and providing the allocated trial participant number to the research nurse or doctor. Each centre will be provided with a user guide and login details for the IWRS.

Each centre will be provided with packaged IMP and nIMP drugs (see Section 8.1.5 Drug supply, distribution and storage). The IWRS will allocate the IMP/placebo treatment and will provide the randomisation number and assign the appropriate unique IMP/placebo treatment kit number for Cycle 1 of treatment from those available at the centre. The IWRS will also confirm the dispensing of the Cycle 1 Day 1 nIMP fulvestrant. Treatment Cycle 1 Day 15 onwards will be assigned by the centre using the patient dispensing visit function of IWRS following the dosing regime specified in Section 8.0 Trial treatments.

The randomisation is centralised, and the assigned randomisation number and associated IMP/placebo kit numbers will not be sequential within a centre. If a patient is given the incorrect kit, the patient should continue to take the medication they have been allocated. The site must inform the IWRS immediately after the error is identified.

At randomisation, the participant will be given a unique participant trial number which should be recorded on the participant randomisation CRF and the top copy returned to the WCTU within four weeks.

The centre will inform the participant's General Practitioner (GP) of the participant's enrolment in FURVA using the FURVA GP letter, if the participant gives consent to do so.

It may be possible for participants to be recruited into other clinical trials, but this should be discussed with the CI via the WCTU before this is considered.

8.0 Trial treatments

Cycle 1 Day 1 Treatment will commence within 28 days of the date of the screening CT scan if possible, and must be within two weeks of the date of randomisation, i.e. ≤6 weeks from the date of the screening CT scan.

8.1 Fulvestrant and vandetanib/placebo

Fulvestrant

This material is the property of the Wales Cancer Trials Unit. Do not disclose or use except as authorised.

Fulvestrant is considered to be a non-investigational medicinal product (NIMP) in this trial, as it will be used within its marketing authorisation. It is administered slowly as two x 5ml (250mg) intramuscular injections, one into each buttock. Fulvestrant 500 mg will be given on day 1 of every 28 day cycle (± 3 days window). An additional loading dose of 500 mg will also be delivered on Cycle 1 Day 15. Fulvestrant will be administered by a suitably trained health professional at the hospital site. Administration guidance can be found at: http://www.ema.europa.eu.

Vandetanib/placebo

Patients will receive oral vandetanib/placebo tablets once a day by mouth for the duration of the trial, until IMP treatment is discontinued. The starting dose will be 300 or 200 mg dependent on renal impairment status.

There are no regular supportive medications mandated for patients starting the trial. In the event of an AE which the investigator considers to be related to the administration of study treatment (fulvestrant or vandetanib/placebo), supportive therapy should be given at the discretion of the investigator. We recommend that nausea and vomiting are treated according to local policy, as long as drugs are not contraindicated in the restrictions section.

Treatment breaks

Patients may take treatment breaks for a duration of ≤2 weeks for any toxicity that is ≥ Grade 2, or multiple Grade 1 toxicities, at the discretion of the local PI.

Treatment discontinuation

- If fulvestrant is discontinued for reasons other than disease progression, the participant may continue on vandetanib/placebo alone at the investigator’s discretion. The participant must continue being scanned for RECIST 1.1 assessment in accordance with the schedule of assessments until objective disease progression or withdrawal from vandetanib/ placebo treatment.

- If vandetanib /placebo is discontinued for reasons other than disease progression, the participant may continue on fulvestrant alone at the investigator’s discretion. The participant must continue being scanned for RECIST 1.1 assessment in accordance with the schedule of assessments until objective disease progression or withdrawal from fulvestrant.

- In either situation above, and until disease progression, no additional anticancer therapy may be added to single agent vandetanib /placebo or fulvestrant to replace the discontinued therapy.

- If a participant becomes amenable for surgery to remove the primary tumour or a metastasis, surgery the decision as to whether to continue treatment with fulvestrant/ vandetanib / placebo should be discussed with the Chief Investigators. The participant must continue being scanned for RECIST 1.1 assessment post-surgery until objective disease progression.

8.1.1 Scheduling

This material is the property of the Wales Cancer Trials Unit. Do not disclose or use except as authorised.

Vandetanib/Placebo
Vandetanib/placebo tablets must be swallowed whole with water. For patients who have difficulty swallowing, vandetanib tablets may be dispersed in half a glass of non-carbonated drinking water. No other liquids should be used. The tablet is to be dropped in water, without crushing, stirred until dispersed (approximately 10 minutes) and the resultant dispersion swallowed immediately. Any residues in the glass are to be mixed with half a glass of water and swallowed.

Patients should be instructed to take their medication at approximately the same time each day with or without food. . If the patient inadvertently does not take the dose in the morning, she may take that day's dose any time up to 10 p.m. that same day. However, if she misses taking the scheduled dose and is unable to take the missed dose on the same day, she must take the next scheduled dose and the missed dose will not be made up. The missed dose must be documented on the appropriate CRF at the next hospital appointment. The dose of study treatment may be repeated if vomiting occurs within 30 minutes of taking the study treatment. If vomiting occurs after 30 minutes of taking the study treatment, the dose will not be repeated.

Fulvestrant
Fulvestrant injections may be administered either before or after the patient has taken their vandetanib/placebo tablets following local practice.

8.1.2 Dose modifications

Vandetanib dose modification for hypertension
Patients who develop CTCAE grade 3 hypertension may continue on vandetanib therapy if blood pressure is controlled on antihypertensive medication. If blood pressure cannot be stabilised with increased antihypertensive medication, vandetanib must be withheld and cannot be resumed until blood pressure is controlled to baseline level. Vandetanib should also be withheld for patients with CTCAE grade 4 hypertension and therapy cannot resume until blood pressure is controlled to baseline level. Following the interruption for hypertension patients should resume treatment at a permanently reduced dose of 200mg. If vandetanib must be interrupted for more than 3 weeks to allow for toxicity to resolve, the patient's study participation will be discontinued. If a patient develops high blood pressure suspected to be indicative of white coat syndrome the patient may remain on treatment without dose modification providing they are referred to their GP for their blood pressure to be taken over three separate readings. The resulting data should be recorded in the patients hospital notes and provided to the WCTU upon request.

Vandetanib dose modifications for renal toxicity
Patients with creatinine clearance <30 ml/min (calculated by Cockcroft-Gault formula) are excluded from the study. Patients with creatinine clearance <50 mL/min should start at a permanently reduced vandetanib dose of 200 mg. During treatment, if creatinine clearance falls to <50 mL/min, then reduce the dose by one dose level. Reduce by a further dose level if it drops below <40 mL/min and discontinue vandetanib if it drops below 30 ml/min.

CONFIDENTIAL
This material is the property of the Wales Cancer Trials Unit. Do not disclose or use except as authorised.

Vandetanib dose modification for hepatic impairment
Patients with grade 3 hepatic dysfunction should stop vandetanib until toxicity resolves to grade 1 or below and resume dosing as described for other toxicities below.

Vandetanib dose modifications for other toxicities
Any grade 3 or 4 toxicity which, in the opinion of the investigator, is due to vandetanib: withhold until toxicity resolves to grade 1 or below. Resume dosing at the lower dose of 200mg od. If grade 3 or 4 toxicity recurs at the reduced rate, withhold until toxicity resolves to grade 1 or below, then resume dosing at the lower dose of 100mg. If grade 3 or 4 toxicity recurs at this reduced rate, withhold until toxicity resolves to grade 1 or below, then resume dosing at the lower dose of 100mg every other day.

Table 1 – Vandetanib dose reductions

Treatment regimens (full dose and reduced dose)

	**Patients with no renal impairment**	**Patients with renal impairment (creatinine clearance 30-50 ml/min)**
Full dose	300 mg daily	200 mg daily
Reduced dose	200 mg daily	100 mg daily
Reduced dose	100 mg daily	100 mg every other day
Reduced dose	100 mg every other day	Not available

If grade 3 or 4 toxicity recurs at the most reduced dose, the patient should permanently discontinue vandetanib / placebo.

If vandetanib / placebo is withheld for > 3 weeks, then the patient should be permanently discontinued.

Grade 2 toxicities should be managed symptomatically, see table 2 overleaf.

This material is the property of the Wales Cancer Trials Unit. Do not disclose or use except as authorised.

Table 2 Summary of Guidance on the Management of Toxicity for Vandetanib.

Toxicity	Vandetanib
QTc value ≥500 msec or prolonged ≥60 msec from baseline	Withhold dose.<br>Triplicate ECGs will be obtained at least once per week (done at the same day each week) along with electrolytes, until the average QTc falls ≤480 ms. Study drug treatment may be resumed at a lower dose permanently, after the average QTc returns to ≤480 ms.<br>If study drug (at the reduced dose) is restarted after the QTc prolongation has resolved, ECGs and electrolytes (including calcium and magnesium) must be obtained at 3, 8, and 12 weeks following the start of the lower dose. Serum potassium levels should be maintained at 4 mEq/L or higher, and serum magnesium and serum calcium should be kept within normal range to reduce the risk of QT prolongation. ECG and electrolyte monitoring can then resume at every 12 weeks at the normal visit schedule for the patient.
Emesis within 30 mins of taking vandetanib	Repeat dose.
Gastrointestinal toxicity	Nausea, vomiting, or both may be controlled with anti-emetic therapy. The use of somatostatin or a somatostatin analogue is allowed to control diarrhoea.<br>Diarrhoea should be treated with standard medications to avoid dose modification or interruption, if possible. No dose modifications will be made for Grade 1 or 2 diarrhoea, however, electrolyte supplementation with regular laboratory monitoring should be used, when appropriate, to maintain electrolytes within normal limits and prevent an increased risk of QTc prolongation.If CTCAE Grade 3 or 4 diarrhoea develops, vandetanib should be withheld until diarrhoea resolves to CTCAE Grade 1 or baseline; vandetanib can then be restarted at a permanently reduced dose. Any electrolyte imbalance must be promptly corrected since hypokalaemia and hypomagnesaemia are potential risk factors for drug-induced arrhythmia.
Cutaneous toxicity	If a patient develops a skin rash, the following actions are recommended to the Investigator for the management of this reaction:<br>• A variety of agents can be used to manage skin rashes. These include mild to moderate strength steroid creams or systemic glucocorticoids, either topical or systemic antibiotics, topical or systemic antihistamines, and occasionally retinoid creams.<br>• The rash should be graded as soon as possible according to the CTCAE cutaneous toxicity criteria.<br>• If a rash of CTCAE Grade 2 or higher is detected, immediate symptomatic treatment should be provided.<br>• If a rash of CTCAE Grade 3 or 4 is detected, vandetanib should be withheld until recovery to CTCAE Grade 1 or baseline; vandetanib can then be restarted at a permanently reduced dose.

This material is the property of the Wales Cancer Trials Unit. Do not disclose or use except as authorised.

Toxicity	Vandetanib
Other grade 3 or 4 toxicity related to vandetanib	Withhold dose until toxicity has resolved to CTCAE grade 1 or less, then reduce dose to 200mg. If grade 3 or 4 toxicity recurs, withhold until toxicity has resolved to grade 1 or below, then reduce dose to 100mg. If grade 3 or 4 toxicity recurs at this reduced rate, withhold until toxicity resolves to grade 1 or below, then reduce dose to 100mg every other day.

Fulvestrant dose modifications

The most commonly reported side effects of fulvestrant (10 or more patients out of 100) are injection site reactions, asthenia, elevated liver enzymes (ALT, AST, ALP), and nausea.

Less common side effects (less than 10 out of every 100 patients) include: elevated bilirubin, vomiting, diarrhoea, hot flushes, headache, anorexia, rash, urinary tract infection (UTI) and hypersensitivity reactions.

If an investigator feels that unacceptable toxicity can reasonably be attributed to fulvestrant, or if there are physical difficulties with administration of bilateral injections, a single dose reduction to the previously licensed dose of 250mg every 28 days may be allowed. However, this should first be discussed with the Chief Investigators as fulvestrant 250mg has been shown in randomised trials to be inferior to the 500mg dose.

Breaks in treatment

Planned breaks in the trial treatment schedule should be avoided where possible. If there is a planned break it should be recorded as a dose delay and the treatment specified on the treatment CRF, If a fulvestrant dose is delayed then the next fulvestrant dose should be brought back in line with the scheduled visit dates weher possible.

Treatment discontinuation

Permanent discontinuation of IMP/placebo and/or nIMP should be recorded on the Withdrawal CRF (see Section 9.7 Unscheduled assessments) and Section 11.3 Participant Withdrawal). Permanent discontinuation of IMP/placebo and nIMP should be recorded using the IWRS patient status change function.

8.1.3 Measures of compliance/adherence

Patients will be asked to complete a patient diary card and also to return un-used medications to the site and these will be counted as a measure of compliance.

8.1.4 Concomitant medications/procedures

Prohibited medications /procedures

Inducers of CYP3A4

This material is the property of the Wales Cancer Trials Unit. Do not disclose or use except as authorised.

Concomitant use of the known potent inducers of CYP3A4: rifampicin, phenytoin, carbamazepine, barbiturates and St. John's Wort are not allowed within 2 weeks of study or during the study. Until the effect of CYP3A4 inducers in the exposure to vandetanib in humans has been assessed, the co-administration of such inducers with vandetanib is not allowed.

GnRH analogues

Concomitant use of GnRH analogue (e.g. goserelin , Zoladex) must have been discontinued for ≥6 months with no resumption of mensus prior to trial entry and must not be commenced after trial entry.

Anti-cancer drugs

Treatment with any other anti-cancer drug or experimental agent is not permitted.

Medications permitted with caution

The following medications can be taken by patients, but require additional monitoring:

Warfarin

Warfarin is allowed in therapeutic and low-doses and these patients should be monitored regularly for changes in their International Normalised Ratio (INR), at the discretion of the investigator. If screening blood test results (e..g PT, INR) are outside of the eligible range due to treatment with warfarin, patients may be switched to heparin and allowed a wash out period of a few days prior to reassessment to ensure eligibility prior to study entry.

Drugs assocatied with Torsades de Pointes

Co-administration of drugs associated with Torsades de Pointes should be avoided if possible. These drugs are listed at http://crediblemeds.org/pdftemp/pdf/CompositeList.pdf. However, these drugs will be allowed, at the discretion of the investigator, if considered absolutely necessary. In such cases, the patient must be closely monitored including regular checks of QTc and electrolytes. If a patient is receiving one of the medications that has a risk of Torsades de Pointes, and cannot be discontinued before study entry, then the screening QTc must be <460 msec, and an additional ECG must be obtained 4-8 hours after the first dose of vandetanib. For patients who start on one of the drugs in this group while on the study, the ECG must be checked within 24 hours of commencing the concomitant medication and then at least once per week while the patient remains on the medication. The frequency of ECG monitoring could revert to the standard schedule if no QTc prolongation has been noted during 4 weeks of co-administration of such a drug. Electrolytes should be maintained within the normal range using supplements if necessary.

This material is the property of the Wales Cancer Trials Unit. Do not disclose or use except as authorised.

Metaformin

Concomitant administration of metformin and vandetanib may increase exposure to metformin. Careful monitoring of blood glucose and metformin toxicity is therefore recommended in patients who require metformin.

Digoxin

Concomitant administration of digoxin and vandetanib may increase exposure to digoxin. Patients who require digoxin should therefore be monitored carefully.

Oral anti-coagultants

Oral anti-coagulants, e.g. rivaoxuban, for the treatment of pulmonary embolism should be avoided within 24 hours of administration of fulvestrant

<u>Medications permitted without caution</u>

CYP3A4 inhibitors	Hemp oil	Fentanyl (e.g. skin patches)
CYP2D6 inhibitors and inducers	Calcium	Alfentanil spray
Flu vaccination	Vitamins D and C	Cod liver oil
Flexiseq	Zine	Losartan
Buclizine	Glucosamine	Heparin, e..g dalteparin
Asthma inhaler, e.g. RElvar Ellipta	Antihistamines ,e.g. Cinnarizine	

<u>Concomitant radiotherapy</u>

Currently, limited information is available regarding the safety and therapeutic benefit of the combination of vandetanib and radiotherapy. Thus, the investigator may use his/her own discretion of whether to stop or continue vandetanib/placebo during the radiation therapy ensuring careful safety monitoring.

<u>Concomitant dental treatment</u>

Dental treatment is allowed and normal cautions with concomitant medication apply.

<u>Concomitant surgery</u>

Vandetanib can act as an antiangiogenic. Concomitant surgery under anaesthetic is permitted unless a significant healing process is required, in which case vandetanib should be withheld at the local PI discretion (see Section Section 8.1.2 Dose modifications).

CONFIDENTIAL

This material is the property of the Wales Cancer Trials Unit. Do not disclose or use except as authorised.

8.1.5 Drug supply, distribution and storage

AstraZeneca will fund and supply both the investigational products (vandetanib (also known as ZD6474; IMP) or matching placebo)) and the non-investigational product (Fulvestrant; nIMP).

All study drugs will be packaged, labelled and distributed by Fisher Clinical Services (FSC) in accordance with local regulations and Good Manufacturing Practice, stating that the drug is for clinical use only and should be kept out of the reach and sight of children.

IMP/Placebo

The IMP/Placebo will be supplied as round, plain, biconvex, white film coated tablets:

Identity of investigational product

Investigational product	Form	Dosage strength	Manufacturer
Vandetanib (ZD6474)	Tablet	300mg	AstraZeneca
Vandetanib (ZD6474)	Tablet	100mg	AstraZeneca
Placebo to Vandetanib	Tablet	N/A	AstraZeneca

IMP/Placebo tablets will supplied in white high density polyethylene (HDPE) Child Resistant bottles. The bottles should be stored below 30°C. The packaging and appearance of IMP tablets will appear identical to the matching placebo treatments. However, the 100mg IMP/placebo tablets are not identical to the 300mg IMP/placebo tablets.

A detachable peel-off trial-specific label will be affixed to each blinded IMP/Placebo container and will contain space for centre number, participant trial number and date of dispensing to be completed. The label will have a unique treatment kit number that is linked to the randomisation scheme. The completed label will be attached to the subject level drug accountability log at the time of dispensing as described in Section 8.1.6 of this Protocol.

nIMP

nIMP will be supplied in cartons of two 5ml pre-filled syringes; each syringe containing 250mg fulvestrant solution for injection, and labelled with the standard commercial label, with no trial specific labelling. nIMP kits will be presented in their commercial format and will not be labelled with any trial specific information. nIMP should be stored at 2 to 8°C”. It should be stored in the original packaging and protected from light. It should be ringfenced in a designated area away from local hospitals stocks of fulvestrant and used for FURVA participants only.

IWRS

When a site is first activated it will be provided with an initial supply of the IMP/Placebo in blinded “kits” (bottles) of tablets and the nIMP. Re-supply is automatic when a site’s stock falls below a minimum level.

This material is the property of the Wales Cancer Trials Unit. Do not disclose or use except as authorised.

An on-line system called IWRS - for which a user guide will be provided (see the Cenduit "Study Site User Guide") – is used to:

- Confirm receipt of drug shipment (site Pharmacy will need do this).
- Dispense IMP/Placebo (bottles) and nMP (cartons) during treatment (Research Nurse/Pharmacist will do this) and receive IMP/Placebo kit number/s that should be allocated.
- Change participant status (e.g. withdraw from treatment)/unblind participants.
- Manage expiry dates – expired stock will not be dispensed to patients and resupply of new stock will be automatic.

The IWRS will initially be set-up for 49 months from when the trial opens to recruitment to cover the trial recruitment period and minimum follow-up of 12 months. If patients require drugs outside of this timeframe and IWRS cannot be extended, AstraZeneca will provide an alternative mechanism for issuing drugs (see further information regarding duration of drug supply below).

Patients enrolled in the study will be dispensed bottles of blinded IMP or Placebo tablets; each bottle will contain IMP or Placebo tablets as determined by the randomisation scheme.

At the beginning of each cycle, patients will be supplied with sufficient IMP/Placebo to last one cycle. There will be sufficient tablets in the bottle to cover the 28 day visit window. The patient will be asked to return any remaining tablets and bottles at the start of the next cycle. Returned drugs and bottles should be returned to pharmacy, logged on the subject level accountability log and destroyed following Section 8.1.6 of this Protocol.

At the beginning of each cycle, and on day 15 of Cycle 1 only, nurses will be supplied with one carton of nIMP for administration to the patient by site staff.

<u>Temperature excursions</u>

Participating sites will report any temperature excursions for IMP/Placebo or nIMP to WCTU as soon as they are discovered following the procedure described in the FURVA Pharmacy Information Sheet and FURVA Drug Enquiry Form and quarantine the stock immediately. WCTU will confirm with AstraZeneca through FSC whether the stock can be used, and if stock cannot be used, then the IWRS will be updated to allow for re-supply.

nIMP is suitable for use without assessment by the supplier if exposed to temperature between -5°C and +30°C for up to 12 days, provided the average temperature of the product storage is less than 25°C. Pharmacy should keep a log of the total time the specific pack/batch of fulvestrant is outside 2 to 8 °C. If a temperature outside -5°C and +30°C is reached, and/or the cumulative time outside of 2 to 8 °C but within 5°C and +30°C range reaches 12 days, the temperature excursion must be reported to the WCTU and FCS.

Duration of drug supply

AstraZeneca divested vandetanib to Genzyme in July 2015 (see Section 15.8 Finance). AstraZeneca stocks of vandetanib expire May 2020, while matching placebo expires April 2019. If FURVA participants require treatment beyond April 2019, treatment allocations will be unblinded at that date by the WCTU trial statistician (see Section 8.2 Blinding), with those patients randomised to vandetanib having the option to continue on unblinded study treatment supplied by AstraZeneca until May 2020 at the latest, and patients randomised to placebo having the option to continue on fulvestrant alone supplied by AstaZeneca indefinitely.

Site to site transfers (even within the same trust) of vandetanib/placebo or fulvestrant are not permitted.

8.1.6 Drug accountability

The medication provided for this study is for use only as directed in the protocol. It is the investigator's responsibility to establish a system for handling the IMP/Placebo and NIMP to ensure that:

- Deliveries of IMP/placebo and NIMP from Fisher Clinical Services are correctly received by a responsible person (e.g. pharmacist) and are handled and stored correctly and safely. Receipt should be acknowledged through the IWRS system.
- IMP.placebo and NIMP are dispensed only to study participants, and in accordance with the protocol.
- Participants return any unused IMP/placebo and all empty containers to the investigator.
- A dispensing record (which will include the identification of the participant to whom the IMP/placebo and NIMP was dispensed, the date of dispensing, the quantity dispensed, and the date and quantity of any unused IMP or NIMP returned to the pharmacy) is accurately maintained. Any discrepancies must be accounted for on the appropriate form. This record is in addition to any drug accountability information recorded in the CRFs.
- It must be possible to reconcile delivery records with dispensing records and records of destroyed or returned stock.

In the case that any study IMP or nIMP is damaged during storage, please contact WCTU for reconciliation and replacement. If the study IMP or nIMP is damaged on arrival, please report it via IWRS (see the Cenduit "Study Site User Guide").

The participants will be given a diary card to keep track of the IMP/placebo tablets they have taken. This must not be returned to the WCTU but should be used to help accurately complete treatment compliance and toxicity CRFs.

The number of tablets of IMP/Placebo returned should be counted and recorded on the accountability log.

CONFIDENTIAL

This material is the property of the Wales Cancer Trials Unit. Do not disclose or use except as authorised.

Returned IMP/Placebo from participants should be destroyed locally at site following local procedures. At the termination of the study or at the request of the Sponsor, all unused drugs will be accounted.

8.1.7 Packaging

There are no special packaging requirements for the IMP or nIMP.

8.1.8 Labelling

The labelling details for the IMP and nIMP are detailed in section 8.1.5.

8.1.9 Drug interactions

See section 8.1.4 for information about drug interactions.

8.1.10 Drug disposal and recall

Sites will obtain permission from the WCTU before disposing of any trial drug. Drugs will be destroyed locally at the study site. Certificates of delivery and destruction or return must be signed and copies retained in the ISF.

If the IMP or NIMP is recalled by the Sponsor, AstraZeneca, or FCS, the WCTU will instruct sites on the product recall process. The WCTU will be responsible for notifying the MHRA, NHS Trusts and Boards and the ethics committee. Fisher Clinical Services recover all recalled supplies within the required timeframe. All recalled stock must be accounted for by the site to allow full drug reconciliation.

8.2 Blinding

This is a double-blind trial. All patients receive nIMP in an unblinded manner, but both patients and clinician are blinded to the IMP/Placebo tablet.

Individual IMP/Placeob treatment kit codes, indicating the treatment randomisation for each randomised participant, will be available to the investigator(s) or pharmacists from the IWRS. The kit code will be different for each bottle of drug. 24 hour unblinding can be performed via the IWRS system. The nominated site pharmacists, FURVA research nurses and the PI all have IWRS accounts. In the event that the investigator (or pharmacist) is not available to unblind the following Cenduit 24 hour helpline is provided: 00 800 1012 1960. Cenduit can unblind as long as an account number is provided and the person has access to the IWRS Unblinding Form. Routines for this will be described in the IWRS user manual that will be provided to each centre.

CONFIDENTIAL

This material is the property of the Wales Cancer Trials Unit. Do not disclose or use except as authorised.

Unblinding must not be performed except in medical emergencies when the appropriate management of the participant necessitates knowledge of the treatment randomisation. If the treatment code is broken then the investigator(s) must document and report to WCTU immediately. Progression of disease or toxicity is not a reason for unblinding and there is no planned cross-over upon progression. Once unblinded, it will not be possible to dispense further IMP/Placebo to the patient, and the treatment withdrawal form should be completed. The patient can continue in the trial follow-up. In the event that the participant cannot be unblinded, it should be assumed that they have been treated with vandetanib.

The WCTU trial statistician and data management team will be blinded to the treatment allocation. The WCTU safety team are the only members of the WCTU to have unblinded access to IWRS. This allows the team to unblind SUSARs and to unblind the trial when required for data review and analysis. The safety team will not have access to the non-safety clinical trial data.

The data reviews by the Independent Data Monitoring Committee (IDMC) will be presented in blinded groupings to the IDMC, although they may request to be unblinded upon review of the data. The WCTU statistician will not be informed of the identity of each group.

The data presented to the MHRA in the DSUR will be unblinded by the WCTU safety team prior to submission. The data presented to participating sites in the ISR will be blinded.

If FURVA participants require treatment beyond April 2019, treatment allocations will be unblinded by the WCTU trial statistician at that date (see Section 8.1.5 Drug supply, distribution and storage).

8.3 Dose banding

Dose banding is not applicable. Study drugs are of a single dose only.

9.0 Trial assessments

A detailed assessment schedule is given in section 9.9.

9.1 Screening assessments

Screening (inclusive of eligibility screening collected after participant consent and within 28 days of date of randomisation):

- Medical history including comorbidities.
- Physical examination including ECOG performance status, weight, blood pressure and pulse.
- Concomitant medication record to include any medication included in the hospital notes.
- Collection of screening blood samples (baseline blood tests include full blood count, urea and electrolytes (including potassium), liver function tests, calcium, magnesium, FSH and estradiol).
- Screening toxicity assessment.

This material is the property of the Wales Cancer Trials Unit. Do not disclose or use except as authorised.

- Screening cardiac assessment (ECG repeated 3 times, to be taken 5 minutes apart using the mean average of the three readings to calculate eligiblity).
- Clinical disease assessment.
- Radiological disease assessment using RECIST Version 1.1. (CT scan of chest, abdomen and pelvis as a minimum. May also include bone scan or other imaging techniques such as MRI if clinically appropriate. Imaging of the head/brain is not mandatory unless there is clinical suspicion of brain metastases). Date of screening CT scan must be within 28 days of the date of randomisation.
- Confirmation that the patient has a FFPE tissue block available for analysis.

9.2 Baseline assessments

Baseline (conducted after consent and randomisation, and prior to Cycle 1 Day 1 treatment):

- Physical examination including ECOG performance status, weight, blood pressure and pulse.
- Concomitant medication record.
- Collection of baseline blood samples (within 72 hours prior to Cycle 1 Day 1 treatment).
- Baseline toxicity assessment.
- Collection of NHS IC flagging data (only if consent given).
- Collection of timepoint 1 (baseline translational blood sample).
- Collection of archival tumour tissue sample (paraffin block). If more than one sample is available only collect the earliest sample that confirmed metastic disease.

9.3 Assessments on treatment (weeks 1-60)

All cycle assessments should be conducted on Day 1 of the cycle before the start of the cycle treatment. Participants will be reviewed by a clinician on Cycle 1, Days 1 and 15 and on weeks 4, 8, 12, 16, 20, 24 and then every 12 weeks (weeks 36, 38 and 60). A trials nurse will review the participant at each fulvestrant administration. Participants will be treated until disease progression (or unacceptable toxicity or withdrawal of consent). The following assessments will be made:

- Treatment details including medication kit numbers for vandetanib/placebo.
- Physical examination including ECOG performance status, weight, blood pressure and pulse.
- Concomitant medication record.
- Collection of blood samples within 72 hours prior to treatment for the following blood tests: full blood count, urea and electrolytes (including potassium), liver function tests, calcium and magnesium.
- Toxicity assessment.
- ECG repeated 3 times (weeks 2, 4, 8, 24, 36, 48, 60).
- Clinical disease assessment (weeks 2, 4, 8, 12, 16, 20, 24, 36, 48 and 60).
- Radiological disease assessment using RECIST Version 1.1 . CT scans will be performed at 8, 16 and 24 weeks after randomisation and at 12 weekly intervals (weeks 36, 48 and 60) thereafter. CT scans will be performed as close as possible to, and within 2 weeks of, the scheduled date of assessment.
- Collection of timepoint 2 translational blood sample (week 8).

CONFIDENTIAL

This material is the property of the Wales Cancer Trials Unit. Do not disclose or use except as authorised.

9.4 Assessments on treatment (weeks 61+)

If participants have not progressed by week 60 they can remain on trial therapy. Assessment of disease will be according to local PIs' practice and trial based monitoring will change to a three monthly review (i.e. week 72, 84, 96 etc.), collecting data on treatment, date of progression, SAEs, trial withdrawal or death only:

- Treatment details including medication kit numbers for vandetanib/placebo.
- Physical examination including ECOG performance status, weight, blood pressure and pulse.
- Concomitant medication record.
- Toxicity assessment.
- ECG repeated 3 times.
- Clinical disease assessment.

9.5 End of treatment assessments

Assessments to be taken when a participant withdraws from IMP:

- Medical history including co-morbidities.
- Physical examination including ECOG performance status, weight, blood pressure, pulse and oxygen saturation.
- Concomitant medication record including any new anti-cancer therapy post-withdrawal from FURVA.
- Toxicity assessment.
- Clinical disease assessment.
- Radiological assessment using RECIST Version 1.1 (if not already performed and the drug is being stopped due to progression).
- Collection of timepoint 3 (end of treatment) translational blood sample

9.6 End of study assessments

Assessments to be taken 30 days after the end of treatment:

- Physical examination including ECOG performance status, weight, blood pressure and pulse.
- Concomitant medication record including any new anti-cancer therapy post-withdrawal from FURVA.
- Toxicity assessment.

9.7 Unscheduled assessments

Data will be collected for the following unscheduled events:

- Unscheduled additional ECGs.

This material is the property of the Wales Cancer Trials Unit. Do not disclose or use except as authorised.

- Withdrawal (refer to section 11.3 of protocol for further details)
- Death
- SAEs (refer to section 10.3 of protocol for further details)

9.8 Completion of CRFs

The FURVA Main Case Report Form (CRF) Booklet should be used to report the following assessment data: screening, randomisation, baseline, NHS Flagging, translational blood and tissue sample collections, treatment weeks 1-64, end of treatment (floating time point), end of study (30 days post-end of treatment), unscheduled ECGs, withdrawal and death.

The FURVA Follow Up CRF Booklet should be used to collect follow up assessment data from week 65 onwards.

The top copy of each completed CRF should be returned to the WCTU for data entry within four weeks of the visit. The remaining copy is to be retained at the local site. In accordance with the principles of GCP, the PI is responsible for ensuring accuracy, completeness, legibility and timeliness of the data reported to the WCTU in the CRFs.

CRF pages and data received by the WCTU from participating trial sites will be checked for missing, illegible or unusual values (range checks) and consistency over time.

If missing or questionable data are identified, a data query will be raised on a data clarification form. The data clarification form will be sent to the relevant participating site. The site shall be requested to answer the data query or correct data on the data clarification form. The case report form pages should not be altered.

All answered data queries and corrections should be signed off and dated by a delegated member of staff at the relevant participating site. The completed data clarification form should be returned to the WCTU and a copy retained at the site along with the participants’ CRFs.

The WCTU will send reminders for any overdue data. It is the site’s responsibility to submit complete and accurate data in timely manner.

CONFIDENTIAL

This material is the property of the Wales Cancer Trials Unit. Do not disclose or use except as authorised.

9.9 Schedule of trial treatments, scheduled and unscheduled assessments, and translational sample collection timepoints

	Informed consent	Screening[1]	Randomisation[2] (including [illegible]	Cycle 1 Day 1[3] (Baseline)	Cycle 1 Day 15	Cycle 2 Day 1	Cycle 3 Day 1	Cycle 4 Day 1	Cycle 5 Day 1	Cycle 6 Day 1	Cycle 7, 10, 13, and 16 Day 1[4]	3 monthly follow-up	End of treatment [13]	End of study[14]
**Week**	**-4**	**-4 to 0**	**0**	**1**	**3**	**5**	**9**	**13**	**17**	**21**	**25, 37, 49, 61**	**65+**	**Floating time point**	**Floating time point**
**Trial Treatments**														
Fulvestrant administration				X	X	X	X	X	X	X	X	X[12]		
Vandetanib/placebo (daily)				←								→		
**Scheduled Assessments**														
Medical history including co-morbidities and dates of last treatments		X											X	
Physcial examination[5]		X		X	X	X	X	X	X	X	X	X	X[6]	X
Concomitant medications		X		X	X	X	X	X	X	X	X		X	X
Blood assessements[7]		X		X	X	X	X	X	X	X	X			
Toxicity and adverse event assessment		X		X	X	X	X	X	X	X	X	X	X	X
ECG/QTc evaluation x 3 (5 mins apart)		X			X	X	X				X	X		
Clinical disease assessment		X		X	X	X	X	X	X	X	X	X	X	
Radiological disease assessment (scan)[8]		X					X		X		X		X[9]	
Treatment details including medication kit numbers				X	X	X	X	X	X	X	X	X		
NHS IC Flagging data				X										
**Unscheduled Assessments[10]**														
Unscheduled additional ECGs				If required										
Withdrawal														
Death														
**Translational samples[11]**														
Blood sample timpoint				1			2						3	
Translational archival tumour tissue sample (paraffin block)				1										

1 All screening assessments, including screening CT scan, should be conducted within 28 days of the date of randomisation.
2 Patients should be randomised within 42 days of the date of consent.

3 All cycle assessments are done on day 1 before the start of the cycle treatment. Participants will also be seen on Day 15 of Cycle 1.
4 All cycle assessments (except CT scans – see 8) are done on day 1 before the start of the cycle treatment. Until radiological progression, then are withdrawn and switched to three monthly review of treatment, date of progression, SAEs, trial withdrawal or death only.
5 Including ECOG performance status, weight, blood pressure, and pulse.
6 Oxygen saturation assessed at screening and end of treatment only.
7 With the exception of the screening assessment, all bloods should be taken within 72 hours of treatment and the results reviewed prior to administration of the next cycle of treatment. Blood assesments to include sodium, potassium, urea, creatine, albumin, transaminase (ALT or AST), ALP, bilirubin, corrected calcium, magnesium, eGFR, haemoglobin, white blood cell count, neutrophils, and platelets. FSH and estradiol (irrespective of other indicators of menopausal status), and prothrombin time, to be collected at screening assessment only.
8 To include cross sectional imaging of chest and abdomen (and, if clinically indicated, pelvis) during screening and at weeks 8, 16, 24, and then 36, 48 and 60. CT scans should be performed as per clinical practice. Other RECIST v1.1 compatible imaging will be allowed e.g. MRI as long as the same method is used throughout the study. Scan timelines to be calculated from Cycle 1 Day 1. Scans will be performed as close as possible to, and within 2 weeks of, the scheduled date of assessment. Disease progression should be assessed using RECIST 1.1. Guidelines provided in Appendix 1: RECIST v1.1.
9 Only required if the patient has withdrawn from treatment because of disease progression and a CT scan confirming disease progression has not already been performed.
10 Unscheduled assessments conducted only if required.
11 Translational samples should only be collected if consent to do so has been provided by the participant on the main study consent form.
12 Patients that have not disease progressed as assessed by CT scan RECIST on Cycle 16 Day 1 (week 61) can continue on trial treatment from week 61 onwards indefinitely at the discretion of the local PI.
13 End of treatment assessment conducted when participant withdraws from IMP, i.e. floating time point.
14 End of study assessment conducted 30 days after the end of treatment assessment, i.e. floating time point.

.

CONFIDENTIAL

This material is the property of the Wales Cancer Trials Unit. Do not disclose or use except as authorised.

10.0 Safety reporting and pharmacovigilance

The Principal Investigator is responsible for ensuring that all site staff involved in this trial are familiar with the content of this section.

The following definitions are in accordance with the Medicines for Human Use (Clinical Trials) Regulations 2004 (SI2004/1031) (as amended) and EU Directive 2001/20/EC.

Term	Definition
**Adverse Event (AE)**	Any untoward medical occurrence in a patient or clinical trial participant administered a medicinal product and which does not necessarily have a causal relationship with this treatment. An adverse event can therefore be any unfavourable and unintended sign (including an abnormal laboratory finding for example), symptom or disease temporally associated with the use of a medicinal product, whether or not considered related to the medicinal product.
**Adverse Reaction (AR)**	Any untoward and unintended response in a clinical trial participant to an investigational medicinal product which is related to any dose administered to that participant
**Serious Adverse Event (SAE)**	Any adverse event that -<br>• Results in death<br>• Is life-threatening*<br>• Required hospitalisation or prolongation of existing hospitalisation**<br>• Results in persistent or significant disability or incapacity<br>• Consists of a congenital anomaly or birth defect<br>• Other medically important condition ***<br><br>For the purposes of this trial the following events will not require immediate reporting<br>• Death due to disease progression<br>• Hospitalisation for blood transfusion<br><br>These should be completed in the participants' notes and on the relevant toxicities CRF page and forwarded to the WCTU in the normal timeframes for CRFs.
**Serious Adverse Reactions (SARs)**	Any SAE occurring in a clinical trial participant for which there is a reasonable possibility that it is **related** to the IMP at any dose administered.

This material is the property of the Wales Cancer Trials Unit. Do not disclose or use except as authorised.

**Suspected Unexpected Serious Adverse Reactions (SUSARs)**	A SAR, the nature and severity of which is not consistent with the Reference Safety Information (RSI) for the IMP.

***Note:** The term 'life-threatening' in the definition of serious refers to an event in which the trial participant was at risk of death at the time of the event or it is suspected that used or continued use of the product would result in the subject's death; it does not refer to an event which hypothetically might have caused death if it were more severe.

**** Note:** Hospitalisation is defined as an inpatient admission, regardless of the length of stay, even if the hospitalisation is a precautionary measure for continued observation. Pre-planned hospitalisation e.g. for pre-existing conditions which have not worsened, or elective procedures, does not constitute an SAE.

***** Note:** other events that may not result in death, are not life-threatening, or do not require hospitalisation, may be considered as an SAE when, based upon appropriate medical judgement, the event may jeopardise the participant and may require medical or surgical intervention to prevent one of the outcomes listed above.

10.1 Causality Assessments

The Principal Investigator (or another delegated medically qualified doctor from the trial team) and Chief Investigator (or another medically qualified doctor from the Trial Management Group) will assess each SAE to determine the causal relationship with the IMP, and will answer 'yes' or 'no' to the question "Do you consider that there is a reasonable possibility that the SAE may have been caused by the IMP?"

For SAEs causal relationship will also be assessed for other trial treatments (nIMPs) and procedures.

**IMPs:** Vandetanib<br>**nIMPs:** Fulvestrant

The causality assessment given by the Principal Investigator (or delegate) cannot be downgraded by the Chief Investigator (or delegate), and in the case of disagreement both opinions will be provided.

A guide to the interpretation of the causality question is found in Appendix 3 of this clinical trial protocol.

10.2 Expectedness Assessments

The Chief Investigator (or another delegated appropriately qualified individual) will assess each SAE to perform the assessment of expectedness.

The expectedness assessment should be made with reference to the current Reference Safety Information (RSI), and must be applied to all IMPs in the trial. Expectedness decisions must be based

This material is the property of the Wales Cancer Trials Unit. Do not disclose or use except as authorised.

purely on the content of the RSI; other factors such as the participant population and participant history should not be taken into account. Expectedness is not related to what is an anticipated event within a particular disease.

SAEs which add significant information on specificity or severity of a known, already documented adverse event constitute unexpected events. For example, an event more specific or more severe than that described in the RSI is considered unexpected.

The table below lists the RSIs that should be referenced:

IMP	RSI to be used for expectedness assessment	Relevant section of RSI to be used for expectedness assessment
Vandetanib/Placebo	IB	Section 5.4

10.3 SAE reporting

10.3.1 Participating Site Responsibilities

All SAEs must be reported immediately (and within 24 hours of knowledge of the event) by the PI at the participating site to the WCTU unless the SAE is specified as not requiring immediate reporting (see section 10.0). This includes SAEs related to IMPs and non-Investigational Medicinal Products (nIMPs).

The PI (or delegated medically qualified doctor from the trial team) should sign and date the SAE CRF to acknowledge that he/she has performed the seriousness and causality assessments.

A completed SAE form for all events requiring immediate reporting should be emailed to the WCTU within 24 hours of knowledge of the event. A separate form and email cover sheet must be used to report each event, irrespective of whether or not the events had the same date of onset.

The participant will be identified only by trial number, date of birth and initials. The participant's name should not be used on any correspondence.

It is also required that sites respond to and clarify any queries raised on any reported SAEs and report any additional information as and when it becomes available through to the resolution of the event.

Serious Adverse Event (SAE) Contact Details:

Email: CTR-Safety@cardiff.ac.uk

Fax (emergency use only) : 029 2064 4488

Serious adverse events should be reported from time of signature of informed consent, throughout the treatment period up to, and including 30 days after the participant receives their last dose of the IMP.

This material is the property of the Wales Cancer Trials Unit. Do not disclose or use except as authorised.

Serious adverse reactions (such as long term side effects of trial treatment under investigation) should continue to be reported until the end of follow up.

Adverse events (AE) should be graded using the NCI Common Terminology Criteria for Adverse Events (CTCAE) Version 4.03. The toxicity grades should be recorded on the toxicity part of the CRF.

An SAE form is not considered as complete unless the following details are provided:

- Full participant trial number
- An Adverse Event / Adverse Reaction
- A completed assessment of the seriousness, and causality as performed by the PI (or another appropriately medically qualified doctor registered on the delegation log).

If any of these details are missing, the site will be contacted and the information must be provided by the site to the WCTU within 24 hours.

All other AEs should be reported on the CRF following the CRF procedure described in Section 11.1.

10.3.2 The WCTU responsibilities

Following the initial report, all SAEs should be followed up to resolution wherever possible, and further information may be requested by the WCTU.

The WCTU should continue reporting SAEs until 30 days after the participant receives their last dose of the IMP. Serious adverse reactions (SARs) should continue to be reported until the end of follow up.

Once an SAE is received at the WCTU, it will be evaluated by staff at the WCTU and sent to the CI (or their delegate) for an assessment of causality and expectedness.

Investigator reports of suspected SARs will be reviewed immediately and those that are identified as SUSARs are reported to the MHRA, Main Ethics Committee and AstraZeneca.

10.4 SUSAR reporting

Velindre NHS trust is undertaking the role of trial Sponsor and has delegated to the WCTU the responsibility for reporting SUSARs and other SARs to the regulatory authorities (MHRA and relevant ethics committees) and to AstraZeneca as follows:

SUSARs which are fatal or life-threatening must be reported to the MHRA and MREC within 7 calendar days of receipt at the WCTU. Any additional, relevant information must be reported within a further 8 calendar days of submitting the initial report.

SUSARs that are not fatal or life-threatening must be reported to the MHRA and MREC within 15 days of receipt at the WCTU. Any additional, relevant information must be reported within a further 15 days.

N.B. There is no requirement for WCTU to report SUSARs to nIMPs to the MHRA except in the following instances:

This material is the property of the Wales Cancer Trials Unit. Do not disclose or use except as authorised.

- If the adverse reaction is suspected to be linked to an interaction between a nIMP and IMP, and is serious and unexpected, WCTU should report as a SUSAR due to the interaction with the IMP.
- If a SUSAR is suspected and might be linked to either a nIMP or an IMP and cannot be attributed to only one of these.
- If the adverse reaction due to the nIMP is likely to affect the safety of trial subjects then WCTU should report it to the MHRA and Main Ethics Committee in accordance with the relevant Standard Operating Procedure for reporting Urgent Safety Measures.

10.5 Safety Reports

A list of all SARs (expected and unexpected) will be reported annually to the MHRA, Main Ethics Committee, and Astrazeneca in an unblinded Development Safety Update Report (DSUR). This report must be submitted within 60 days of the anniversary of the MHRA CTA approval date. The Sponsor will receive a blinded version of the DSUR.

The Principal Investigators will also receive a list of SARs, in the form of Investigator Safety Reports (ISRs), which will be produced every six months during trial treatment and then annually during follow-up.

CONFIDENTIAL

This material is the property of the Wales Cancer Trials Unit. Do not disclose or use except as authorised.

10.6 Flowchart for Serious Adverse Event reporting

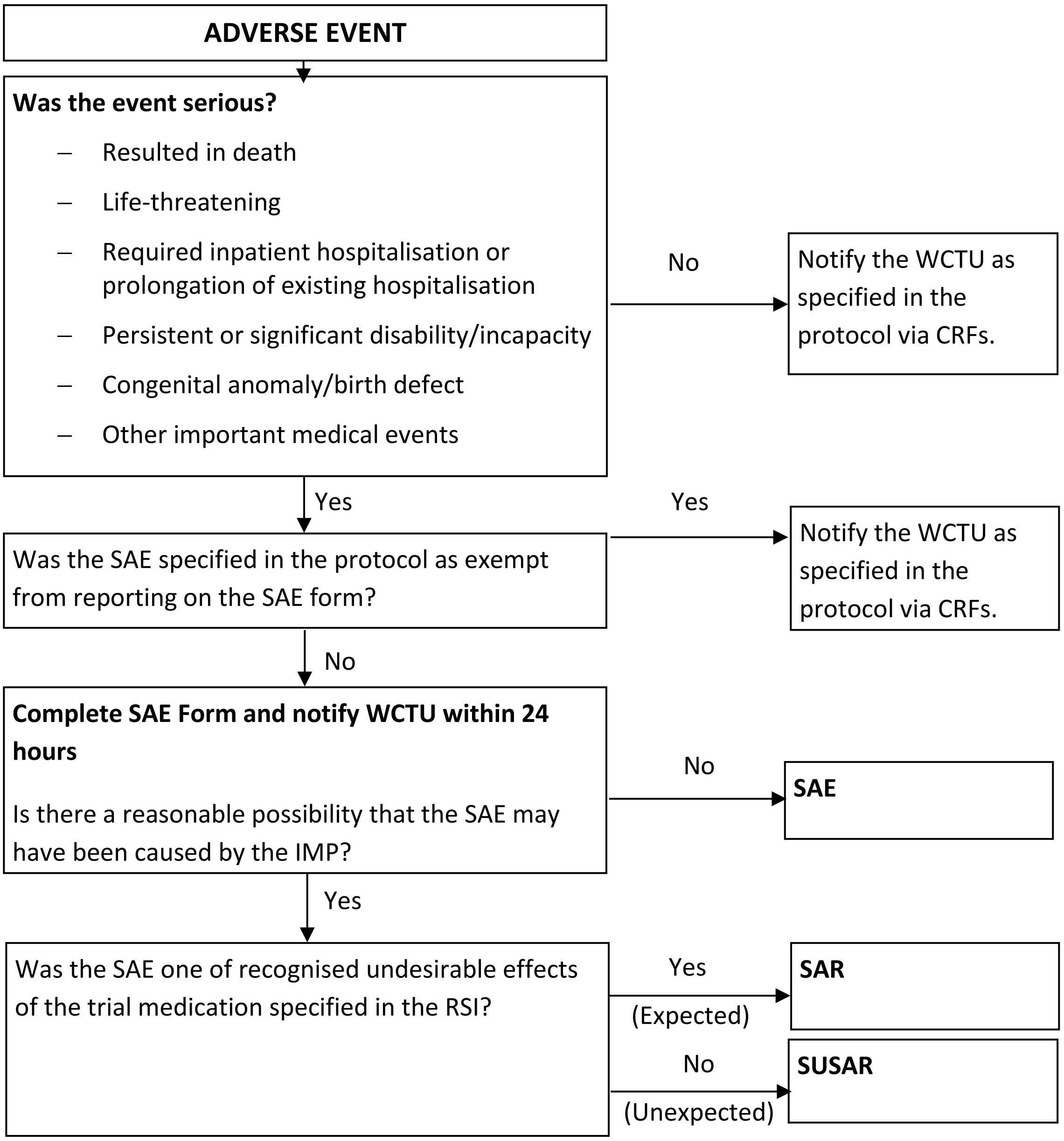

CRF Case Report Form

RSI Reference Safety Information

SAE Serious Adverse Event

SAR Serious Adverse Reaction

SUSAR Suspected Unexpected Serious Adverse Reaction

WCTU Wales Cancer Trials Unit

This material is the property of the Wales Cancer Trials Unit. Do not disclose or use except as authorised.

11.0 Trial management

11.1 Trial committees and trial management

The conduct of the trial is being overseen by the following committees:

- The Trial Management Group (TMG) will be responsible for the day-to-day running of the trial and will meet at least once every six months. The TMG members will include the Chief Investigator, other active trial investigators, WCTU representatives, and specialist advisors (e.g. Pharmacist, Statistician, consumer representative, translational expert).
- The data will be reviewed (approximately six monthly) by an **Independent Data Monitoring Committee** (IDMC), consisting of at least two Clinicians (not entering patients into the trial) and an independent Statistician. The IDMC will be asked to recommend whether the accumulated data from the trial, together with results from other relevant trials, justifies continuing recruitment of further patients. A decision to discontinue recruitment, in all patients or in selected subgroups, will be made only if the result is likely to convince a broad range of Clinicians including PIs in the trial and the general clinical community. If a decision is made to continue, the IDMC will advise on the frequency of future reviews of the data on the basis of accrual and event rates. The IDMC will make confidential recommendations to the Trial Steering Committee (TSC).
- Independent Trial Steering Committee (TSC): The TSC will be a committee of independent members that provides overall supervision of the trial. The role of the TSC is to act on behalf of the Sponsor, to provide overall supervision for the trial, to ensure that it is conducted in accordance with GCP, and to provide advice through its independent chairman. The TSC will review the recommendations from the IDMC and will decide on continuing or stopping the trial, or modifying the protocol. It will meet at least annually when it will consider each report of the IDMC, as well as results of other trials and new information which has arisen, and recommend appropriate action.

11.2 Monitoring

The clinical trial risk assessment has been used to determine the intensity and focus of central and on-site monitoring activity in the FURVA trial. Moderate monitoring levels will be employed and are fully documented in the trial monitoring plan.

Investigators should agree to allow trial related monitoring, including audits and regulatory inspections, by providing direct access to source data/documents as required. Patient consent for this will be obtained.

11.3 Participant Withdrawal

In consenting to the trial, participants are consenting to trial treatment, trial follow-up and data collection. Participants may withdraw from the trial at any time.

Patients may withdraw at one of 4 levels:

Level 1: Withdraw from trial treatment – participants stop trial treatment (IMP, NIMP, or both) but remain in follow-up and continue to provide translational samples.

CONFIDENTIAL

This material is the property of the Wales Cancer Trials Unit. Do not disclose or use except as authorised.

Level 2: Withdraw from the translational study – participants continue trial treatment (IMP and NIMP) and follow-up but do not provide translational samples

Level 3: Withdrawal from the translational study and trial treatment - participants stop trial treatment (IMP, NIMP or both) and do not continue providing translational samples but remain in follow-up.

Level 4: Completely withdraw from the trial – participants stop trial treatment (IMP and NIMP), follow-up and any translational sample collection.

Withdrawal for any reason requires a completed Withdrawal CRF to be faxed to the WCTU as soon as the withdrawal information becomes available, with the hard copy to follow soon after.Participants do not have to give a reason for their withdrawal but sites should make a reasonable attempt to find out why and should record the reason on the Withdrawal CRF. Up to three Withdrawal CRFs can be submitted for each participant to report changes to participant withdrawal status, e.g. level and/or reason.

A participant may withdraw, or be withdrawn, from trial treatment for the following reasons:

- Intolerance to treatment (including SAEs and toxicities)
- Disease progression
- Participant choice
- Clinician's decision
- Incorrect enrolment i.e., the participant does not meet the required inclusion/exclusion criteria for the study
- Participant lost to follow-up
- Non-concordance with protocol treatment
- Other (please specify)

If participants withdraw at Level 1 or 3, then the site should confirm on the Withdrawal CRF if the patient has withdrawn from IMP, NIMP or both.

If a participant withdraws from NIMP trial treatment only (i.e. remains on IMP) then participating sites should continue IMP treatment and the Cycle 1-16 follow-up schedule as normal until the participant subsequently withdraws from IMP due to disease progression, toxicity or death.

If a participant withdraws from IMP, then the End of Treatment CRFand the End of Study (30 days after end of treatment CRF) should be completed.

If the participant withdraws from IMP treatment for reasons other than disease progression, then the Cycle 1-16 follow-up schedule should be followed as normal except ECGs which are not required unless the patient experienced a significant change in ECG from their baseline reading. It is optional for the participant to continue monotherapy with NIMP sourced from trial stocks for the remainder of the Cycle 1-16 trial treatment phase providing the monotherapy is recorded on the treatment section of the Cycle 1-16 CRFs.

This material is the property of the Wales Cancer Trials Unit. Do not disclose or use except as authorised.

If a participant disease progresses they should withdraw from both IMP and NIMP. Disease progression data should be recorded on the End of Treatment CRF. Only if the withdrawal is at level 1 should an end of treatment (time point 3) translational blood sample also be collected. No further assessments or CRF completion is required other than updated withdrawal CRF and death CRF data where applicable.
Data and samples collected prior to participant withdrawal at any of the four levels indicated above will be collected and used for trial analysis by the WCTU. Participants who initially consented to be registered with the National Health Service Information Centre (NHSIC) or equivalent will remain on the system so that important research information on date and cause of death can be requested from NHSIC by the WCTU.

If a participant explicitly withdraws consent to have any data recorded, their decision must be respected and recorded on the Withdrawal CRF. Details of the CRF should be noted in the participant records and no further FURVA CRFs should be completed for the participant.

If a participant withdraws at Level 2, 3 or 4 (complete withdrawal) complete the End of Treatment CRF, if possible with any information available on the date of withdrawal from IMP. Do not complete the End of Study CRF. Do not conduct any follow-up assessments.

If a participant withdraws at Level 2, 3 or 4 do not collect any translational samples that are due after the date of withdrawal.

If a patient withdraws at Level 1 *after* consent and randomisation but *before* administration of Cycle 1 Day 1 treatment do not collect the baseline (time point 1) translational blood sample or baseline FFPE tissue block. No further assessment or CRF completion is required.

On receipt of a withdrawal form at the WCTU, the WCTU will contact the site to confirm the level of withdrawal.

11.4 Lost to follow-up

If a participant is lost to follow up the WCTU will request that the PI contacts the participant's GP to obtain information on the participant's status. Participants have the option to consent to NHSIC Flagging. This will entail completion of a separate consent form which will contain the participant name, postcode, date of birth and NHS Number (or equivalent – e.g. CHI number) and will therefore be kept separate from the other data, and securely locked away. This will enable the WCTU to trace the participant cause and date of death.

11.5 Protocol/GCP non-compliance

The PI should report any non-compliance to the trial protocol or the conditions and principles of GCP to the WCTU as soon as they become aware of it.

CONFIDENTIAL

This material is the property of the Wales Cancer Trials Unit. Do not disclose or use except as authorised.

11.6 The End of the Trial

The Cycle 1 to 16 treatment phase will be followed by a non-interventional follow-up period which will continue to observe overall survival in study patients. All participants will be followed up for SAEs until 30 days after all trial treatment is withdrawn.

For the purposes of both MHRA and Research Ethics Committee approval, the study end date is deemed to be the date of last data capture. This will be the last patient's study visit, or when 98 disease progression events have been reported, whichever is later. After this point, data on survival will be obtained from the NHS Information Centre flagging service.

11.7 Archiving

The TMF and TSF containing essential documents will be archived at an approved external storage facility for a minimum of 15 years. The WCTU will archive the TMF, TSFs and participant files on behalf of the Sponsor. The PI is responsible for archival of the ISF, Patient Trial Files, and Pharmacy File at site. Essential documents pertaining to the trial shall not be destroyed without permission from the Sponsor. Any participant diary cards returned to the site must be archived as part of the ISF.

This material is the property of the Wales Cancer Trials Unit. Do not disclose or use except as authorised.

12.0 Statistical considerations

12.1 Randomisation

Randomisation will take place centrally via the IWRS. Participants will be randomised using the method of minimisation with a random element. This will ensure balanced treatment allocation by a number of clinically important stratification factors. Randomisation will have an allocation ratio of 1:1.

12.2 Outcome measures

12.2.1 Primary outcome measure

- Progression-free survival (PFS - time to event) based on Response Evaluation Criteria in Solid Tumours (RECIST) v1.1.

12.2.2 Secondary outcome measures

- Safety, tolerability (side effects) and feasibility of use (number of participants requiring dose delays or reductions and/or treatment withdrawal).
- Objective response rate and clinical benefit rate as assessed by RECIST v1.1.
- Overall survival (OS), time from enrolment to death with those still alive censored at date last seen.
- Exploratory analysis: The influence of RET signalling pathway components expression on vandetanib activity.

12.3 Sample size calculation

The sample size was calculated for a Phase 2 screening design, based on a primary outcome of progression free survival, with a time-to-event hazard ratio of 0.65, and with 90% power and a one-sided significance of 20% and assuming an overall loss to follow-up of 10%. Assuming that the estimated PFS in the control arm is 5.4 months, and that we will have an 18 month accrual period and a 12 month follow-up, then a total of 98 events will be required overall, and we would recruit 120 patients to detect 98 events within this timeframe, however to allow us to look at the effect of RET overexpression, we will recruit an additional 40 patients as outlined below.

It is expected that at least 30% of participants will have RET overexpression, and these patients may respond better to vandetanib treatment. With 160 participants, we will have a sub group of approximately 50 participants with RET overexpression to be evaluated for activity. 50 participants will be enough to detect an HR of 0.5 in this subgroup, keeping all other sample size parameters the same, and will provide more information towards the design of a phase III trial.

12.4 Statistical analyses

A statistical analysis plan will be developed before the first analysis of the trial and will be signed off prior to unblinding the data for analysis.

The WCTU trial statistician will be provided with blinded treatment groupings prior to analyses for the safety run-in, IDMC analysis and final report. Blinded group interim reports prior to final data lock will

This material is the property of the Wales Cancer Trials Unit. Do not disclose or use except as authorised.

only be seen by the WCTU trial statistician and the IDMC. Upon request, the IDMC will be given access to the unblinding code for the treatment grouping by the WCTU safety officer, but the WCTU statistician will remain blinded. The final clinical study report will be unblinded.

Safety will be assessed by the IDMC when 20 and 40 patients have completed one cycle of trial treatment.

The trial data will be analysed when all participants have completed a minimum 64 week follow-up from Cycle 1 Day 1 treatment onwards and at least 98 disease progression events are observed. Disease progression will be formally assessed according to the RECIST v1.1 criteria.

At the end of the trial both an intention-to-treat (all results analysed according to the participants' original trial arm allocation) and a per-protocol analysis will be carried out. We will attempt to trace participants who are lost to follow-up via their GP or through the NHSIC or equivalent. Where no information is available these participants will be censored at the date last seen.

Primary analysis will be of PFS described using Kaplan-Meier curves in both arms of the trial. The median PFS will be calculated for each arm of the trial, and then the one-sided logrank test will be then used to formally test the equality of the survivor functions. If the hazards are proportional, then Cox regression will also be performed to adjust the hazard ratio for the stratification factors.

12.5 Sub-group analyses

If the RET signalling pathway status results are available at the time of the main analysis, the PFS, overall survival and objective response rate will be summarised according to the RET status, where patients will be categorised as either RET over expressers, or RET normal. If hazards are proportional, a Cox regression will be performed to calculate the Hazard ratios seen in the RET expression sub-groups.

This material is the property of the Wales Cancer Trials Unit. Do not disclose or use except as authorised.

13.0 Translational research

An exploratory element of the main study will investigate whether the genetic make-up of cancer can predict whether patients will benefit from the drugs used in this study. Translational blood and tissue samples will be collected and stored for future translational analysis.

A Material Transfer Agreement between the host care organisation and the Sponsor must be in place prior to the collection and transfer of translational samples. The site is responsible for collecting the translational samples at the time points listed in the schedule of trial assessments detailed in Section 9.6 of this protocol. Instructions for collecting and transferring samples are detailed in the FURVA Laboratory Manual. Sample data will be collected on the translational sample CRFs included in the main CRF booklet. Samples will be transferred to, stored at, processed by, and tested by research laboratories approved by the Sponsor.

Whole blood samples will be sent to the All Wales Genetic Laboratory (AWGL), spun and the plasma stored pending future analysis. The buffy coat derived from the baseline blood sample will also be stored pending future extraction of germline DNA and analysis. In the event that the analyses do not take place, or, having taken place, some plasma or buffy coat remains, the Sponsor shall arrange for the transfer of the remaining samples to the Wales Cancer Bank (WCB).

Paraffin blocks and associated anonymised pathology reports will be stored long term at the WCB which holds an HTA tissue bank license and has made arrangements with the WCTU to maintain this license as long as it holds the material. In the event that the WCB is unable to continue to hold the material, the Sponsor shall arrange for the transfer of the material to another suitably licensed facility.

All tissue will be supplied anonymously to researchers; only the local hospital staff will be able to identify which samples a patient has donated. Minimum information will be supplied to each laboratory. Only the patient's study number, initials and date of birth will be used to identify their research blood samples. Only their study number, initials, date of birth, the date the biopsy was taken, and the histology block reference number will be used to identify research tissue samples.

The research will include but not be limited to the following analyses. Archival tumour samples will be examined for total RET protein levels by immunohistochemical analysis. This will form the basis of the subgroup exploratory analysis to identify if the effect of vandetanib is limited to patients with activated RET signalling. Other biomarkers may also be interrogated to guide which patients from this population may gain particular benefit. The pharmacokinetic/pharmacodynamics relationship between vandetanib and fulvestrant exposure and biomarkers in blood and anti-tumour actitivty will be assessed. DNA extracts from tissue and blood may also be used in genetic research to investigate specific genes and/or mutations.

CONFIDENTIAL

This material is the property of the Wales Cancer Trials Unit. Do not disclose or use except as authorised.

Separate funding will be sourced for the analysis of the research blood and tissue samples. Any tests being done on the stored samples (and derivatives thereof), other than those related to the FURVA trial, will require separate ethical approval. We will not need to contact the patient about this as the consent they give on the main study consent form will cover these tests.

Patients will be told the results of their monitoring blood tests, but will not be told of any specific results arising from research on their translational samples (and derivatives thereof). Individual results will not be passed on to other parties outside of this study.

Section 11.3 (Participant withdrawal) of this protocol provides guidance regarding withdrawal from the translational part of the study.

Samples will not be sold and will not be used for animal research or in the commercial sector.

The Sponsor shall use its best endeavours to permit the return of specific diagnostic samples to the site upon request if required for the care of the donor.

The samples are expected to continue to be held after the ethics approval for the study has expired and may continue to be held by the Sponsor for as long as the Sponsor deems appropriate provided that, if it is relevant material as defined by the Human Tissue Act, it is stored at premises licensed in accordance with the Human Tissue Act 2004 and in accordance with the consent under which it was obtained.

In the event that the Sponsor deems it no longer appropriate to hold any or all of the samples, the TSC will consider whether the samples should be destroyed, and, if destruction is recommended by the TSC, the Sponsor shall have absolute discretion to arrange the disposal of any or all of the samples.

Disposal of any samples which is relevant material as defined by the Human Tissue Act 2014 will be carried out in accordance with the policies of the WCB and The Human Tissue Act 2004.

This material is the property of the Wales Cancer Trials Unit. Do not disclose or use except as authorised.

14.0 Publication policy

Data from all sites will be analysed together and published as soon as possible. Individual participating PIs may not publish data concerning their participants that are directly relevant to questions posed by the trial until the TMG has published its report. The TMG will form the basis of the writing committee and advise on the nature of publications, subject to the Sponsor's requirements.

All publications should include a list of participating PIs, and if there are named authors, these should include the CI, Co-Investigators, Trial Manager, and Statistician(s) involved in the trial, as agreed by the CI and Director of WCTU. If there are no named authors then a writing committee will be identified.

CONFIDENTIAL

This material is the property of the Wales Cancer Trials Unit. Do not disclose or use except as authorised.

15.0 Ethical and regulatory considerations, and Informed Consent

15.1 Ethical approval

This protocol will be submitted to a Multi-centre Research Ethics Committee (MREC) that is legally "recognised" by the United Kingdom Ethics Committee Authority for review and approval. The approval of the MREC must be obtained before the start of a clinical trial or any trial procedures are conducted.

15.2 Clinical Trial Authorisation (CTA)

The trial is being performed under a Clinical Trial Authorisation (CTA) from the MHRA. The Clinical Trials Authorisation (CTA), the approval of the MHRA, must be obtained before the start of the trial in accordance with Part 3, Regulation 12 of The Medicines for Human Use (Clinical Trials) Regulations 2004 (SI2004/1031).

15.3 Regulatory considerations

Substantial amendments to this Protocol must be approved by the MREC responsible for the study and MHRA (where applicable), before the implementation of the amendments. Minor amendments will not require prior approval by the MREC and MHRA.

If the trial is temporarily halted it will not be recommenced without reference to the MREC responsible for the study and the MHRA.

The MREC and MHRA will be notified within 90 days of trial completion. If the trial is terminated early, the MREC and MHRA will be notified of this within 15 days.

A summary of the clinical trial report will be submitted to the MREC responsible for the study and MHRA within one year of the end of trial.

15.4 Research governance approval

This trial protocol will be submitted through the Research Governance process of the permissions co-ordinating system for global governance review and the local host care organisation for review and approval. The Research Governance approval of the host care organisation must be obtained before recruitment of participants within that host care organisation.

15.5 Sponsorship

The FURVA trial is being sponsored by Velindre NHS Trust. The Trust shall be responsible for ensuring that the trial is performed in accordance with the following:

- The Medicines for Human Use (Clinical Trials) Regulations 2004 (SI2004/1031) and subsequent amendments.
- Conditions and principles of Good Clinical Practice.
- Declaration of Helsinki (1996) .
- Research Governance Framework for Health and Social Care (Welsh Assembly Government 2009 and Department of Health 2nd July 2005).
- The Data Protection Act 1998.

This material is the property of the Wales Cancer Trials Unit. Do not disclose or use except as authorised.

- The Human Tissue Act 2004.
- Other regulatory requirements as appropriate.

The Sponsor has/will be delegating certain responsibilities to Cardiff University (WCTU), the Chief Investigators, Principal Investigators, host sites and other stakeholder organisations as appropriate in accordance with the relevant agreement that is informed by regulation and study type.

15.6 Indemnity

- Non-negligent harm: This trial is an academic, investigator-led and designed trial sponsored by Velindre NHS Trust and coordinated by the WCTU. The Chief Investigator, local Investigators and WCTU do not hold insurance against claims for compensation for injury caused by participation in a clinical trial and therefore cannot offer any non-negligent harm indemnity. The Association of the British Pharmaceutical Industry (ABPI) guidelines will not apply.
- Negligent harm: In accordance with Technical Note 12 Indemnity for Clinical Research for research Sponsored by a Welsh body, Welsh Risk Pool Services provides indemnity cover against successful negligence claims arising from the management and conduct of the study. Where NHS employees are responsible for the design of a study, indemnity cover will also be provided for negligent harm arising from the study design. Velindre NHS Trust does not accept liability for any breach in the other NHS Organisations duty of care, or any negligence on the part of employees of these NHS Organisations.

15.7 Data protection

The WCTU will act to preserve patient confidentiality and will not disclose or reproduce any information by which participants could be identified, except where specific consent is obtained. This includes collection of NHS number (or equivalent – e.g. CHI number in Scotland), name, date of birth, and postcode to register and trace participants with the NHSIC. This also includes collection of NHS number or equivalent to use NHS data for future research through Cancer Research UK.

Data will be stored in a secure manner and will be registered in accordance with the Data Protection Act 1998. The data custodian and the translational sample custodian for this trial is the interim Director of the WCTU reverting to the Director of WCTU on appointment.

15.8 Finance

The trial is in the NIHR portfolio and has support from both the NCRI Breast Cancer Clinical Studies Group and Advanced Disease Subgroup.

AstraZeneca are co-funding the study with a per-patient payment and also provision of the study drugs. Astra Zeneca sold its Vandetanib business to Genzyme in July 2015. As the FURVA trial uses a combination of Vandetanib and another AZ drug (fulvestrant) Astra Zeneca retained oversight of accountabilities of the trial until its completion with no impact on trial procedures. However, both companies will be jointly responsible for the trial. The WCTU is core funded by CR-UK and these core resources will be used to support this trial. The trial is in the National Cancer Research Institute

This material is the property of the Wales Cancer Trials Unit. Do not disclose or use except as authorised.

(NCRI), NISCHR Portfolio (CSP) and National Institute for Health (NIHR) portfolio. Local NCRN/WCTN/SCRN support should be available at each site taking part to support entry of participants into this trial.

This material is the property of the Wales Cancer Trials Unit. Do not disclose or use except as authorised.

16.0 References

1. http://info.cancerresearchuk.org/cancerstats/types/breast/?script=true accessed 2/7/12.
2. **Early Breast Cancer Trialists' Collaborative Group (EBCTCG), Davies, C., Godwin, J., Gray. R, Clarke, M., Cutter, D., Darby, S., McGale, P., Pan, H. C., Taylor, C., Wang, Y. C., Dowsett, M., Ingle, J. & Peto, R.** (2011). Relevance of breast cancer hormone receptors and other factors to the efficacy of adjuvant tamoxifen: patient-level meta-analysis of randomised trials. *Lancett* 378(9793):771-84.
3. **Adamo, V., Iorfida, M., Montalto, E., Festa, V., Garipoli, C., Scimone, A., Zanghì, M. & Caristi N.** (2007). Overview and new strategies in metastatic breast cancer (MBC) for treatment of tamoxifen-reistant patients. *Annals of Oncology* 18 Supplement 6:vi53-57.
4. **Chia, S., Gradishar, W., Mauriac, L., Bines, J., Amant, F., Federico, M., Fein, L., Romieu, G., Buzdar, A., Roberstson, J. F., Brufsky, A., Possinger, K., Rennie, P., Sapunar, F., Lowe, e. & Piccart, M.** (2008). A double-blind, randomised placebo controlled trial of fulvestrant compared with exemestane after prior nonsteroidal aromatase inhibitor therapy in postmenopausal women with hormone receptor-positive, advanced breast cancer: results from EFECT. *Journal of Clinical Oncology* 26(10):1664-70.
5. **Di Leo, A., Jerusalem, G., Petruzelka, L., Torres, R., Bondarenko, I. N., Khasanov, R., Verhoeven, D., Pedrini, J. L., Smirnova, I., Lichinitser, M. R., Pendergrass, K., Garnett, S., Lindemann, J. P. O., Sapunar, F. & Martin, M.** (2010). Results of the CONFIRM Phase III Trial comparing Fulvestrant 250 mg with Fulvestrant 500 mg in Postmenopausal women with Estrogen-Receptor positive advanced breast cancer. *Journal of Clinical Oncology* 28:4594-4600.
6. **Robertson, J. F., Lindemann, J. P., Llombart-Cussac, A., Rolski, J., Feltl, D., Dewar, J., Emerson, L., Dean, A. & Ellis, M. J.** (2012) Fulvestrant 500 mg versus anastrozole 1 mg for the first-line treatment of advanced breast cancer: follow-up analysis from the randomized 'FIRST' study. *Breast Cancer Research and Treatment* 136(2):503-511.
7. **Osborne, C. K. & Schiff, R.** (2011). Mechanisms of endocrine resistance in breast cancer. *Annual Review of Medicine* 62:233-47.
8. **Hutcheson, I. R., Knowlen, J. M., Jones, H. E., Burmi, R. S., McClelland, R. A., Barrow, D., Gee. J. M. & Nicholson, R. I.** (2006). Inductive mechanisms limiting response to anti-epidermal growth factor receptor therapy. *Endocrine-Related Cancer* 13 Supplement 1:S89-97.
9. **Plaza-Menacho, I., Morandi, A., Robertson, D., Pancholi, S., Drury, S., Dowsett, M., Martin, L. A. & Isacke, C. M.** (2010). Targeting receptor tyrosine kinase RET sensitizes breast cancer cells to tamoxifen treatment and reveals a role for RET in endocrine resistance. *Oncogene* 29(33):4648-4657.
10. **Esseghir, S., Todd, S. K., Hunt, T., Poulsom, R., Plaza-Menacho, I., Reis-Filho, J. S. & Isacke, C. M.** (2007). A role for glial cells derived neurotrophic factor induced expression by inflammatory

This material is the property of the Wales Cancer Trials Unit. Do not disclose or use except as authorised.

cytokines and RET/GFR alpha 1 receptor up-regulation in breast cancer. *Cancer Research* 67(24):11732-11741.

11. **Gustafson, D. L., Bradshaw-Pierce, E.L., Merz, A. L. & Zirrolli, J. A.** (2006). Tissue distribution and metabolism of the tyrosine kinase inhibitor ZD6474 (Zactima) in tumor-bearing nude mice following oral dosing. *Journal of Pharmacology and Experimental Therapeutics* 318:872-880.
12. **Wedge, S. R., Ogilvie, D. J., Dukes, M., Kendrew, J., Chester, R., Jackson, J. A., Boffey, S. J., Valentine, P. J., Curwen, J. O., Musgrove, H. L., Graham, G. A., Hughes, G. D., Thomas, P. D., Stokes, E. S. E., Curry, B., Richmond, G. H. P., Wadsworth, P. F., Bigley, A. L. & Hennequin, L. F.** (2002). ZD6474 inhibits vascular endothelial growth factor signaling, angiogenesis, and tumour growth following oral administration. *Cancer Research* 62:4645.
13. **Vitagliano, D., De Falco, V., Tamburrino, A., Coluzzi, S., Trocone, G., Chiappetta, G., Ciardiello, F., Tortora, G., Fagin, J. A., Ryan, A. J., Carlomagno, F. & Santoro, M.** (2010). The tyrosine kinase inhibitor ZD6474 blocks proliferation of RET mutant medullary thyroid carcinoma cells. *Endocrine-Related Cancer* 18(1):1-11.
14. **Boer, K., Lang, I., Llombart-Cussac, A., Andreasson, I., Vivanco, G. L., Sanders, N., Pover, G. M. & Murray, E.** (2007). Vandetanib with docetaxl as second-line treatment for advanced breast cancer: a double-blind, placebo controlled, randomized phase II study. *San Antonio Breast Cancer Symposium (Dec 13-16, San Antonio)*, Abstract No. 6081.
15. **Clemons, M. J., Cochrane, B., Califaretti, N., Chia, K. L., Dent, R. A., Song, X., Robidoux, A., Pond, G. R., Parpia, S., Pritchard, K. I. & Levine, M. N.** (2013). Randomized phase II placebo-controlled trial of fulvestrant plus vandetanib in postmenopausal women with bone only or bone predominant, hormone receptor-positive metastatic breast cancer (MBC): OCOG Zamboney study, NCT00811369. *ASCO Annual Meeting* Abstract No. 574.

This material is the property of the Wales Cancer Trials Unit. Do not disclose or use except as authorised.

APPENDIX 1: RECIST v1.1

INTRODUCTION

This appendix details the implementation of RECIST 1.1 Guidelines (www.recist.com) for the FURVA study with regards to Investigator assessment of tumour burden including protocol-specific requirements for this study.

DEFINITION OF MEASURABLE, NON-MEASURABLE, TARGET AND NON-TARGET LESIONS

Measurable:

A lesion, not previously irradiated, that can be accurately measured at baseline as ≥10 mm in the longest diameter (except lymph nodes which must have short axis ≥15 mm) with CT or MRI and which is suitable for accurate repeated measurements.

Non-measurable:

- All other lesions, including small lesions (longest diameter <10 mm or pathological lymph nodes with ≥10 to <15 mm short axis at baseline*).
- Truly non-measurable lesions include the following: bone lesions, leptomeningeal disease, ascites, pleural / pericardial effusion, inflammatory breast disease, lymphangitic involvement of skin or lung, abdominal masses/abdominal organomegaly identified by physical examination that is not measurable by CT or MRI.
- Previously irradiated lesions.**
- Skin lesions assessed by clinical examination.***
- Brain metastasis.***

* Nodes with <10 mm short axis are considered non-pathological and should not be recorded or followed as NTL.

** Localised post-radiation changes which affect lesion sizes may occur. Therefore, lesions that have been previously irradiated will not be considered measurable and must be selected as Non-Target Lesions (NTL) at baseline and followed up as part of the NTL assessment.

*** Skin lesions assessed by clinical examination and brain lesions are considered as NTL.

Special Cases:

- Lytic bone lesions or mixed lytic–blastic lesions, with identifiable soft tissue components, can be considered measurable if the soft tissue component meets the definition of measurability. Blastic lesions are considered non-measurable.

This material is the property of the Wales Cancer Trials Unit. Do not disclose or use except as authorised.

- Cystic metastases can be considered measurable lesions if they meet the criteria for measurability from a radiological point of view, but if non-cystic lesions are present in the same patient; these should be selected as target lesions.

Target lesions:

A maximum of 5 measurable lesions (with a maximum of 2 lesions per organ), representative of all lesions involved suitable for accurate repeated measurement, should be identified as target lesions (TL) at baseline.

Non-Target lesions:

All other lesions (or sites of disease) not recorded as TL should be identified as NTL at baseline.

METHODS OF ASSESSMENT

The same method of assessment and the same technique should be used to characterise each identified and recorded lesion at baseline and during follow-up visits.

A summary of the methods to be used for RECIST assessment is provided below and those excluded from tumour assessments for this study are highlighted, with the rationale provided.

Table 1: Summary of Methods of Assessment

Target Lesions	Non-Target Lesions	New Lesions
CT (preferred)	CT (preferred)	CT (preferred)
MRI	MRI	MRI
	Clinical examination	Clinical examination
	X-ray, Chest x-ray	X-ray, Chest x-ray
		Ultrasound
		Bone Scan
		FDG-PET

1.1 CT and MRI

CT and MRI are generally considered to be the best currently available and reproducible methods to measure TL selected for response assessment and to assess NTL and identification of any new lesions.

1.2 Clinical examination

In the FURVA study, clinical examination will not be used for assessment of TL. Clinically detected lesions can be selected as target lesions if they are assessed by CT or MRI scans. Clinical examination can be used to assess NTL and to identify the presence of new lesions.

CONFIDENTIAL

This material is the property of the Wales Cancer Trials Unit. Do not disclose or use except as authorised.

1.3 X-ray

1.3.1 Chest X-ray

Chest x-ray assessment will not be used for assessment of TL as they will be assessed by CT examination or MRI examination. Chest X-ray can, however, be used to assess NTL and to identify the presence of new lesions.

1.3.2 Plain X-ray

Plain x-ray may be used as a method of assessment for bone NTL and to identify the presence of new bone lesions.

1.4 Ultrasound

Ultrasound examination will not be used for assessment of TL and NTL as it is not a reproducible method, does not provide an accurate assessment of tumour size and it is subjective and operator dependent. Ultrasound examination can, however, be used to identify the presence of new lesions. If new clinical symptoms occur and an ultrasound is performed then new lesions should be confirmed by CT or MRI examination.

1.5 Endoscopy and laparoscopy

Endoscopy and laparoscopy will not be used for tumour assessments as they are not validated in the context of tumour assessment.

1.6 Tumour markers

Tumour markers will not be used for tumour response assessments as per RECIST 1.1.

1.7 Cytology and histology

Histology will not be used as part of the tumour response assessment as per RECIST 1.1.

Cytological confirmation of the neoplastic origin of any effusion that appears or worsens during treatment is required when the measurable tumour has met criteria for response or stable disease. In such circumstances, the cytology is necessary to differentiate between response / stable disease (an effusion may be a side effect of the treatment) and progressive disease (if the neoplastic origin of the fluid is confirmed). Where cytology findings are not available, any effusion that significantly worsens (from trace to large) or appearance of clinically significant effusion (requiring change in drug therapy) during the study treatment will be considered to be progression of NTL, or disease progression due to new lesions.

1.8 Isotopic bone scan

Isotopic bone scans are not a protocol requirement for FURVA, but clinicians may use these if necessary for clinical decision making.

Bone lesions identified on an isotopic bone scan at baseline and confirmed by CT, MRI or X-ray at baseline should be recorded as NTL and followed by the same method as per baseline assessment.

Isotopic bone scans may be used as a method of assessment to identify the presence of new bone lesions at follow-up visits. New lesions will be recorded where a positive hot-spot that was not present on the

CONFIDENTIAL

This material is the property of the Wales Cancer Trials Unit. Do not disclose or use except as authorised.

baseline bone scan assessment is identified on a bone scan performed at any time during the study. The Investigator should consider the positive hot-spot to be a significant new site of malignant disease and represent true disease progression in order to record the new lesion. Confirmation by CT, MRI and x-ray is recommended where bone scan findings are equivocal.

1.9 FDG-PET scan

FDG-PET scans are not a protocol requirement for FURVA, but clinicians may use these if necessary for clinical decision making.

FDG-PET scans may be used as a method for identifying new lesions, according with the following algorithm: New lesions will be recorded where there is positive FDG uptake* not present on baseline FDG-PET scan or in a location corresponding to a new lesion on CT/MRI at the same follow-up visit. If there is no baseline FDG-PET scan available, and no evidence of new lesions on CT/MRI scans then follow-up CT/MRI assessments should be continued, scheduled as per protocol or clinically indicated, in order to confirm new lesions.

* A positive FDG-PET scan lesion should be reported only when an uptake greater than twice that of the surrounding tissue is observed.

TUMOUR RESPONSE EVALUATION

1.10 Schedule of evaluation

Baseline assessments should encompass all areas of known predilection for metastases in the disease under evaluation and should additionally investigate areas that may be involved based on signs and symptoms of individual patients and should be performed no more than 28 days before the start of study treatment. Follow-up assessments will be performed every 12 weeks ($\pm$7 days) after start of treatment until objective disease progression as defined by RECIST 1.1. Any other sites at which new disease is suspected should also be adequately imaged at follow-up.

If an unscheduled assessment was performed and the patient has not progressed, every attempt should be made to perform the subsequent assessments at their scheduled visits. This schedule is to be followed in order to minimise any unintentional bias caused by some patients being assessed at a different frequency than other patients.

1.11 Target lesions (TL)

1.11.1 Documentation of target lesions

A maximum of 5 measurable lesions, with a maximum of 2 lesions per organ (including lymph nodes), representative of all lesions involved should be identified as TL at baseline. Target lesions should be selected on the basis of their size (longest diameter for non-nodal lesions or short axis for nodal lesions), but in addition should be those that lend themselves to reproducible repeated measurements. It may be the case that, on occasion, the largest lesion does not lend itself to reproducible measurement in which circumstance the next largest lesion, which can be measured reproducibly, should be selected.

The site and location of each TL should be documented as well as the longest diameter for non-nodal lesions (or short axis for lymph nodes). All measurements should be recorded in millimeters. At baseline the sum of the diameters for all TL will be calculated and reported as the baseline sum of diameters. At

This material is the property of the Wales Cancer Trials Unit. Do not disclose or use except as authorised.

follow-up visits the sum of diameters for all TL will be calculated and reported as the follow-up sum of diameters.

Special cases:

- For TL measurable in 2 or 3 dimensions, always report the longest diameter. For pathological lymph nodes measurable in 2 or 3 dimensions, always report the short axis.
- If the CT/MRI slice thickness used is >5 mm, the minimum size of measurable disease at baseline should be twice the slice thickness of the baseline scan.
- If a lesion has completely disappeared, the longest diameter should be recorded as 0 mm.
- If a TL splits into two or more parts, then record the sum of the diameters of those parts.
- If two or more TL merge then the sum of the diameters of the combined lesion should be recorded for one of the lesions and 0 mm recorded for the other lesion(s).
- If a TL is believed to be present and is faintly seen but too small to measure, a default value of 5mm should be assigned. If an accurate measure can be given, this should be recorded, even if it is below 5 mm.
- If a TL cannot be measured accurately due to it being too large, provide an estimate of the size of the lesion.
- When a TL has had any intervention e.g. radiotherapy, embolisation, surgery etc., during the study, the size of the TL should still be provided where possible.

1.11.2 Evaluation of target lesions

This section provides the definitions of the criteria used to determine objective tumour visit response for TL.

Table 2: Evaluation of target lesions

Complete Response (CR)	Disappearance of all target lesions since baseline. Any pathological lymph nodes selected as target lesions must have a reduction in short axis to <10 mm.
Partial Response (PR)	At least a 30% decrease in the sum of the diameters of TL, taking as reference the baseline sum of diameters
Stable Disease (SD)	Neither sufficient shrinkage to qualify for PR nor sufficient increase to qualify for PD
Progressive Disease (PD)	At least a 20% increase in the sum of diameters of target lesions, taking as reference the smallest sum on study (this includes the baseline sum if that is the smallest on study). In addition to the relative increase of 20%, the sum must also demonstrate an absolute increase of at least 5mm.

This material is the property of the Wales Cancer Trials Unit. Do not disclose or use except as authorised.

Not Evaluable (NE)	Only relevant if any of the target lesions were not assessed or not evaluable or had a lesion intervention at this visit. Note: If the sum of diameters meets the progressive disease criteria, progressive disease overrides not evaluable as a target lesion response

1.12 Non-Target lesions (NTL)

1.12.1 Evaluation of non-target lesions

All other lesions (or sites of disease) not recorded as TL should be identified as NTL at baseline. Measurements are not required for these lesions, but their status should be followed at subsequent visits. At each visit an overall assessment of the NTL response should be recorded by the Investigator. This section provides the definitions of the criteria used to determine and record overall response for NTL at the investigational site at each visit.

Table 3: Evaluation of Non-Target Lesions

Complete Response (CR)	Disappearance of all non-target lesions since baseline. All lymph nodes must be non-pathological in size (< 10 mm short axis).
Non CR/Non PD	Persistence of one or more NTL
Progression (PD)	Unequivocal progression of existing non-target lesions. Unequivocal progression may be due to an important progression in one lesion only or in several lesions. In all cases the progression MUST be clinically significant for the physician to consider changing (or stopping) therapy.
Not Evaluable (NE)	Only relevant when one or some of the non-target lesions were not assessed and, in the Investigator's opinion, they are not able to provide an evaluable overall non-target lesion assessment at this visit.<br>Note: For patients without target lesions at baseline, this is relevant if any of the non-target lesions were not assessed at this visit and the progression criteria have not been met.

To achieve ‘unequivocal progression’ on the basis of non-target lesions, there must be an overall level of substantial worsening in non-target disease such that, even in presence of SD or PR in target lesions, the overall tumour burden has increased sufficiently to merit discontinuation of therapy. A modest ‘increase’ in the size of one or more non-target lesions is usually not sufficient to qualify for unequivocal progression status.

1.13 New Lesions

Details of any new lesions will also be recorded with the date of assessment. The presence of one or more new lesions is assessed as progression.

A lesion identified at a follow up assessment in an anatomical location that was not scanned at baseline is considered a new lesion and will indicate disease progression.

CONFIDENTIAL

This material is the property of the Wales Cancer Trials Unit. Do not disclose or use except as authorised.

The finding of a new lesion should be unequivocal: i.e. not attributable to differences in scanning technique, change in imaging modality or findings thought to represent something other than tumour.

If a new lesion is equivocal, for example because of its small size, the treatment and tumour assessments should be continued until the new lesion has been confirmed. If repeat scans confirm there is a new lesion, then the progression date should be declared using the date of the initial scan.

1.14 Symptomatic deterioration

Symptomatic deterioration is not a descriptor of an objective response: it is a reason for stopping study therapy.

Patients with 'symptomatic deterioration' requiring discontinuation of treatment without objective evidence of disease progression at that time should continue to undergo tumour assessments where possible until objective disease progression is observed.

1.15 Evaluation of Overall Visit Response

The overall visit response will be derived using the algorithm shown in Table 4.

Table 4: Overall Visit Response

Target lesions	Non-Target lesions	New Lesions	Overall response
CR	CR	No	CR
CR	NA	No	CR
NA	CR	No	CR
CR	Non CR/Non PD	No	PR
CR	NE	No	PR
PR	Non PD or NE	No	PR
SD	Non PD or NE	No	SD
NA	Non CR/Non PD	No	SD (Non CR/Non PD)
NE	Non PD or NE	No	NE
NA	NE	No	NE
PD	Any	Yes or No	PD
Any	PD	Yes or No	PD
Any	Any	Yes	PD

CR = complete response, PR = partial response, SD = stable disease, PD = progressive disease, NE = not evaluable, NA = not applicable (only relevant if there were no TL/NTLs at baseline).

This material is the property of the Wales Cancer Trials Unit. Do not disclose or use except as authorised.

SPECIFICATIONS FOR RADIOLOGICAL IMAGING

These notes are recommendations for use in clinical studies. The use of standardised protocols for CT and MRI allows comparability both within and between different studies, irrespective of where the examination has been undertaken.

1.16 CT Scan

CT scans of the chest, abdomen, and pelvis should be contiguous throughout all the anatomic region of interest.

The most critical CT image acquisition parameters for optimal tumour evaluation using RECIST 1.1 are anatomic coverage, contrast administration, slice thickness, and reconstruction interval.

a. Anatomic coverage: Optimal anatomic coverage for most solid tumours is the chest, abdomen and pelvis. Coverage should encompass all areas of known predilection for metastases in the disease under evaluation and should additionally investigate areas that may be involved based on signs and symptoms of individual patients. Because a lesion later identified in a body part not scanned at baseline would be considered as a new lesion representing disease progression, careful consideration should be given to the extent of imaging coverage at baseline and at subsequent follow-up time points. This will enable better consistency not only of tumour measurements but also identification of new disease.

b. IV contrast administration: Optimal visualisation and measurement of metastases in solid tumours requires consistent administration (dose and rate) of IV contrast as well as timing of scanning. Typically, most abdominal imaging is performed during the portal venous phase and (optimally) about the same time frame after injection on each examination. An adequate volume of a suitable contrast agent should be given so that the metastases are demonstrated to best effect and a consistent method is used on subsequent examinations for any given patient. It is very important that the same technique be used at baseline and on follow- up examinations for a given patient. For patients who develop contraindications to contrast after baseline contrast CT is done, the decision as to whether non-contrast CT or MRI (enhanced or non-enhanced) should be performed should also be based on the tumour type, anatomic location of the disease and should be optimised to allow for comparison to the prior studies if possible. Each case should be discussed with the radiologist to determine if substitution of these other approaches is possible and, if not, the patient should be considered not evaluable from that point forward. Care must be taken in measurement of target lesions on a different modality and interpretation of non-target disease or new lesions, since the same lesion may appear to have a different size using a new modality. Oral contrast is recommended to help visualise and differentiate structures in the abdomen.

If iodine contrast media is medically contraindicated at baseline or at any time during the course of the study then the recommended methods are: CT thoracic examination without contrast and abdominal and pelvis MRI with contrast. If MRI cannot be performed then CT without intravenous contrast is an option for the thorax, abdomen and pelvis examination. For brain lesions assessment, MRI is the preferred method.

c. Slice thickness and reconstruction interval: It is recommended that CT scans be performed at 5mm contiguous slice thickness and this guideline presumes a minimum 5 mm thickness in recommendations for measurable lesion definition. Exceptionally, particular institutions may perform medically acceptable scans at slice thicknesses greater than 5 mm. If this occurs, the minimum size of measurable lesions at baseline should be twice the slice thickness of the baseline scans.

CONFIDENTIAL

This material is the property of the Wales Cancer Trials Unit. Do not disclose or use except as authorised.

All window settings should be included in the assessment, particularly in the thorax where lung and soft tissue windows should be considered. When measuring lesions, the TL should be measured on the same window setting for repeated examinations throughout the study. All images from each examination should be included in the assessment and not "selected" images of the apparent lesion.

1.17 MRI Scan

MRI has excellent contrast, spatial and temporal resolution; however, there are many image acquisition variables involved in MRI, which greatly impact image quality, lesion conspicuity and measurement. Furthermore, the availability of MRI is variable globally. The modality used at follow-up should be the same as was used at baseline and the lesions should be measured/assessed on the same pulse sequence. Generally, axial imaging of the abdomen and pelvis with T1 and T2 weighted imaging along with gadolinium-enhanced imaging should be performed. The field of view, matrix, number of excitations, phase encode steps, use of fat suppression and fast sequences should be optimised for the specific body part being imaged as well as the scanner used. It is beyond the scope appendix to prescribe specific MRI pulse sequence parameters for all scanners, body parts and diseases. Ideally, the same type of scanner should be used and the image acquisition protocol should be followed as closely as possible to prior scans. Body scans should be performed with breath-hold scanning techniques if possible.

For these reasons, CT is the imaging modality of choice.

1.18 FDG-PET scans

FDG-PET has gained acceptance as a valuable tool for detecting, staging and restaging several malignancies. If FDG-PET scans are included in a protocol, an FDG uptake period of 60 min prior to imaging has been decided as the most appropriate for imaging of patients with malignancy. Whole-body acquisition is important since this allows for sampling of all areas of interest and can assess if new lesions have appeared thus determining the possibility of interval progression of disease. Images from the base of the skull to the level of the mid-thigh should be obtained 60 min post injection. PET camera specifications are variable and manufacturer specific, so every attempt should be made to use the same scanner, or the same model scanner, for serial scans on the same patient. Whole-body acquisitions can be performed in either 2- or 3-dimensional mode with attenuation correction, but the method chosen should be consistent across all patients and serial scans in the clinical trial.

1.18.1 PET/CT scans

At present, low dose or attenuation correction CT portions of a combined PET–CT are of limited use in anatomically based efficacy assessments and it is therefore suggested that they should not be substituted for dedicated diagnostic contrast enhanced CT scans for tumour measurements by RECIST 1.1. In exceptional situations, if a site can document that the CT performed as part of a PET–CT is of identical diagnostic quality to a diagnostic CT (with IV and oral contrast) then the CT portion of the PET–CT can be used for RECIST measurements. However, this is not recommended because the PET portion of the CT introduces additional data that may bias an investigator if it is not routinely or serially performed.

This material is the property of the Wales Cancer Trials Unit. Do not disclose or use except as authorised.

REFERENCES

Eisenhauer et al 2009

Eisenhauer EA, Therasse P, Bogaerts J, Schwartz LH, Sargent D, Ford R. New response evaluation criteria in solid tumours: Revised RECIST guideline (version 1.1). Eur J Cancer 45 (2009) 228-247

This material is the property of the Wales Cancer Trials Unit. Do not disclose or use except as authorised.

APPENDIX 2: New York Heart Association (NYHA) classification of heart disease

**NYHA Class**	**Symptoms**
I	No symptoms and no limitation in ordinary physical activity, e.g. shortness of breath when walking, climbing stairs etc.
II	Mild symptoms (mild shortness of breath and/or angina) and slight limitation during ordinary activity.
III	Marked limitation in activity due to symptoms, even during less-than-ordinary activity, e.g. walking short distances (20–100 m). Comfortable only at rest.
IV	Severe limitations. Experiences symptoms even while **at rest**. Mostly bedbound patients.

References

New York Heart Association 1994

The Criteria Committee of the New York Heart Association: Nomenclature and Criteria for Diagnosis of Diseases of the Heart and Great Vessels. 9th edition. Boston, MA: Little, Brown & Co; 1994:253-256.

This material is the property of the Wales Cancer Trials Unit. Do not disclose or use except as authorised.

APPENDIX 3: A guide to performing causality assessments

The following factors should be considered when deciding if there is a "reasonable possibility" that an SAE may have been caused by the drug.

- Time course. Exposure to suspect drug. Has the subject actually received the suspect drug? Did the SAE occur in a reasonable temporal relationship to the administration of the suspect drug?
- Consistency with known drug profile. Was the AE consistent with the previous knowledge of the suspect drug (pharmacology and toxicology) or drugs of the same pharmacological class? Or could the SAE be anticipated from its pharmacological properties?
- Dechallenge experience. Did the SAE resolve or improve on stopping or reducing the dose of the suspect drug?
- No alternative cause. The SAE cannot be reasonably explained by another aetiology such as the underlying disease, other drugs, other host or environmental factors.
- Laboratory tests. A specific laboratory investigation (if performed) has confirmed the relationship?
- A "reasonable possibility" could be considered to exist for an SAE where one or more of these factors exist.

In contrast there would not be a "reasonable possibility" of causality if none of the above criteria apply or where there is evidence of exposure and a reasonable time course but any dechallenge (if performed) is negative or ambiguous or there is another more likely cause of the SAE.

In difficult cases other factors should be considered such as:

Is this a recognised feature of overdose of the drug?

Is there a known mechanism?

Ambiguous cases should be considered as being a "reasonable possibility" of causal relationship unless further evidence becomes available to refute this. Causal relationship in cases where the disease under study has deteriorated due to lack of effect should be classified as no reasonable possibility.

CONFIDENTIAL

This material is the property of the Wales Cancer Trials Unit. Do not disclose or use except as authorised.

APPENDIX 4: Cockcroft and Gault Formula to calculate GFR

$$\text{Women: CrCl [ml/min]} = \frac{(140\text{-age}) \times \text{weight [kg]} \times 1.04}{\text{SeCr [}\mu\text{mol/l]}}$$

This material is the property of the Wales Cancer Trials Unit. Do not disclose or use except as authorised.

WALES CANCER TRIALS UNIT

Contact Details:

Wales Cancer Trials Unit
Centre for Trials Research
College of Biomedical & Life Sciences
Cardiff University
6th Floor
Neuadd Meirionnydd
Heath Park
Cardiff
CF14 4YS

General Telephone Number: 029 2068 7500
General Fax: 029 20687501

Email:
FURVA@cardiff.ac.uk

Visit our website:
www.cardiff.ac.uk/centre-for-trials-research
